# Palladium-Catalyzed Carbonylative Fluoroalkylation of 1,3-Enynes to Access Allenyl Primary Amides

**DOI:** 10.1021/acs.orglett.5c03213

**Published:** 2025-08-20

**Authors:** Chang-Sheng Kuai, Ru-Han A, Zhi-Peng Bao, Xiao-Feng Wu

**Affiliations:** † Dalian National Laboratory for Clean Energy, Dalian Institute of Chemical Physics, Chinese Academy of Sciences, Dalian 116023, China; ‡ University of Chinese Academy of Sciences, Beijing 100049, China; § Leibniz-Institut für Katalyse e. V., Albert-Einstein-Straβe 29a, 18059 Rostock, Germany

## Abstract

A palladium-catalyzed difunctional fluoroalkylative carbonylation of 1,3-enynes with fluoroalkyl halides and ammonium iodide has been developed, providing rapid access to fluoroalkylated allenyl primary amides with a broad substrate scope, high chemo- and regioselectivity, and good functional group tolerance.

## Graphical abstract

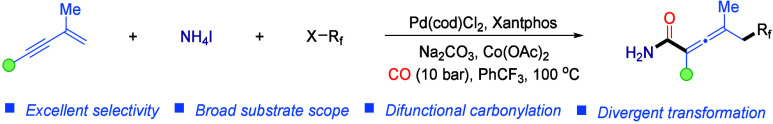

Primary amines, as structurally simple yet functionally diverse nitrogen-containing compounds, are widely distributed in natural products, pharmaceuticals, agrochemicals, and functional materials.^1^ They not only play pivotal roles within molecular architectures but also serve as key intermediates in multistep organic synthesis.^2^ Accordingly, the development of efficient, selective, and broadly applicable methods for the synthesis of primary amines is of great significance across the fields of organic synthesis, biomedical research, and materials science.

Among the various synthetic strategies, transition-metal-catalyzed carbonylation reactions have emerged as exceptionally powerful tools, offering one of the most direct and efficient methods for the construction of primary amides because of their high atom economy and excellent functional group compatibility. For example, early studies primarily employed aryl (pseudo)­halides as electrophiles in Heck-type carbonylation reactions, leading to the development of various synthetic methods for aryl primary amides (Scheme 1A).^3^ Subsequently, elegant work by the groups of Huang,^4^ Liu,^5^ and others^6^ not only expanded the scope to include the hydroamidation of alkenes and alkynes but also circumvented the use of weakly coordinating, highly corrosive, and hazardous gaseous ammonia by employing surrogate ammonia sources, thereby enabling efficient access to a broad range of alkyl and allyl primary amides (Scheme 1A).

**Scheme 1:**
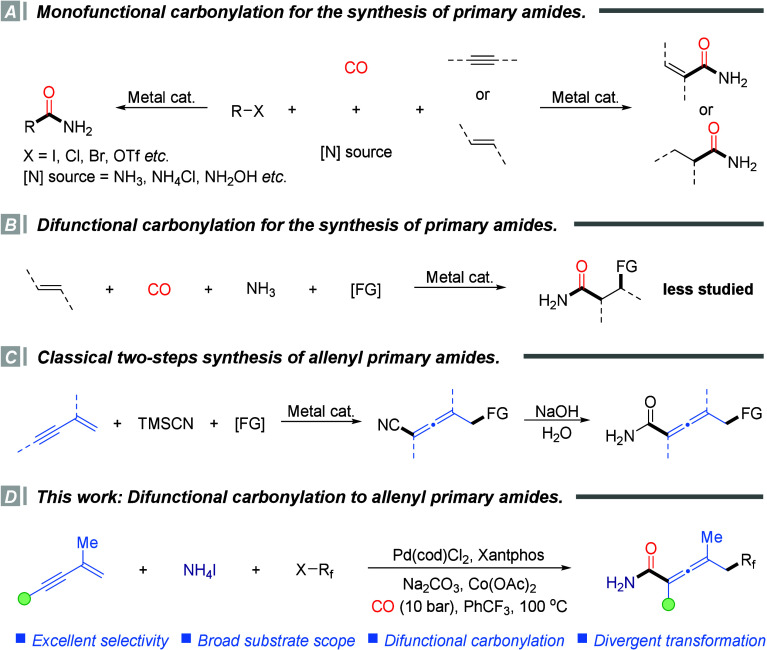
Catalytic Carbonylation Strategies for the Synthesis of Primary Amides

Despite these advances, most of these strategies remain inherently limited to monofunctionalization, typically affording structurally simple products. Such limitations restrict the efficient construction of molecular complexity and functional diversity in a single transformation. To overcome these challenges, multicomponent difunctional carbonylation reactions have attracted a growing interest. These reactions enable the simultaneous installation of two distinct functional groups in a single step, providing a highly atom- and step-economical approach to structurally complex and functionally rich molecules. In this context, both Beller’s group^7^ and our own^8^ have independently developed difunctional carbonylation strategies for alkenes, enabling the efficient synthesis of structurally diverse alkyl primary amides and thereby demonstrating the feasibility and synthetic potential of this approach (Scheme 1B). However, most reported examples have predominantly focused on the formation of secondary and tertiary amides, while the direct synthesis of primary amides has remained relatively underexplored. In addition, the substrate scope has been largely limited to simple alkyl frameworks, highlighting the urgent need for more general and functionally enriched carbonylation strategies capable of constructing structurally complex and diverse primary amide scaffolds.

On the other hand, allenes are versatile and reactive intermediates widely present in natural products, pharmaceuticals, and functional materials.^9^ The integration of allene scaffolds with primary amide groups within a single molecular framework not only expands the structural diversity of nitrogen-containing allenes but also provides valuable platforms for drug discovery, molecular probes, and functional materials (Scheme 1C). To date, the synthesis of allenyl primary amides has predominantly relied on a two-step sequence involving difunctional cyanation of 1,3-enynes followed by hydrolysis.^10^ Although effective, this approach suffers from poor atom economy and step inefficiency, failing to meet the growing demand for streamlined and sustainable synthetic methods. Therefore, the development of one-step difunctional carbonylation protocols that directly assemble allenyl primary amides remains a highly desirable yet unmet challenge.

Building on our continued interest in carbonylation chemistry,^11^ we herein report a palladium-catalyzed difunctional carbonylation of 1,3-enynes that enables the efficient one-step construction of allenyl primary amides from readily available substrates under mild conditions (Scheme 1D). Notably, the concurrent incorporation of a fluoroalkyl group into the allenyl primary amide framework not only significantly enhances the electronic properties and lipophilicity of the products but also improves their metabolic stability and pharmacokinetic profiles, further expanding the application potential of allenyl primary amides in fields such as medicine and materials science.

At the outset, we initiated our investigation using 1,3-enyne **1a**, ammonium iodide (**2a**), and trifluoroiodomethane (**3a**) as model substrates (Table 1). The reaction was carried out under 10 bar CO at 100 °C, employing Pd­(cod)­Cl_2_ as the catalyst precursor, cobalt­(II) acetate as an additive, and Na_2_CO_3_ as the base with trifluorotoluene as the solvent. We first examined the influence of ligands on the reaction outcome (Table 1, entries 1–8). Under monodentate phosphine conditions, the use of triphenylphosphine delivered the desired product **4a** in 4% yield (Table 1, entry 2). Although the efficiency was low, this result validated the feasibility of the proposed reaction strategy. Encouraged by this initial success, we next evaluated a series of bidentate phosphine ligands, focusing on the impact of their bite angles on the catalytic activity. Notably, BINAP, dppe, dppp, and dppf failed to promote the transformation (Table 1, entries 3–6). In sharp contrast, Xantphos proved to be highly effective, affording the target product in 64% yield (Table 1, entry 8). These findings underscore the critical role of ligand geometry in facilitating the tandem difunctional carbonylation process. We next investigated the influence of Lewis acid additives on the reaction outcome. In the absence of cobalt­(II) acetate, the reaction yield sharply decreased to 33% (Table 1, entry 9), indicating its promoting role in the transformation. We then screened other types of Lewis acidic metal salts, including CuBr_2_, MgCl_2_, Fe­(OTf)_3_, and CeCl_3_ (Table 1, entries 10–13). However, under standard conditions, Co­(OAc)_2_ consistently exhibited superior activity, outperforming all other additives tested. Subsequently, we examined the effect of Co­(OAc)_2_ loading on the reaction efficiency. Varying the amount of this additive, either increasing or decreasing from the standard, failed to further enhance the yield (Table 1, entries 14 and 15). Finally, we evaluated the impact of the solvent polarity on the reaction efficiency. A clear trend was observed, wherein increasing solvent polarity led to diminished reactivity. Among the solvents tested, trifluorotoluene proved to be optimal, outperforming other nonpolar solvents (Table 1, entries 16–20). With regard to the amine source, NH_4_Cl, (NH_4_)_2_CO_3_, and NH_4_HCO_3_ were also tested but led to low or no desired amide product.

**Table 1:**
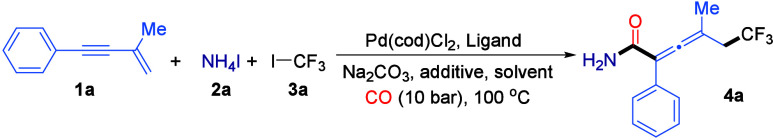
Optimization of the Reaction Conditions^a^

entry	ligand	additive	solvent	yield of **4a** (%)^b^
1	PCy_3_	Co(OAc)_2_	PhCF_3_	ND
2	PPh_3_	Co(OAc)_2_	PhCF_3_	4
3	BINAP	Co(OAc)_2_	PhCF_3_	ND
4	DPPE	Co(OAc)_2_	PhCF_3_	ND
5	DPPP	Co(OAc)_2_	PhCF_3_	ND
6	DPPF	Co(OAc)_2_	PhCF_3_	ND
7	Dpephos	Co(OAc)_2_	PhCF_3_	5
8	Xantphos	Co(OAc)_2_	PhCF_3_	64
9	Xantphos	–	PhCF_3_	33
10	Xantphos	CuBr_2_	PhCF_3_	ND
11	Xantphos	MgCl_2_	PhCF_3_	52
12	Xantphos	Fe(OTf)_3_	PhCF_3_	58
13	Xantphos	CeCl_3_	PhCF_3_	28
14	Xantphos	Co(OAc)_2_	PhCF_3_	55^c^
15	Xantphos	Co(OAc)_2_	PhCF_3_	60^d^
16	Xantphos	Co(OAc)_2_	toluene	53
17	Xantphos	Co(OAc)_2_	DCE	39
18	Xantphos	Co(OAc)_2_	THF	16
19	Xantphos	Co(OAc)_2_	DMF	ND
20	Xantphos	Co(OAc)_2_	DMSO	ND

a Reaction conditions: **1a** (0.1 mmol), **2a** (0.15 mmol), **3a** (0.3 mmol), Pd­(cod)­Cl_2_ (5 mol %), ligand (10 mol % for mono-P; 5 mol % for bis-P), Na_2_CO_3_ (0.3 mmol), additive (50 mol %), CO (10 bar), PhCF_3_ (1.0 mL), 100 °C, 18 h.

b Determined by GC using *n*-hexadecane as the internal standard.

c Co­(OAc)_2_ (10 mol %).

d Co­(OAc)_2_ (100 mol %).

To evaluate the generality of this palladium-catalyzed tandem difunctional carbonylation reaction, we next examined the substrate scope of 1,3-enynes under the optimized catalytic conditions (Scheme 2). We began by exploring a series of aryl-substituted 1,3-enynes bearing a range of electronic and steric substituents. Enynes bearing electron-donating groups such as Me and OMe, halogens (F, Cl, and Br), and electron-withdrawing groups, including trifluoromethyl and ester groups, were well-tolerated, affording the corresponding allenyl primary amides in moderate to good yields (**4a**–**4h**). Notably, the bromo-substituted aryl enyne delivered the desired product in a slightly reduced yield of 29% (**4f**), which may be attributed to undesired side reactions such as oxidative addition of the aryl–Br bond under the palladium-catalyzed conditions. In contrast, enynes bearing *ortho* substituents such as a methyl group furnished only trace amounts of product, likely due to steric hindrance destabilizing the key allenylpalladium intermediate and impeding the catalytic cycle. A *m*-fluoro-substituted enyne exhibited good reactivity, furnishing the target product in 55% yield (**4j**). Enynes bearing extended aromatic systems, such as a naphthyl group, also participated efficiently in the transformation, affording the product in 80% yield (**4k**). Moreover, the reaction was compatible with heteroaryl systems, as demonstrated by the successful transformation of a thiophenyl-substituted 1,3-enyne into the corresponding product (**4l**). Encouraged by these results, we further explored alkyl-substituted 1,3-enynes, including substrates with linear alkyl chains such as phenylpropyl and octyl groups as well as ones bearing heteroatom-based functional groups, such as a silyl ether (OTBS). Gratifyingly, all of these substrates underwent smooth conversion under the standard conditions, affording the corresponding fluoroalkylated allenyl primary amides in synthetically useful to high yields (**4m**–**4o**). These results underscore the broad functional group tolerance and synthetic applicability of this catalytic system. However, no desired product could be detected when the methyl group at the 2-position of the enyne was replaced with a −CF_3_ or Ph group.

**Scheme 2:**
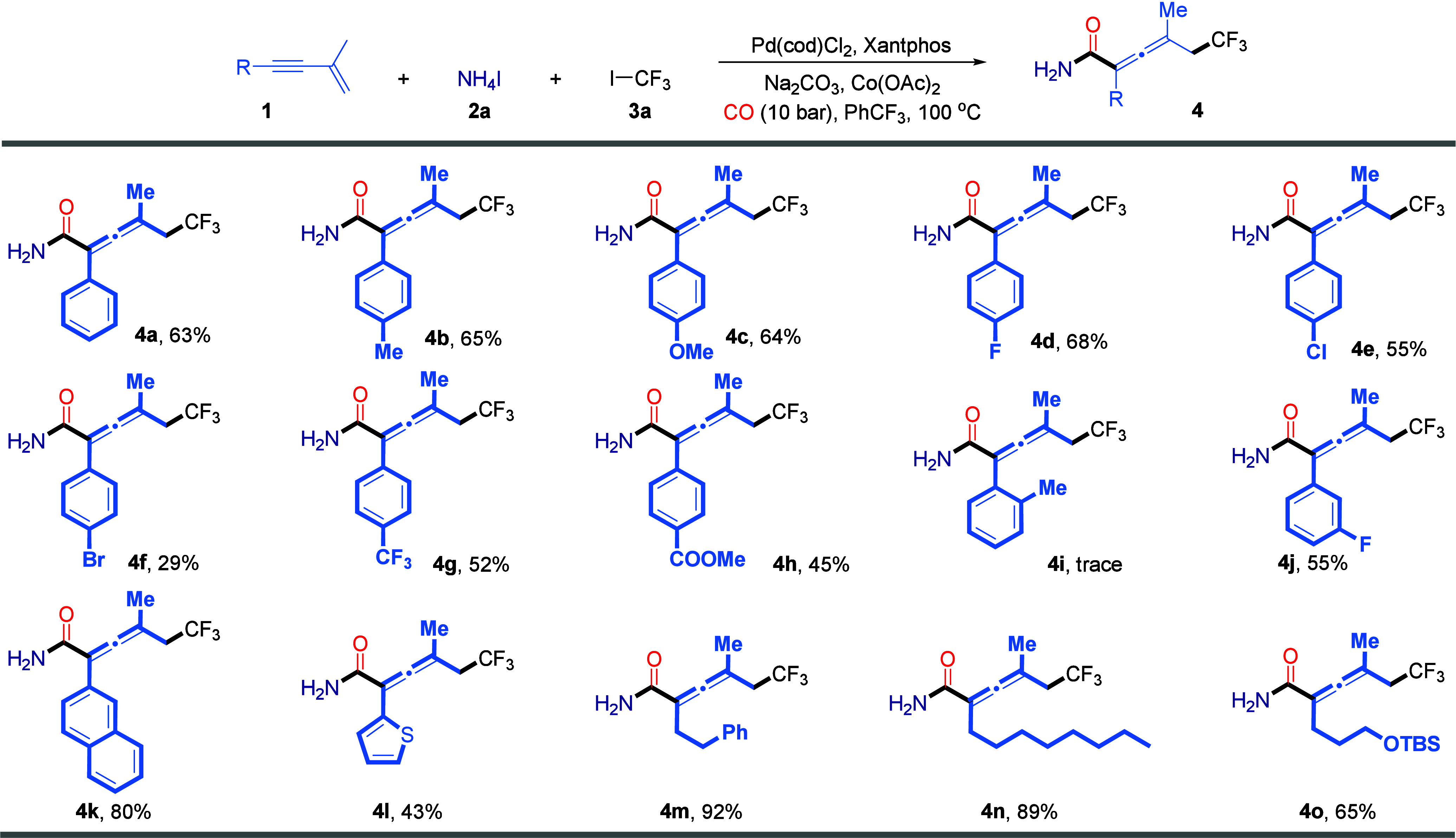
Scope of 1,3-Enynes^a^ ^,^ ^b^

a Reaction conditions: **1a** (0.1 mmol), **2a** (0.15 mmol), **3a** (0.3 mmol), Pd­(cod)­Cl_2_ (5 mol %), Xantphos (5 mol %), Na_2_CO_3_ (0.3 mmol), Co­(OAc)_2_ (50 mol %), CO (10 bar), PhCF_3_ (1.0 mL), 100 °C, 18 h.
b Isolated yields are reported.

We further investigated the scope of the fluoroalkyl halides in this transformation (Scheme 3). In the examination of perfluoroalkyl halides, we observed that iodides were significantly less reactive than their bromide counterparts (**4p**, **4q**), likely due to the generation rate of fluoroalkyl radicals, which affects the overall efficiency and compatibility of the reaction. This trend was further supported by the results obtained with bromodifluoroacetate and iododifluoroacetate, which gave the corresponding product **4r** in 92% and 42% yield, respectively. When the number of fluorine atoms was reduced, as in the case of monofluoroacetate, the reaction yield dropped sharply to 29%, highlighting the essential role of fluorine atoms in stabilizing the generated fluoroalkyl radicals (**4s**). Next, we examined the applicability of bromodifluoroacetamide derivatives bearing reactive N–H functionalities. A variety of such substrates, including those bearing aryl, alkyl, heterocyclic, and even primary amide groups, were found to be compatible with the catalytic system, affording the desired products in good yields (**4t**–**4x**). Notably, 2-iodo-1,1,1-trifluoropropane and bromoacetonitrile, bearing an additional methylene unit, also underwent smooth transformation under the standard conditions, furnishing the corresponding allenyl primary amide products **4y** and **4z** in 43% and 56% yield, respectively.

**Scheme 3:**
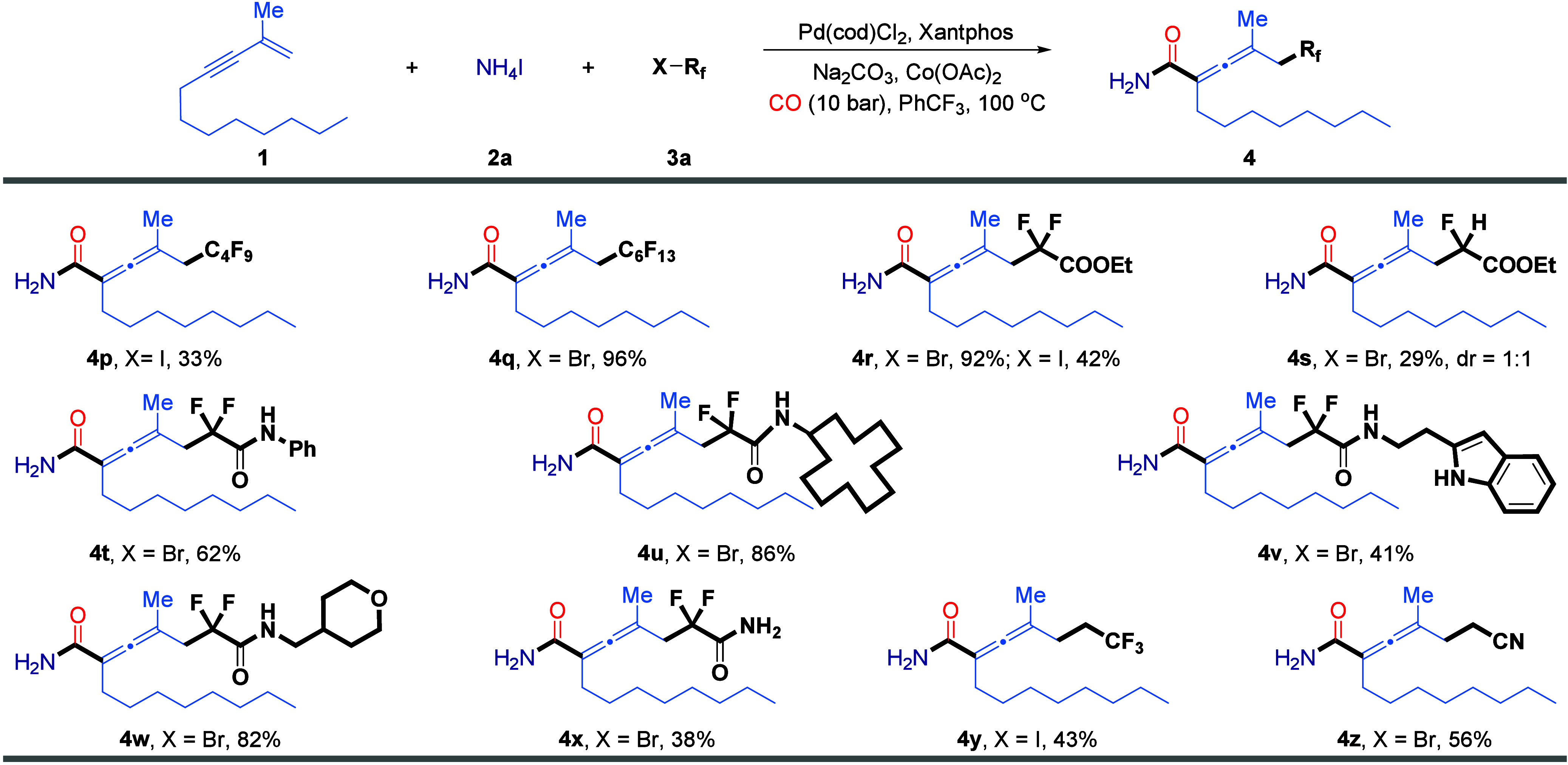
Scope of Fluoroalkyl Halides^a^ ^,^ ^b^

a Reaction conditions: **1a** (0.1 mmol), **2a** (0.15 mmol), **3a** (0.3 mmol), Pd­(cod)­Cl_2_ (5 mol %), Xantphos (5 mol %), Na_2_CO_3_ (0.3 mmol), Co­(OAc)_2_ (50 mol %), CO (10 bar), PhCF_3_ (1.0 mL), 100 °C, 18 h.
b Isolated yields are reported.

To further highlight the synthetic utility and diversification potential of this strategy, a series of representative downstream transformations of the highly reactive allenyl primary amide intermediate were conducted, as depicted in Scheme 4A. The multifunctional nature of this scaffold enables straightforward access to structurally complex and value-added heterocycles under mild conditions. For example, compound **4a** underwent efficient intramolecular cyclization in the presence of stoichiometric amounts of CuCl_2_ and CuBr_2_ in aqueous tetrahydrofuran, furnishing the corresponding halogenated pyrrol-2-ones **5a** and **5b** in good yields. Similarly, treatment with *N*-iodosuccinimide (NIS) provided the corresponding iodo analogue **5c** in 62% yield. These alkenyl halides, bearing synthetically versatile C–X bonds, serve as ideal precursors for further derivatization through classical cross-coupling reactions, expanding the molecular complexity in a modular fashion. Notably, under palladium catalysis, the allenyl primary amide underwent direct coupling with iodobenzene to furnish the iminolactone product **5d**, further highlighting the broad applicability and modular nature of this transformation platform.

**Scheme 4:**
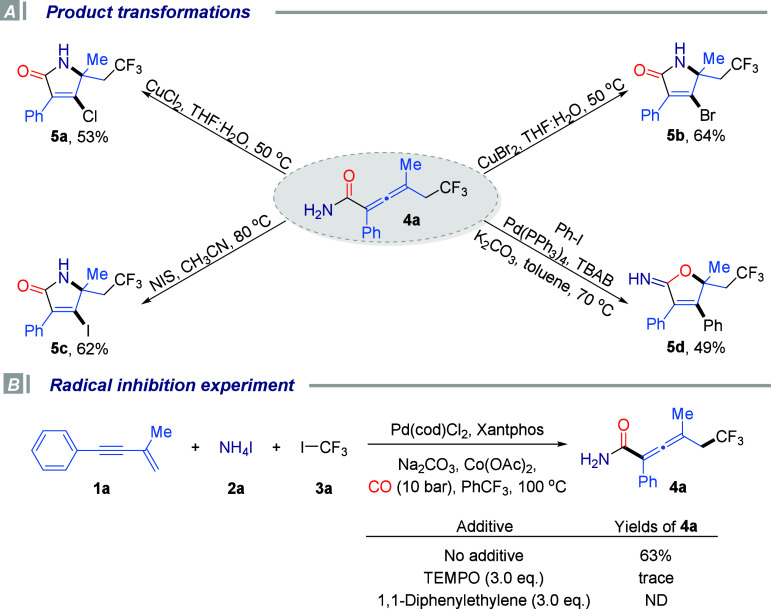
Product Transformations and Mechanistic Studies

To gain insight into the reaction mechanism of this difunctional fluoroalkylative carbonylation for the synthesis of allenyl primary amides, radical inhibition studies were conducted (Scheme 4B). When the standard reaction was carried out in the presence of the radical scavenger TEMPO, only trace amounts of the desired product were detected, indicating that the reaction was significantly inhibited. Moreover, the addition of 1,1-diphenylethylene completely inhibited product formation. These observations strongly suggest that the transformation proceeds through a radical-mediated pathway.

Based on mechanistic studies and relevant literature precedents,^12^ a plausible catalytic cycle is proposed as depicted in Scheme 5. At the initial stage, the Pd^II^ precatalyst is reduced *in situ* to the active Pd^0^L_*n*_ species **A** under ligand assistance. Subsequently, the Pd^0^L_*n*_ complex **A** undergoes single-electron transfer (SET) with the fluoroalkyl halide (R_f_–X, **3**), generating fluoroalkyl radical **B** and Pd^I^L_*n*_X species **E**. The resulting fluoroalkyl radical **B** rapidly adds to the 1,3-enyne (**1**), forming propargyl radical intermediate **C**, which then undergoes fast radical isomerization to afford the more stable allenyl radical **D**. The Pd^I^L_*n*_X species **E** then traps allenyl radical **D** to form Pd^II^–allenyl complex **F**, which undergoes carbon monoxide insertion to yield acylpalladium intermediate **G**. Under basic conditions, ammonium iodide (**2a**) might react with Co­(OAc)_2_ to give a Co­(NH_3_)_6_^*x*+^ complex (*x* = 2 or 3) with various anions, which then attacks intermediate **G**, followed by reductive elimination to afford the allenyl primary amide product **4a**. Concurrently, the Pd^0^L_*n*_ catalytic species is regenerated, thus completing the catalytic cycle.

**Scheme 5:**
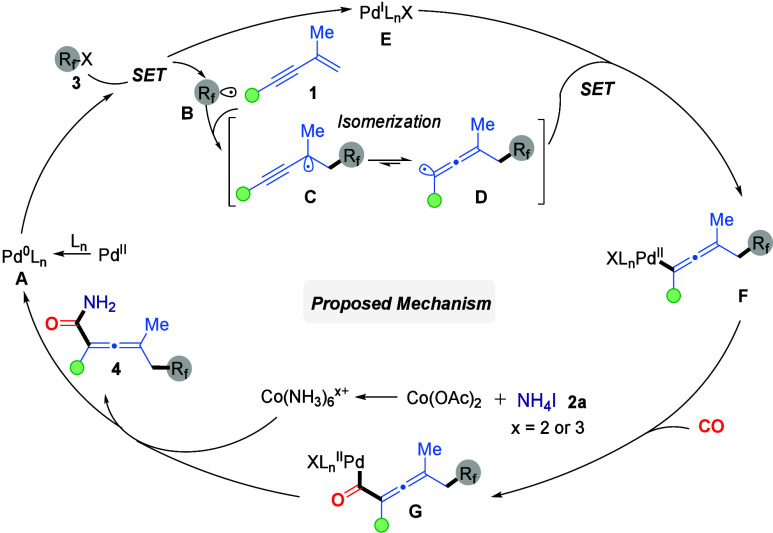
Proposed Mechanism

In summary, we have developed a palladium-catalyzed difunctional fluoroalkylative carbonylation of 1,3-enynes that enables the efficient, modular, and one-step synthesis of fluoroalkyl-substituted allenyl primary amides from readily accessible starting materials. This transformation features a broad substrate scope, excellent functional group tolerance, and high chemo- and regioselectivity under mild conditions. The resulting fluoroalkylated allenyl primary amides are valuable scaffolds with great potential for drug discovery and functional material development.

## Supplementary Material



## Data Availability

The data underlying this study are available in the published article and its Supporting Information.

## Notes

Experimental details, characterization data, and NMR spectra (PDF)
