# Supplementary material for: Palladium-Catalyzed Carbonylative Fluoroalkylation of 1,3-Enynes to Access Allenyl Primary Amides
Source: Org Lett. 2025 Aug 20;27(35):9807–13. doi: 10.1021/acs.orglett.5c03213 (PMC12418493; doi:10.1021/acs.orglett.5c03213)

Supporting Information

Palladium-Catalyzed Carbonylative Fluoroalkylation of 1,3-Enynes to Access Allenyl Primary Amides

Chang-Sheng Kuai[a], [b], Ru-Han A[a,c], Zhi-Peng Bao[a,c], and Xiao-Feng Wu[a], [b], [c]*

[a] Dalian National Laboratory for Clean Energy, Dalian Institute of Chemical Physics, Chinese Academy of Sciences, Dalian 116023 China

[b] University of Chinese Academy of Sciences, Beijing 100049, China

[c] Leibniz-Institut für Katalyse e. V., Albert-Einstein-Straβe 29a, 18059 Rostock, Germany

Supporting Information Placeholder

Table of Contents

1. General experimental details. 2

2. Typical procedure for Pd-catalyzed difunctional fluoroalkylative carbonylation of 1,3-enynes to allenyl primary amides...3

3. Product transformations. 4

4. Radical inhibition experiment. 5

5. Spectroscopic Data of Products. 6

6. NMR Spectra of the Products. 16

1. General experimental details.

Unless otherwise noted, all reactions were carried out under a carbon monoxide or nitrogen atmosphere. The amines, enynes and reagents were ordered from Adamas-beta®, Energy Chemical Sigma-Aldrich, Bidepharm and used without purification. All solvents were dried by standard techniques and distilled prior to use. Column chromatography was performed on silica gel (200-300 meshes). All NMR spectra were recorded at ambient temperature using Bruker Avance III 400 MHz NMR (^{1}H, 400 MHz; ^{13}C {^{1}H}, 101 MHz, ^{19}F 376 MHz), Bruker AVANCE III HD 700MHz NMR spectrometers (^{1}H, 700 MHz; ^{13}C{^{1}H}, 100 MHz). ^{1}H NMR chemical shifts are reported relative to TMS and were referenced via residual proton resonances of the corresponding deuterated solvent ($CDCl_3$: 7.26 ppm) whereas ^{13}C{^{1}H} NMR spectra are reported relative to TMS via the carbon signals of the deuterated solvent ($CDCl_3$: 77.0 ppm). Data for ^{1}H are reported as follows: chemical shift (δ ppm), multiplicity (s = singlet, d = doublet, t = triplet, q = quartet, quint = quintet, m = multiplet, br = broad), coupling constant (Hz), and integration. All ^{13}C NMR spectra were broad-band ^{1}H decoupled. All reactions were monitored by GC-FID or NMR analysis. HRMS data was obtained with Micromass HPLC-Q-TOF mass spectrometer (ESI) or Agilent 6540 Accurate-MS spectrometer (Q-TOF).

Because of the high toxicity of carbon monoxide, all the reactions should be performed in an autoclave. The laboratory should be well-equipped with a CO detector and alarm system.

2. Typical procedure for Pd-catalyzed difunctional fluoroalkylative carbonylation of 1,3-enynes to allenyl primary amides.

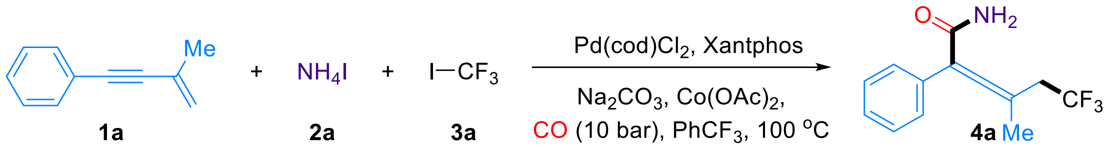

A 4 mL screw-cap vial was charged with Pd(cod)Cl_2 (5 mol%, 1.425 mg), Xantphos (5 mol%, 2.89 mg), $Co(OAc)_2$ (50 mol%, 8.85 mg), Na_2CO_3 (0.3 mmol), and an oven-dried stir bar. The vial was closed with a Teflon septum and cap and connected to the atmosphere via a needle. After $PhCF_3$ (1.0 mL), 1,3-enyne (**1a**, 0.10 mmol), NH_4I (**2a**, 0.15 mmol) and iodotrifluoromethane (**3a**, 0.30 mmol), were added with a syringe under argon atmosphere, the vial was moved to an alloy plate and put into a Parr 4560 series autoclave (300 mL) under an argon atmosphere. At room temperature, the autoclave was flushed with CO three times and charged with 10 bar of CO. The autoclave was placed on a heating plate equipped with a magnetic stirrer and an aluminum block. The reaction mixture was heated to 100 ^{o}C for 18h. After the reaction was complete, the autoclave was cooled down with ice water to room temperature and the pressure was released carefully. The reaction mixture concentration under reduced pressure, the crude product was purified by column chromatography on silica gel to afford the corresponding product **4a**.

1 mmol scale reaction: A 12 mL screw-cap vial was charged with Pd(cod)Cl_2 (5 mol%, 14.25 mg), Xantphos (5 mol%, 28.9 mg), $Co(OAc)_2$ (50 mol%, 88.5 mg), Na_2CO_3 (3 mmol, 318 mg), and an oven-dried stir bar. The vial was closed with a Teflon septum and cap and connected to the atmosphere via a needle. After $PhCF_3$ (5.0 mL), 1,3-enyne (**1a**, 1 mmol), NH_4I (**2a**, 1.5 mmol) and iodotrifluoromethane (**3a**, 3 mmol), were added with a syringe under argon atmosphere, the vial was moved to an alloy plate and put into a Parr 4560 series autoclave (300 mL) under an argon atmosphere. At room temperature, the autoclave was flushed with CO three times and charged with 10 bar of CO. The autoclave was placed on a heating plate equipped with a magnetic stirrer and an aluminum block. The reaction mixture was heated to 100 ^{o}C for 18h. After the reaction was complete, the autoclave was cooled down with ice water to room temperature and the pressure was released carefully. The reaction mixture concentration under reduced pressure, the crude product was purified by column chromatography on silica gel to afford the corresponding product **4a** in 60% yield (153 mg).

3. Product transformations.

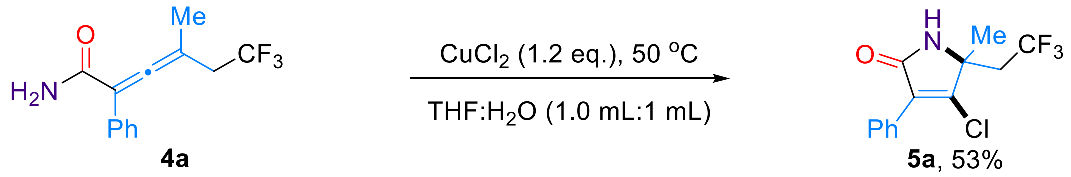

In a Schlenk tube (4.0 mL), allenyl primary amide **4a** (0.10 mmol), $CuCl_2$ (0.12 mmol) was dissolved in mixture of EtOH (1.0 mL) and H_2O (1.0 mL). Then the Schlenk tube was placed in an oil bath pre-heated at 50 °C with stirring for 16 h. After extraction with ethyl acetate (5 mL x 3), the organic layer was then washed with brine (10 mL) and dried over anhydrous Na_2SO_4. After filtration and concentration under reduced pressure, the crude product was purified by column chromatography on silica gel to afford **5a**.

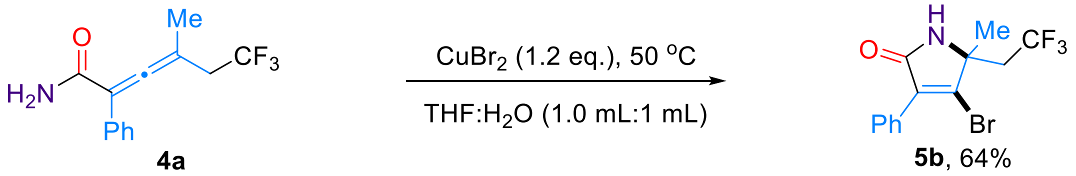

In a Schlenk tube (4.0 mL), allenyl primary amide **4a** (0.10 mmol), $CuBr_2$ (0.12 mmol) was dissolved in mixture of EtOH (1.0 mL) and H_2O (1.0 mL). Then the Schlenk tube was placed in an oil bath pre-heated at 50 °C with stirring for 16 h. After extraction with ethyl acetate (5 mL x 3), the organic layer was then washed with brine (10 mL) and dried over anhydrous Na_2SO_4. After filtration and concentration under reduced pressure, the crude product was purified by column chromatography on silica gel to afford **5b**.

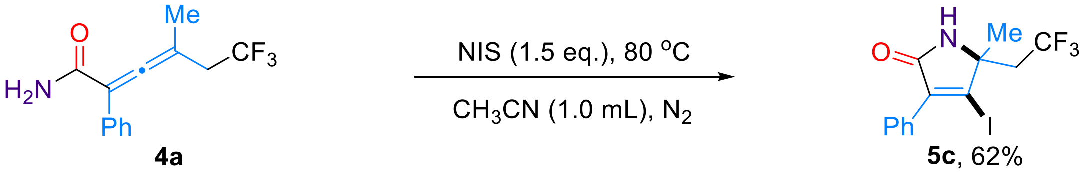

In a Schlenk tube (4.0 mL), allenyl primary amide **4a** (0.10 mmol), NIS (0.15 mmol) was dissolved in mixture of CH_3CN (1.0 mL). At room temperature, the Schlenk tube was flushed with N_2 three times. Then the Schlenk tube was placed in an oil bath pre-heated at 80 °C with stirring for 12 h. After filtration and concentration under reduced pressure, the crude product was purified by column chromatography on silica gel to afford **5c**.

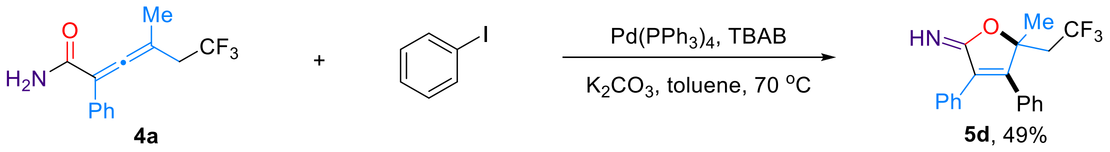

To a mixture of potassium carbonate (0.2 mmol), TBAB (5 mol%), and $Pd(PPh_3)_4$ (10 mol%) in toluene (1 mL) were added allenyl primary amide **4a** (0.1 mmol) and iodobenzene (0.11 mmol) sequentially under Ar. The resulting mixture was heated to 70 °C for 48 h. After evaporation, the residue was purified by flash chromatography on silica gel to afford 49% of **5d**:

4. Radical inhibition experiment.

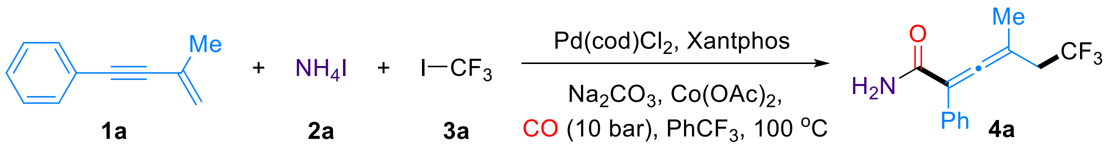

Additive	Yields of **4a**
No additive	63%
TEMPO (3.0 eq.)	trace
1,1-Diphenylethylene (3.0 eq.)	NR

A 4 mL screw-cap vial was charged with Pd(cod)Cl_2 (5 mol%), xantphos (5 mol%), Na_2CO_3 (0.3 mmol), $Co(OAc)_2$ (0.05 mmol), radical scavenger TEMPO or 1,1-Diphenylethylene, and an oven-dried stir bar. The vial was closed with a Teflon septum and cap and connected to the atmosphere via a needle. After $PhCF_3$ (1.0 mL), **1a** (0.10 mmol), NH_4I **2a** (0.15 mmol) and iodotrifluoromethane (**3a**, 0.20 mmol) were added with a syringe under argon atmosphere, the vial was moved to an alloy plate and put into a Parr 4560 series autoclave (300 mL) under an argon atmosphere. At room temperature, the autoclave was flushed with CO three times and charged with 10 bar of CO. The autoclave was placed on a heating plate equipped with a magnetic stirrer and an aluminum block. The reaction mixture was heated to 100 °C for 18h. After the reaction was complete, the autoclave was cooled down with ice water to room temperature and the pressure was released carefully. The reaction mixture concentration under reduced pressure, the crude product was purified by column chromatography on silica gel to afford the corresponding product **4a**.

5. Spectroscopic Data of Products.

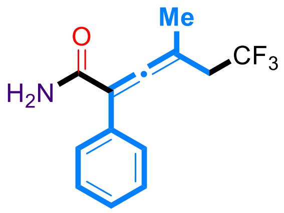

6,6,6-Trifluoro-4-methyl-2-phenylhexa-2,3-dienamide (4a):

Brown oil, 16.1mg, 63% yield, R_f=0.2 (PE/EtOAc 3/1).

^{1}H NMR (400 MHz, $CDCl_3$) δ 7.47 – 7.39 (m, 2H), 7.37 – 7.31 (m, 2H), 7.30 – 7.25 (m, 1H), 6.60 (s, 1H), 5.99 (s, 1H), 3.05 – 2.83 (m, 2H), 1.99 (s, 3H).

^{13}C NMR (100 MHz, $CDCl_3$) δ 206.9, 168.0, 132.4, 128.7, 128.4, 128.0, 125.8 (q, J = 277.7 Hz), 105.1, 96.6 (q, J = 3.1 Hz), 38.3 (q, J = 29.6 Hz), 18.9.

^{19}F NMR (376 MHz, $CDCl_3$) δ -64.41.

HRMS (ESI-TOF) m/z: $[M + H]^+$ calculated for $C_{13}H_{13}F_3NO$ 256.0944; Found 256.0944.

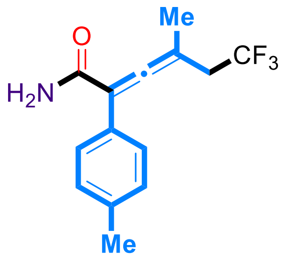

6,6,6-Trifluoro-4-methyl-2-(p-tolyl)hexa-2,3-dienamide (4b):

Yellow oil, 17.4 mg, 65% yield, R_f=0.2 (PE/EtOAc 3/1).

^{1}H NMR (700 MHz, $CDCl_3$) δ 7.32 (d, J = 8.0 Hz, 2H), 7.16 (d, J = 7.9 Hz, 2H), 5.90 (d, J = 37.6 Hz, 2H), 3.01 (dq, J = 15.0, 10.6 Hz, 1H), 2.91 (dq, J = 15.1, 10.4 Hz, 1H), 2.34 (s, 3H), 2.01 (s, 3H).

^{13}C NMR (176 MHz, $CDCl_3$) δ 206.8, 167.6, 138.0, 129.3, 129.2, 128.6, 125.8 (q, J = 277.7 Hz), 104.9, 96.3 (q, J = 2.9 Hz), 38.4 (q, J = 29.6 Hz), 21.2, 19.0.

^{19}F NMR (376 MHz, $CDCl_3$) δ -64.43.

HRMS (ESI-TOF) m/z: $[M + H]^+$ calculated for $C_{14}H_{15}F_3NO$ 270.1100; Found 270.1101.

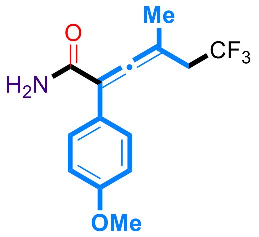

6,6,6-Trifluoro-2-(4-methoxyphenyl)-4-methylhexa-2,3-dienamide (4c):

Brown oil, 18.1mg, 64% yield, R_f=0.2 (PE/EtOAc 1/1).

^{1}H NMR (700 MHz, $CDCl_3$) δ 7.38 (d, J = 8.8 Hz, 2H), 6.89 (d, J = 8.8 Hz, 2H), 5.91 (d, J = 54.9 Hz, 2H), 3.81 (s, 3H), 3.01 (dq, J = 15.0, 10.7 Hz, 1H), 2.91 (dq, J = 15.1, 10.4 Hz, 1H), 2.01 (s, 3H).

^{13}C NMR (176 MHz, $CDCl_3$) δ 206.7, 167.7, 159.5, 129.9, 125.8 (q, J = 277.7 Hz), 124.4, 113.9, 104.6, 96.3 (q, J = 2.9 Hz), 55.3, 38.5 (q, J = 29.5 Hz), 19.0.

^{19}F NMR (376 MHz, $CDCl_3$) δ -64.43.

HRMS (ESI-TOF) m/z: $[M + H]^+$ calculated for $C_{14}H_{15}F_3NO_2$ 286.1049; Found 286.1048.

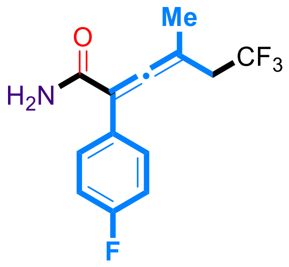

6,6,6-Trifluoro-2-(4-fluorophenyl)-4-methylhexa-2,3-dienamide (4d):

Brown oil, 18.7mg, 68% yield, R_f=0.2 (PE/EtOAc 2/1).

^{1}H NMR (700 MHz, $CDCl_3$) δ 7.43 (dd, J = 8.4, 5.5 Hz, 2H), 7.04 (t, J = 8.6 Hz, 2H), 5.94 (d, J = 17.5 Hz, 2H), 3.03 (dq, J = 15.1, 10.6 Hz, 1H), 2.92 (dq, J = 15.2, 10.4 Hz, 1H), 2.03 (s, 3H).

^{13}C NMR (176 MHz, $CDCl_3$) δ 206.7, 167.3, 162.5 (d, J = 247.8 Hz), 130.5 (d, J = 8.1 Hz), 128.1 (d, J = 3.4 Hz), 125.7 (q, J = 277.6 Hz), 115.4 (d, J = 21.7 Hz), 104.1, 96.9 (q, J = 3.0 Hz), 38.4 (q, J = 29.6 Hz), 19.0.

^{19}F NMR (376 MHz, $CDCl_3$) δ -64.45, -113.81.

HRMS (ESI-TOF) m/z: $[M + H]^+$ calculated for $C_{13}H_{12}F_4NO$ 274.0850; Found 274.0845.

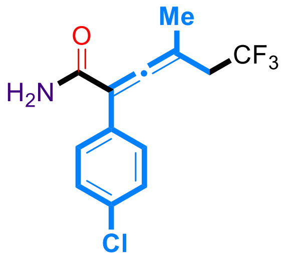

2-(4-Chlorophenyl)-6,6,6-trifluoro-4-methylhexa-2,3-dienamide (4e):

Brown oil, 15.8 mg, 55% yield, R_f=0.2 (PE/EtOAc 3/1).

^{1}H NMR (700 MHz, $CDCl_3$) δ 7.39 (d, J = 8.3 Hz, 2H), 7.32 (d, J = 8.3 Hz, 2H), 5.92 (d, J = 38.9 Hz, 2H), 3.07 – 2.99 (m, 1H), 2.97 – 2.88 (m, 1H), 2.03 (s, 3H).

^{13}C NMR (176 MHz, $CDCl_3$) δ 206.8, 167.0, 134.0, 130.7, 130.0, 128.6, 125.6 (q, J = 277.6 Hz), 104.1, 97.1 (q, J = 3.0 Hz), 38.4 (q, J = 29.7 Hz), 19.0.

^{19}F NMR (376 MHz, $CDCl_3$) δ -64.44.

HRMS (ESI-TOF) m/z: $[M + H]^+$ calculated for $C_{13}H_{12}ClF_3NO$ 290.0554; Found 290.0551.

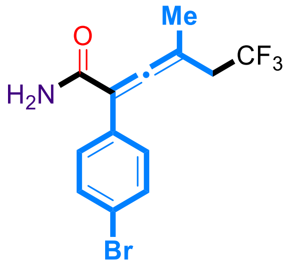

2-(4-Bromophenyl)-6,6,6-trifluoro-4-methylhexa-2,3-dienamide (4f):

Brown solid, 9.5mg, 29% yield, R_f=0.2 (PE/EtOAc 2/1).

^{1}H NMR (400 MHz, $CDCl_3$) δ 7.47 (d, J = 8.2 Hz, 2H), 7.33 (d, J = 8.2 Hz, 2H), 5.94 (s, 1H), 5.78 (s, 1H), 3.09 – 2.98 (dq, J = 15.1, 10.3 Hz, 1H), 2.92 (dq, J = 15.1, 10.3 Hz, 1H), 2.03 (s, 3H).

^{13}C NMR (100 MHz, $CDCl_3$) δ 206.7, 166.9, 131.5, 131.2, 130.3, 125.6 (q, J = 277.6 Hz), 122.2, 104.1, 97.2 (q, J = 3.0 Hz), 38.3 (q, J = 29.5 Hz), 18.9.

^{19}F NMR (376 MHz, $CDCl_3$) δ -64.43.

HRMS (ESI-TOF) m/z: $[M + H]^+$ calculated for $C_{13}H_{12}BrF_3NO$ 334.0049; Found 334.0058.

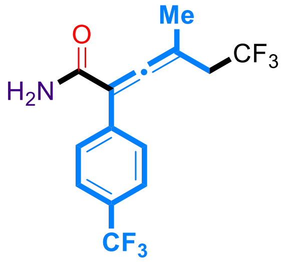

6,6,6-Trifluoro-4-methyl-2-(4-(trifluoromethyl)phenyl)hexa-2,3-dienamide (4g):

Yellow solid, 16.7 mg, 52% yield, R_f=0.2 (PE/EtOAc 3/1).

^{1}H NMR (700 MHz, $CDCl_3$) δ 7.59 (q, J = 8.3 Hz, 4H), 5.97 (s, 1H), 5.86 (s, 1H), 3.05 (dq, J = 15.0, 10.5 Hz, 1H), 2.95 (dq, J = 15.3, 10.3 Hz, 1H), 2.05 (s, 3H).

^{13}C NMR (176 MHz, $CDCl_3$) δ 207.1, 166.7, 136.0, 130.0 (q, J = 32.5 Hz), 129.1, 125.6 (q, J = 267.7 Hz), 125.3 (q, J = 3.7 Hz), 124.0 (q, J = 262.1 Hz), 104.1, 97.6 (q, J = 3.0 Hz),, 38.3 (q, J = 29.8 Hz), 18.9.

^{19}F NMR (376 MHz, $CDCl_3$) δ -62.70, 64.44.

HRMS (ESI-TOF) m/z: $[M + H]^+$ calculated for $C_{14}H_{12}F_6NO$ 324.0818; Found 324.0819.

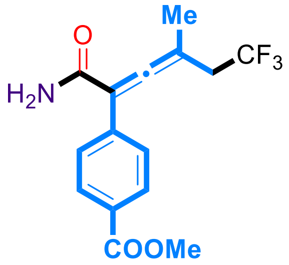

Methyl 4-(1-amino-6,6,6-trifluoro-4-methyl-1-oxohexa-2,3-dien-2-yl)benzoate (4h):

Yellow solid, 14.1mg, 45% yield, R_f=0.2 (PE/EtOAc 3/1).

^{1}H NMR (700 MHz, $CDCl_3$) δ 8.01 (d, J = 8.1 Hz, 2H), 7.54 (d, J = 8.1 Hz, 2H), 5.97 (s, 1H), 5.82 (s, 1H), 3.92 (s, 3H), 3.11 – 3.00 (m, 1H), 2.99 – 2.89 (m, 1H), 2.05 (s, 3H).

^{13}C NMR (176 MHz, $CDCl_3$) δ 207.2, 166.78, 166.75, 137.0, 129.6, 129.5, 128.6, 125.6 (q, J = 277.7 Hz), 104.5, 97.4 (q, J = 3.0 Hz), 52.2, 38.3 (q, J = 29.7 Hz), 18.9.

^{19}F NMR (376 MHz, $CDCl_3$) δ -64.43.

HRMS (ESI-TOF) m/z: $[M + H]^+$ calculated for $C_{15}H_{15}F_3NO_3$ 314.0999; Found 314.1002.

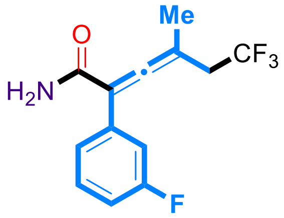

6,6,6-Trifluoro-2-(3-fluorophenyl)-4-methylhexa-2,3-dienamide (4j):

Brown oil, 14.9 mg, 55% yield, R_f=0.2 (PE/EtOAc 3/1).

^{1}H NMR (700 MHz, $CDCl_3$) δ 7.31 (q, J = 7.8 Hz, 1H), 7.24 (d, J = 7.8 Hz, 1H), 7.20 (d, J = 10.1 Hz, 1H), 7.01 – 6.97 (m, 1H), 5.93 (d, J = 22.2 Hz, 2H), 3.04 (dq, J = 15.0, 10.6 Hz, 1H), 2.93 (dq, J = 15.2, 10.4 Hz, 1H), 2.04 (s, 3H).

^{13}C NMR (176 MHz, $CDCl_3$) δ 206.8, 166.9, 162.7 (d, J = 245.5 Hz), 134.4 (d, J = 8.1 Hz), 129.8 (d, J = 8.3 Hz), 125.6 (q, J = 277.7 Hz), 124.4 (d, J = 2.8 Hz), 115.7 (d, J = 23.1 Hz), 114.9 (d, J = 21.3 Hz), 104.2, 97.3 (q, J = 3.0 Hz), 38.3 (q, J = 29.7 Hz), 18.9.

^{19}F NMR (376 MHz, $CDCl_3$) δ -64.44, 113.02.

HRMS (ESI-TOF) m/z: $[M + H]^+$ calculated for $C_{13}H_{12}F_4NO$ 274.0850; Found 274.0849.

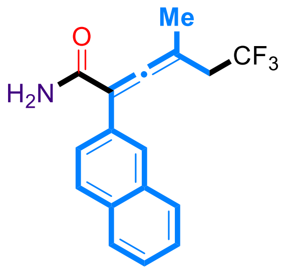

6,6,6-Trifluoro-4-methyl-2-(naphthalen-2-yl)hexa-2,3-dienamide (4k):

Brown oil, 24.4 mg, 80% yield, R_f=0.2 (PE/EtOAc 3/1).

^{1}H NMR (700 MHz, $CDCl_3$) δ 8.01 (s, 1H), 7.82 (dd, J = 16.0, 7.1 Hz, 3H), 7.53 – 7.43 (m, 3H), 6.01 (s, 1H), 5.89 (s, 1H), 3.06 (dq, J = 14.9, 10.6 Hz, 1H), 2.96 (dq, J = 15.1, 10.4 Hz, 1H), 2.07 (s, 3H).

^{13}C NMR (176 MHz, $CDCl_3$) δ 207.2, 167.5, 133.3, 132.9, 129.5, 128.3, 128.0, 128.0, 127.6, 126.4, 126.3, 126.2, 125.8 (d, J = 277.7 Hz), 105.2, 96.8 (q, J = 3.1 Hz), 38.5 (q, J = 29.6 Hz), 19.0.

^{19}F NMR (376 MHz, $CDCl_3$) δ -64.36.

HRMS (ESI-TOF) m/z: $[M + H]^+$ calculated for $C_{17}H_{15}F_3NO$ 306.1100; Found 306.1105.

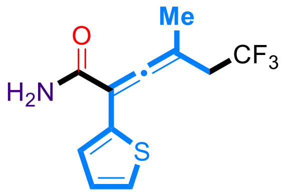

6,6,6-Trifluoro-4-methyl-2-(thiophen-2-yl)hexa-2,3-dienamide (4l):

Brown oil, 11.3 mg, 43% yield, R_f=0.2 (PE/EtOAc 2/1).

^{1}H NMR (700 MHz, $CDCl_3$) δ 7.32 (d, J = 5.0 Hz, 1H), 7.29 (d, J = 3.4 Hz, 1H), 7.04 – 7.00 (m, 1H), 5.96 (s, 1H), 5.66 (s, 1H), 3.07 (dq, J = 15.0, 10.6 Hz, 1H), 2.95 (dq, J = 15.1, 10.3 Hz, 1H), 2.04 (s, 3H).

^{13}C NMR (176 MHz, $CDCl_3$) δ 206.1, 166.4, 133.2, 127.2, 127.0, 127.0, 125.6 (q, J = 277.7 Hz), 100.1, 98.3 (q, J = 3.1 Hz), 38.4 (q, J = 29.7 Hz), 19.0.

^{19}F NMR (376 MHz, $CDCl_3$) δ -64.43.

HRMS (ESI-TOF) m/z: $[M + H]^+$ calculated for $C_{11}H_{11}F_3NOS$ 262.0508; Found 262.0507.

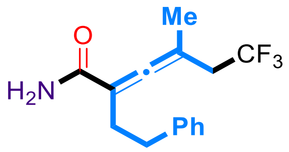

6,6,6-Trifluoro-4-methyl-2-phenethylhexa-2,3-dienamide (4m):

Brown oil, 26.0mg, 92% yield, R_f=0.2 (PE/EtOAc 3/1).

^{1}H NMR (700 MHz, $CDCl_3$) δ 7.28 – 7.24 (m, 2H), 7.17 (d, J = 7.6 Hz, 3H), 5.81 (s, 1H), 5.64 (s, 1H), 2.79 (qt, J = 14.1, 7.3 Hz, 2H), 2.66 (dt, J = 14.2, 7.0 Hz, 1H), 2.58 (ddd, J = 24.5, 15.2, 6.0 Hz, 2H), 2.54 – 2.46 (m, 1H), 1.72 (s, 3H).

^{13}C NMR (176 MHz, $CDCl_3$) δ 206.3, 168.1, 141.2, 128.6, 128.3, 125.9, 125.8 (q, J = 277.7 Hz), 101.2, 95.7 (q, J = 3.0 Hz), 38.1 (q, J = 29.4 Hz), 33.8, 29.2, 18.6.

^{19}F NMR (376 MHz, $CDCl_3$) δ -64.72.

HRMS (ESI-TOF) m/z: $[M + H]^+$ calculated for $C_{15}H_{17}F_3NO$ 284.1257; Found 284.1259.

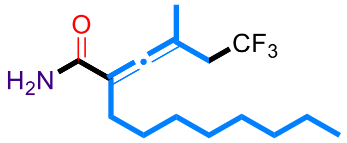

2-(4,4,4-Trifluoro-2-methylbut-1-en-1-ylidene)decanamide (4n):

Light yellow oil, 26.0 mg, 89% yield, R_f=0.2 (PE/EtOAc 3/1).

^{1}H NMR (700 MHz, $CDCl_3$) δ 5.83 (s, 1H), 5.55 (s, 1H), 2.92 (dq, J = 14.9, 10.7 Hz, 1H), 2.82 (dq, J = 15.0, 10.4 Hz, 1H), 2.25 (h, J = 8.2 Hz, 2H), 1.92 (s, 3H), 1.40 (dq, J = 12.7, 7.2 Hz, 2H), 1.32 – 1.24 (m, 10H), 0.88 (t, J = 7.1 Hz, 3H).

^{13}C NMR (176 MHz, $CDCl_3$) δ 205.7, 168.4, 125.8 (q, J = 277.6 Hz), 102.8, 95.4 (q, J = 3.0 Hz), 38.5 (q, J = 29.4 Hz), 31.9, 29.4, 29.3, 29.2, 27.8, 27.6, 22.7, 18.9, 14.1.

^{19}F NMR (376 MHz, $CDCl_3$) δ -64.65.

HRMS (ESI-TOF) m/z: $[M + H]^+$ calculated for $C_{15}H_{25}F_3NO$ 292.1883; Found 292.1886.

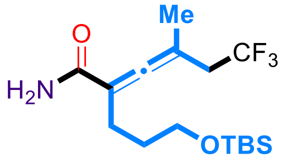

2-(3-((Tert-butyldimethylsilyl)oxy)propyl)-6,6,6-trifluoro-4-methylhexa-2,3-dienamide (4o):

Light yellow oil, 22.8 mg, 65% yield, R_f=0.2 (PE/EtOAc 3/1).

^{1}H NMR (700 MHz, $CDCl_3$) δ 5.80 (s, 1H), 5.40 (s, 1H), 3.59 (t, J = 6.5 Hz, 2H), 2.88 (dq, J = 14.9, 10.7 Hz, 1H), 2.77 (dq, J = 15.0, 10.4 Hz, 1H), 2.32 – 2.22 (m, 2H), 1.87 (s, 3H), 1.60 (dd, J = 12.9, 4.9 Hz, 2H), 0.84 (s, 9H), -0.00 (s, 6H).

^{13}C NMR (176 MHz, $CDCl_3$) δ 205.7, 168.1, 125.8 (q, J = 277.6 Hz), 102.5, 95.7 (q, J = 2.9 Hz), 62.5, 38.5 (d, J = 29.4 Hz), 31.1, 25.9, 24.2, 19.0, 18.4, -5.3.

^{19}F NMR (376 MHz, $CDCl_3$) δ -64.64.

HRMS (ESI-TOF) m/z: $[M + H]^+$ calculated for $C_{16}H_{29}F_3NO_2Si$ 352.1914; Found 352.1917.

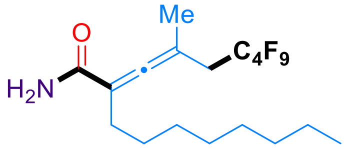

2-(4,4,5,5,6,6,7,7,7-Nonafluoro-2-methylhept-1-en-1-ylidene)decanamide (4p):

Brown oil, 14.4 mg, 33% yield, R_f=0.2 (PE/EtOAc 3/1).

^{1}H NMR (700 MHz, $CDCl_3$) δ 5.85 (s, 1H), 5.33 (s, 1H), 2.91 (ddd, J = 27.9, 14.9, 10.7 Hz, 1H), 2.81 (ddd, J = 25.3, 14.9, 10.2 Hz, 1H), 2.30 – 2.22 (m, 2H), 1.95 (s, 3H), 1.43 – 1.38 (m, 2H), 1.28 (ddd, J = 17.2, 13.0, 6.3 Hz, 10H), 0.88 (t, J = 7.1 Hz, 3H).

^{13}C NMR (176 MHz, $CDCl_3$) δ 206.3, 168.1, 102.6, 94.5, 35.5 (t, J = 21.9 Hz), 31.9, 29.4, 29.3, 29.2, 27.8, 27.7, 22.7, 19.7, 14.1.

^{19}F NMR (376 MHz, $CDCl_3$) δ -80.99 (tt, J = 9.6, 2.9 Hz), -112.68 – -112.95 (m), -124.05 – -124.20 (m), -125.80 – -125.94 (m)..

HRMS (ESI-TOF) m/z: $[M + H]^+$ calculated for $C_{18}H_{25}F_9NO$ 442.1787; Found 442.1790.

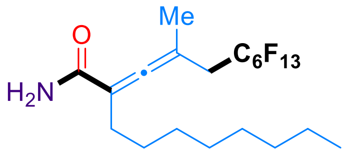

6,6,7,7,8,8,9,9,10,10,11,11,11-Tridecafluoro-4-methyl-2-octylundeca-2,3-dienamide (4q):

Brown oil, 52.2 mg, 96% yield, R_f=0.2 (PE/EtOAc 3/1).

^{1}H NMR (700 MHz, $CDCl_3$) δ 5.87 (s, 1H), 5.73 (s, 1H), 2.96 – 2.77 (m, 2H), 2.31 – 2.21 (m, 2H), 1.95 (s, 3H), 1.44 – 1.38 (m, 2H), 1.29 (ddd, J = 23.0, 13.0, 5.2 Hz, 10H), 0.87 (t, J = 7.1 Hz, 3H).

^{13}C NMR (176 MHz, $CDCl_3$) δ 206.3, 168.4, 117.9 (t, J = 35.7 Hz), 102.6, 94.5, 35.6 (t, J = 21.8 Hz), 31.8, 29.4, 29.3, 29.2, 27.8, 27.6, 22.6, 19.6, 14.0.

^{19}F NMR (376 MHz, $CDCl_3$) δ -80.85 (t, J = 9.9 Hz), -112.39 – -112.85 (m), -121.80, -122.89, -123.20 (d, J = 13.3 Hz), -126.00 – -126.34 (m).

HRMS (ESI-TOF) m/z: $[M + H]^+$ calculated for $C_{20}H_{25}F_{13}NO$ 542.1723; Found 542.1722.

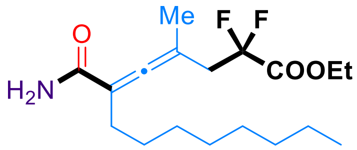

Ethyl-6-carbamoyl-2,2-difluoro-4-methyltetradeca-4,5-dienoate (4r):

Brown oil, 31.9 mg, 92% yield, R_f=0.2 (PE/EtOAc 3/1).

^{1}H NMR (700 MHz, $CDCl_3$) δ 6.04 (s, 1H), 5.43 (s, 1H), 4.33 (q, J = 7.1 Hz, 2H), 2.97 – 2.90 (m, 1H), 2.79 (q, J = 15.6 Hz, 1H), 2.28 – 2.24 (m, 1H), 2.20 (ddd, J = 15.1, 8.2, 6.7 Hz, 1H), 1.88 (s, 3H), 1.37 (dt, J = 14.3, 6.8 Hz, 5H), 1.28 (dt, J = 16.2, 8.4 Hz, 10H), 0.88 (t, J = 7.1 Hz, 3H).

^{13}C NMR (176 MHz, $CDCl_3$) δ 205.4, 168.6, 163.8 (t, J = 32.3 Hz), 115.1 (t, J = 252.4 Hz), 102.6, 96.0, 63.4, 38.9 (t, J = 23.7 Hz), 31.9, 29.4, 29.3, 29.2, 27.9, 27.7, 22.7, 19.6, 14.1, 13.9.

^{19}F NMR (376 MHz, $CDCl_3$) δ -103.78, -103.85.

HRMS (ESI-TOF) m/z: $[M + H]^+$ calculated for $C_{18}H_{30}F_2NO_3$ 346.2188; Found 346.2190.

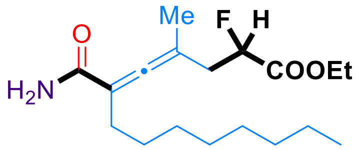

Ethyl-6-carbamoyl-2-fluoro-4-methyltetradeca-4,5-dienoate (4s):

Brown oil, 9.4 mg, 29% yield, R_f=0.2 (PE/EtOAc 3/1).

^{1}H NMR (700 MHz, $CDCl_3$) δ 6.15 (d, J = 19.1 Hz, 2H), 5.29 (s, 2H), 5.09 (dt, J = 8.6, 4.4 Hz, 1H), 5.02 (dt, J = 8.7, 4.6 Hz, 1H), 4.28 (ddt, J = 10.3, 7.3, 3.2 Hz, 4H), 2.86 – 2.68 (m, 2H), 2.68 – 2.50 (m, 2H), 2.29 – 2.18 (m, 4H), 1.87 (s, 3H), 1.84 (s, 3H), 1.39 (dq, J = 12.6, 6.9, 6.3 Hz, 4H), 1.34 – 1.31 (m, 8H), 1.30 – 1.24 (m, 18H), 0.88 (t, J = 7.1 Hz, 6H).

^{13}C NMR (176 MHz, $CDCl_3$) δ 204.3, 203.8, 169.3 (d, J = 13.4 Hz), 169.2 (d, J = 13.3 Hz), 169.0, 168.9, 130.5, 127.9, 103.4, 102.6, 99.0 (d, J = 2.2 Hz), 99.0 (d, J = 2.0 Hz), 87.6, 87.6, 86.6, 86.6, 62.1, 62.0, 37.1, 37.0, 36.5, 36.4, 31.9, 29.5 29.3, 29.3, 29.3, 29.2, 28.1, 28.0, 27.8, 27.7, 22.7, 19.2, 18.4, 14.1.

^{19}F NMR (376 MHz, $CDCl_3$) δ -189.28, -189.33.

HRMS (ESI-TOF) m/z: $[M + H]^+$ calculated for $C_{18}H_{31}FNO_3$ 328.2282; Found 328.2280.

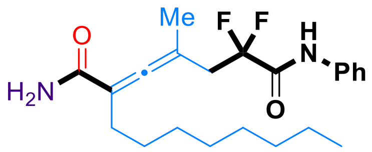

6,6-Difluoro-4-methyl-2-octyl-N7-phenylhepta-2,3-dienediamide (4t):

Brown solid, 24.4 mg, 62% yield, Rf=0.2 (PE/EtOAc 3/1).

^{1}H NMR (700 MHz, $CDCl_3$) δ 8.28 (s, 1H), 7.56 (d, J = 8.0 Hz, 2H), 7.36 (t, J = 7.8 Hz, 2H), 7.20 (t, J = 7.4 Hz, 1H), 6.17 (s, 1H), 5.31 (s, 1H), 3.05 (q, J = 16.2 Hz, 1H), 2.91 (q, J = 15.7 Hz, 1H), 2.22 (dt, J = 15.3, 7.7 Hz, 1H), 2.14 (dt, J = 15.1, 7.2 Hz, 1H), 1.89 (s, 3H), 1.37 (t, J = 9.3 Hz, 2H), 1.30 – 1.22 (m, 10H), 0.87 (t, J = 7.1 Hz, 3H).

^{13}C NMR (176 MHz, $CDCl_3$) δ 205.3, 168.8, 161.5 (t, J = 28.4 Hz), 135.9, 129.3, 125.9, 120.3, 117.0 (t, J = 255.6 Hz), 102.8, 96.4, 38.3 (t, J = 23.8 Hz), 31.9, 29.4, 29.3, 29.2, 27.9, 27.8, 22.7, 19.6, 14.1.

^{19}F NMR (376 MHz, $CDCl_3$) δ -102.48, -102.50.

HRMS (ESI-TOF) m/z: $[M + H]^+$ calculated for $C_{22}H_{31}F_2N_2O_2$ 393.2348; Found 393.2346.

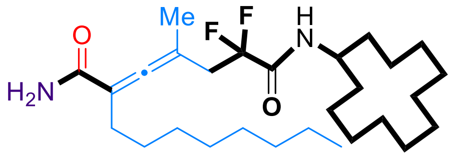

N^7-cyclododecyl-6,6-difluoro-4-methyl-2-octylhepta-2,3-dienediamide (4u):

White solid, 41.4 mg, 86% yield, R_f=0.2 (PE/EtOAc 3/1).

^{1}H NMR (700 MHz, $CDCl_3$) δ 6.49 (s, 1H), 6.26 (s, 1H), 5.36 (s, 1H), 4.04 (s, 1H), 2.92 (t, J = 16.7 Hz, 1H), 2.84 (t, J = 15.0 Hz, 1H), 2.22 (ddt, J = 44.2, 14.4, 7.3 Hz, 2H), 1.85 (s, 3H), 1.67 (s, 2H), 1.40 – 1.24 (m, 32H), 0.88 (t, J = 6.3 Hz, 3H).

^{13}C NMR (176 MHz, $CDCl_3$) δ 205.5, 169.0, 163.0 (t, J = 28.2 Hz), 117.0 (t, J = 254.4 Hz), 102.3, 96.3, 46.7, 38.6 (t, J = 23.9 Hz), 31.9, 29.9, 29.5, 29.3, 29.3, 27.9, 27.8, 23.8, 23.7, 23.6, 23.4, 23.4, 23.3, 23.23, 22.7, 21.3, 21.3, 19.3, 14.1.

^{19}F NMR (376 MHz, $CDCl_3$) δ -102.15, -102.25.

HRMS (ESI-TOF) m/z: $[M + H]^+$ calculated for $C_{28}H_{49}F_2N_2O_2$ 483.3757; Found 483.3759.

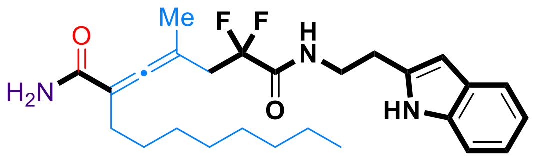

N^7-(2-(1H-indol-2-yl)ethyl)-6,6-difluoro-4-methyl-2-octylhepta-2,3-dienediamide (4v):

Brown oil, 18.9 mg, 41% yield, R_f=0.2 (PE/EtOAc 3/1).

^{1}H NMR (700 MHz, $CDCl_3$) δ 8.25 (s, 1H), 7.58 (d, J = 7.9 Hz, 1H), 7.38 (d, J = 8.1 Hz, 1H), 7.21 (t, J = 7.5 Hz, 1H), 7.13 (t, J = 7.5 Hz, 1H), 7.02 (s, 1H), 6.53 (s, 1H), 6.35 (s, 1H), 5.36 (s, 1H), 3.63 (dp, J = 13.6, 6.7 Hz, 2H), 3.01 (t, J = 6.7 Hz, 2H), 2.87 (q, J = 16.2 Hz, 1H), 2.77 (q, J = 15.4 Hz, 1H), 2.24 (dt, J = 15.2, 7.7 Hz, 1H), 2.15 (dt, J = 15.1, 7.2 Hz, 1H), 1.81 (s, 3H), 1.36 (dd, J = 13.7, 6.8 Hz, 2H), 1.26 (dd, J = 20.5, 7.3 Hz, 10H), 0.87 (t, J = 7.1 Hz, 3H).

^{13}C NMR (176 MHz, $CDCl_3$) δ 205.2, 169.1, 163.6 (t, J = 28.3 Hz), 136.5, 127.1, 122.4, 122.3, 119.7, 118.5, 116.9 (t, J = 254.4 Hz), 111.9, 111.4, 102.4, 96.4, 39.8, 38.5 (t, J = 24.0 Hz), 31.9, 29.5, 29.3, 29.3, 27.9, 27.8, 24.9, 22.7, 19.3, 14.1.

^{19}F NMR (376 MHz, $CDCl_3$) δ -102.61, -102.70.

HRMS (ESI-TOF) m/z: $[M + H]^+$ calculated for $C_{26}H_{36}F_2N_3O_2$ 460.2770; Found 460.2772.

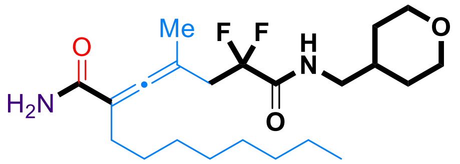

6,6-Difluoro-4-methyl-2-octyl-N7-((tetrahydro-2H-pyran-4-yl)methyl)hepta-2,3-dienediamide (4w):

Brown solid, 33.9 mg, 82% yield, R_f=0.2 (PE/EtOAc 3/1).

^{1}H NMR (700 MHz, $CDCl_3$) δ 6.64 (s, 1H), 6.31 (s, 1H), 5.44 (s, 1H), 3.99 – 3.96 (m, 2H), 3.37 (t, J = 11.6 Hz, 2H), 3.26 – 3.23 (m, 1H), 3.18 (dt, J = 13.3, 6.4 Hz, 1H), 2.94 (q, J = 16.3 Hz, 1H), 2.85 (q, J = 15.7 Hz, 1H), 2.27 – 2.22 (m, 1H), 2.20 – 2.14 (m, 1H), 1.86 (s, 3H), 1.80 (ddt, J = 11.5, 7.7, 4.1 Hz, 1H), 1.60 (d, J = 13.0 Hz, 2H), 1.38 (td, J = 13.3, 12.7, 6.7 Hz, 2H), 1.34 – 1.23 (m, 12H), 0.88 (t, J = 7.0 Hz, 3H).

^{13}C NMR (176 MHz, $CDCl_3$) δ 205.2, 169.0, 163.9 (t, J = 28.3 Hz), 117.0 (t, J = 255.5 Hz), 102.6, 96.4, 67.4, 67.4, 45.2, 38.4 (t, J = 24.0 Hz), 35.0, 31.9, 30.4, 29.5, 29.3, 29.3, 27.9, 27.8, 22.7, 19.5, 14.1.

^{19}F NMR (376 MHz, $CDCl_3$) δ -102.32, -102.65.

HRMS (ESI-TOF) m/z: $[M + H]^+$ calculated for $C_{22}H_{37}F_2N_2O_3$ 415.2767; Found 415.2771.

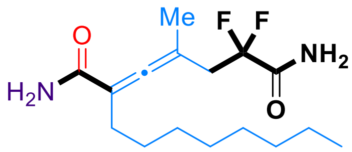

6,6-Difluoro-4-methyl-2-octylhepta-2,3-dienediamide (4x):

White solid, 12.1 mg, 38% yield, R_f=0.2 (PE/EtOAc 3/1).

^{1}H NMR (700 MHz, $CDCl_3$) δ 7.57 (s, 1H), 6.42 (d, J = 14.2 Hz, 2H), 6.30 (s, 1H), 2.90 (td, J = 15.8, 14.9, 6.7 Hz, 2H), 2.23 (dt, J = 15.2, 7.4 Hz, 1H), 2.16 (dt, J = 15.1, 7.2 Hz, 1H), 1.88 (s, 3H), 1.41 – 1.35 (m, 2H), 1.31 – 1.24 (m, 10H), 0.88 (t, J = 7.0 Hz, 3H).

^{13}C NMR (176 MHz, $CDCl_3$) δ 205.1, 169.9, 166.4 (t, J = 29.3 Hz), 116.6 (t, J = 254.3 Hz), 102.8, 96.9, 38.6 (t, J = 24.1 Hz), 31.9, 29.4, 29.3, 29.2, 27.9, 27.6, 22.7, 19.2, 14.1.

^{19}F NMR (376 MHz, $CDCl_3$) δ -101.54, -101.57.

HRMS (ESI-TOF) m/z: $[M + H]^+$ calculated for $C_{16}H_{27}F_2N_2O_2$ 317.2035; Found 317.2035.

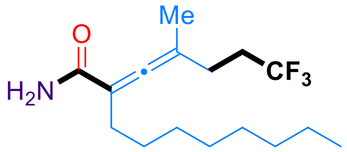

2-(5,5,5-Trifluoro-2-methylpent-1-en-1-ylidene)decanamide (4y):

Brown oil, 13.2 mg, 43% yield, R_f=0.2 (PE/EtOAc 3/1).

^{1}H NMR (700 MHz, $CDCl_3$) δ 5.79 (s, 1H), 5.42 (s, 1H), 2.38 – 2.31 (m, 2H), 2.24 (tt, J = 13.0, 6.9 Hz, 4H), 1.86 (s, 3H), 1.38 (p, J = 7.4, 6.8 Hz, 2H), 1.32 – 1.25 (m, 10H), 0.88 (t, J = 7.1 Hz, 3H).

^{13}C NMR (176 MHz, $CDCl_3$) δ 202.5, 168.9, 126.7 (q, J = 276.4 Hz), 103.7, 103.4, 31.9, 32.8 (q, J = 28.8 Hz), 29.5, 29.3, 29.3, 28.2, 27.8, 26.3 (q, J = 3.0 Hz), 22.7, 18.7, 14.1.

^{19}F NMR (376 MHz, $CDCl_3$) δ -66.31.

HRMS (ESI-TOF) m/z: $[M + H]^+$ calculated for $C_{16}H_{27}F_3NO$ 306.2039; Found 306.2038.

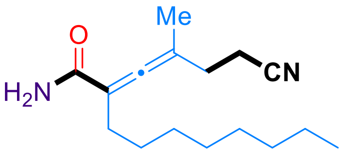

2-(4-Cyano-2-methylbut-1-en-1-ylidene)decanamide (4z):

Brown oil, 14.8 mg, 56% yield, R_f=0.2 (PE/EtOAc 3/1).

^{1}H NMR (700 MHz, $CDCl_3$) δ 6.12 (s, 1H), 5.33 (s, 1H), 2.58 – 2.45 (m, 3H), 2.43 – 2.38 (m, 1H), 2.29 (hept, J = 8.2, 7.7 Hz, 2H), 1.87 (s, 3H), 1.40 (dq, J = 12.7, 7.2 Hz, 2H), 1.29 (ddd, J = 23.2, 11.3, 5.3 Hz, 10H), 0.88 (t, J = 7.0 Hz, 3H).

^{13}C NMR (176 MHz, $CDCl_3$) δ 202.5, 168.4, 119.6, 104.5, 102.4, 31.9, 29.8, 29.5, 29.3, 29.3, 28.3, 28.1, 22.7, 18.5, 15.7, 14.1.

HRMS (ESI-TOF) m/z: $[M + H]^+$ calculated for $C_{16}H_{27}N_2O$ 263.2118; Found 263.2115.

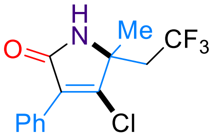

4-Chloro-5-methyl-3-phenyl-5-(2,2,2-trifluoroethyl)-1,5-dihydro-2H-pyrrol-2-one (5a):

White solid, 15.3 mg, 53% yield, R_f=0.2 (PE/EtOAc 20/1).

^{1}H NMR (700 MHz, $CDCl_3$) δ 7.79 (dd, J = 8.2, 1.3 Hz, 2H), 7.49 – 7.44 (m, 3H), 2.90 – 2.83 (m, 1H), 2.74 – 2.68 (m, 1H), 1.71 (s, 3H).

^{13}C NMR (176 MHz, $CDCl_3$) δ 167.6, 153.8, 129.9, 128.6, 128.6, 127.2, 126.3, 124.3 (q, J = 278.2 Hz), 82.5 (d, J = 2.1 Hz), 40.2 (q, J = 28.9 Hz), 24.4.

HRMS (ESI-TOF) m/z: $[M + H]^+$ calculated for $C_{13}H_{12}ClF_3NO$ 290.0554; Found 290.0557.

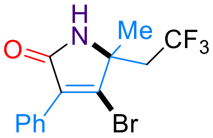

4-Bromo-5-methyl-3-phenyl-5-(2,2,2-trifluoroethyl)-1,5-dihydro-2H-pyrrol-2-one (5b):

White solid, 21.4 mg, 64% yield, R_f=0.2 (PE/EtOAc 20/1).

^{1}H NMR (700 MHz, $CDCl_3$) δ 7.73 (dd, J = 7.9, 1.5 Hz, 2H), 7.49 – 7.44 (m, 3H), 2.86 (dq, J = 15.7, 10.0 Hz, 1H), 2.72 (dq, J = 15.7, 9.8 Hz, 1H), 1.70 (s, 3H).

^{13}C NMR (176 MHz, $CDCl_3$) δ 167.8, 145.9, 130.3, 129.9, 128.7, 128.5, 128.0, 124.4 (q, J = 278.3 Hz), 83.7 (q, J = 2.0 Hz), 40.6 (q, J = 28.8 Hz), 24.8.

HRMS (ESI-TOF) m/z: $[M + H]^+$ calculated for $C_{13}H_{12}BrF_3NO$ 334.0049; Found 334.0051.

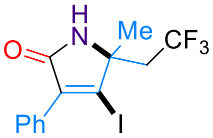

4-Iodo-5-methyl-3-phenyl-5-(2,2,2-trifluoroethyl)-1,5-dihydro-2H-pyrrol-2-one (5c):

Yellow solid, 23.6 mg, 62% yield, R_f=0.2 (PE/EtOAc 10/1).

^{1}H NMR (700 MHz, $CDCl_3$) δ 7.54 – 7.49 (m, 2H), 7.45 – 7.39 (m, 3H), 2.85 (dq, J = 15.7, 10.0 Hz, 1H), 2.73 (dq, J = 15.6, 9.9 Hz, 1H), 1.71 (s, 3H).

^{13}C NMR (176 MHz, $CDCl_3$) δ 170.7, 136.5, 130.4, 129.6, 129.2, 128.4, 124.5 (q, J = 278.7 Hz), 117.5, 88.8 (q, J = 1.9 Hz),, 41.3 (q, J = 28.3 Hz), 25.7.

HRMS (ESI-TOF) m/z: $[M + H]^+$ calculated for $C_{13}H_{12}F_3INO$ 381.9910; Found 381.9907.

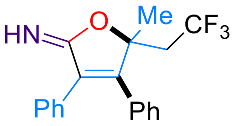

5-Methyl-3,4-diphenyl-5-(2,2,2-trifluoroethyl)furan-2(5H)-imine (5d):

Yellow solid, 16.2 mg, 49% yield, R_f=0.2 (PE/EtOAc 3/1).

^{1}H NMR (700 MHz, $CDCl_3$) δ 7.34 (dq, J = 6.5, 3.1 Hz, 3H), 7.27 (s, 5H), 7.17 (dd, J = 7.4, 2.1 Hz, 2H), 2.72 – 2.56 (m, 2H), 1.71 (s, 3H).

^{13}C NMR (176 MHz, $CDCl_3$) δ 156.0, 131.8, 130.5, 129.5, 129.1, 128.9, 128.6, 128.6, 128.5, 125.1 (q, J = 278.4 Hz), 85.7 (q, J = 2.0 Hz), 41.4 (q, J = 27.8 Hz), 25.1.

HRMS (ESI-TOF) m/z: $[M + H]^+$ calculated for $C_{19}H_{17}F_3NO$ 332.1257; Found 332.1255.

6. NMR Spectra of the Products.

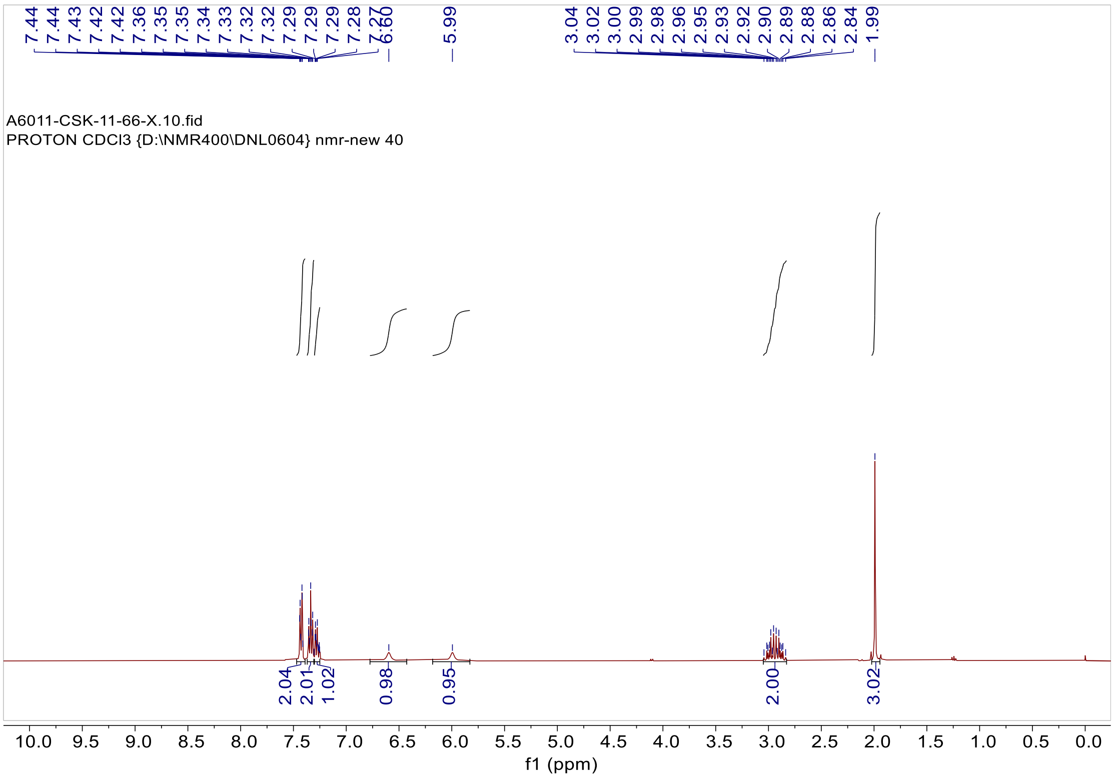

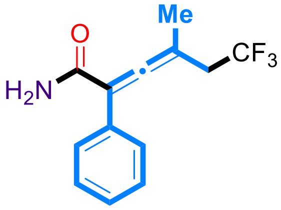

4a

^{1}H NMR (400 MHz, $CDCl_3$)
^{13}C NMR (100 MHz, $CDCl_3$)

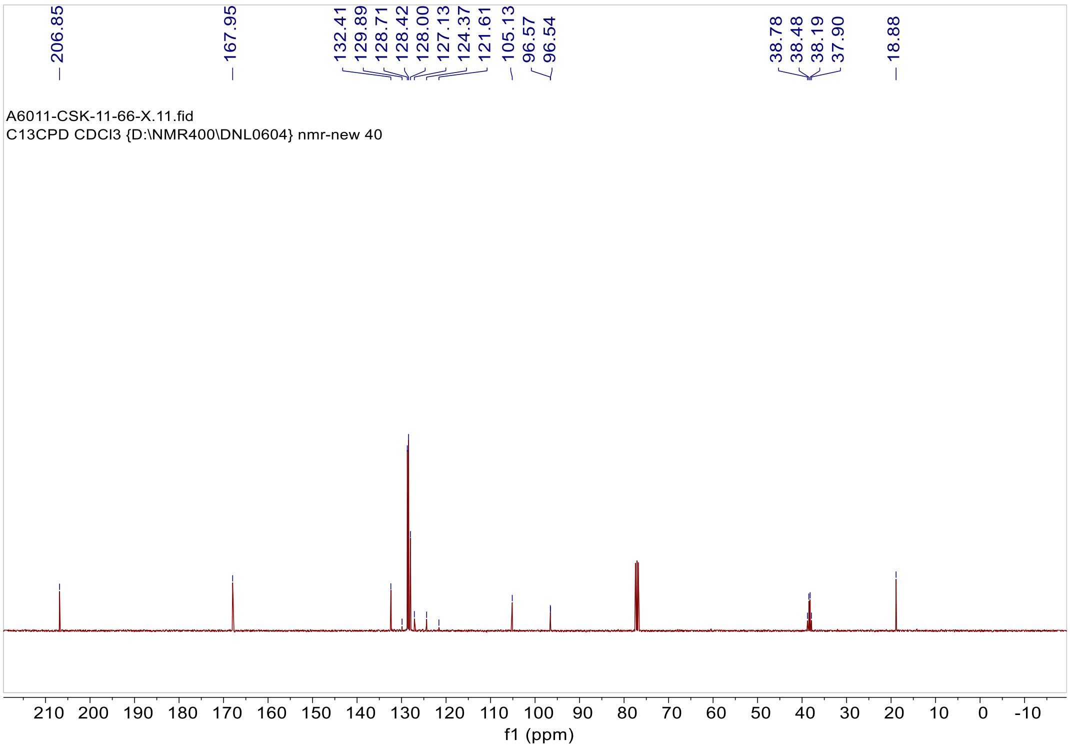

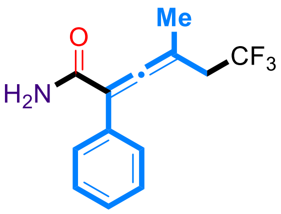

4a ^{19}F NMR (376 MHz, $CDCl_3$)

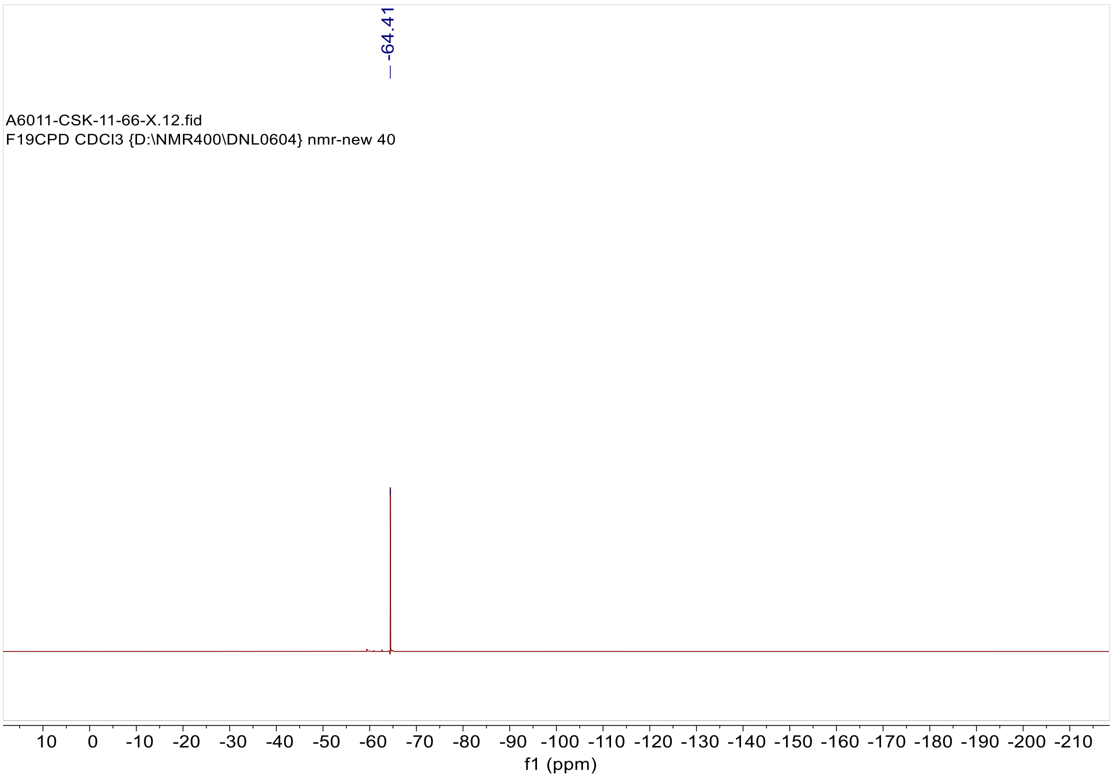

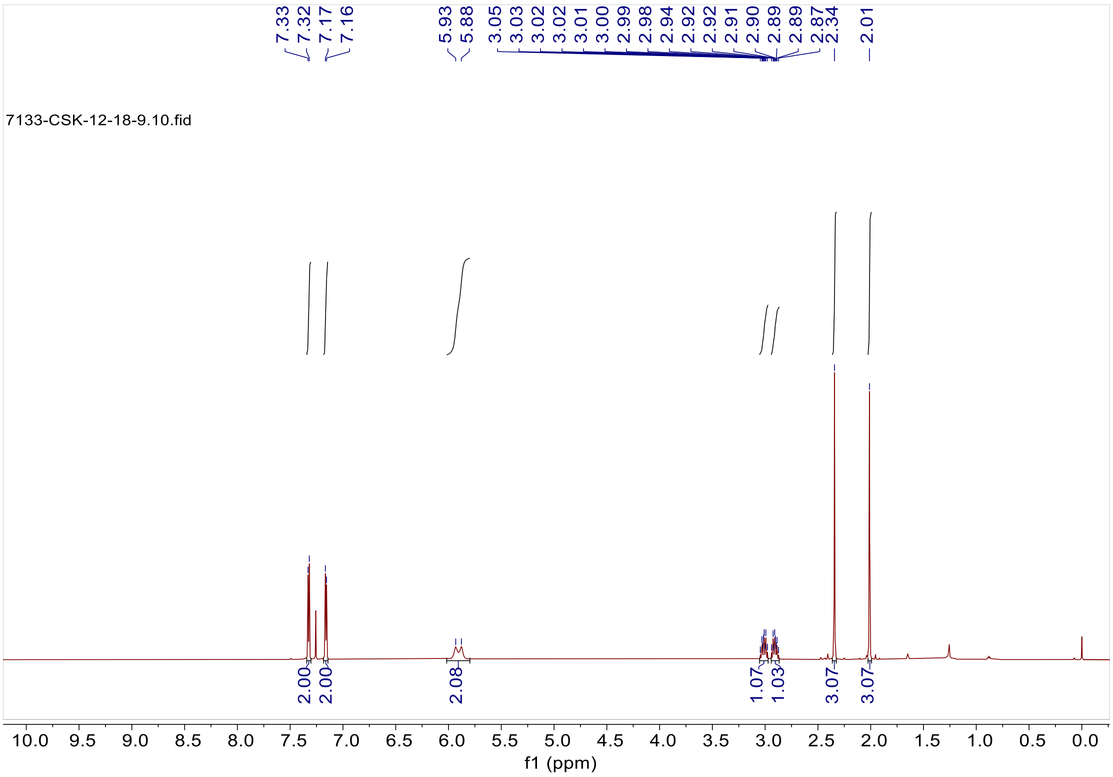

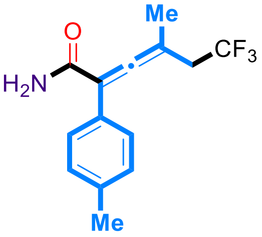

4b

^{1}H NMR (700 MHz, $CDCl_3$)

^{13}C NMR (176 MHz, $CDCl_3$)

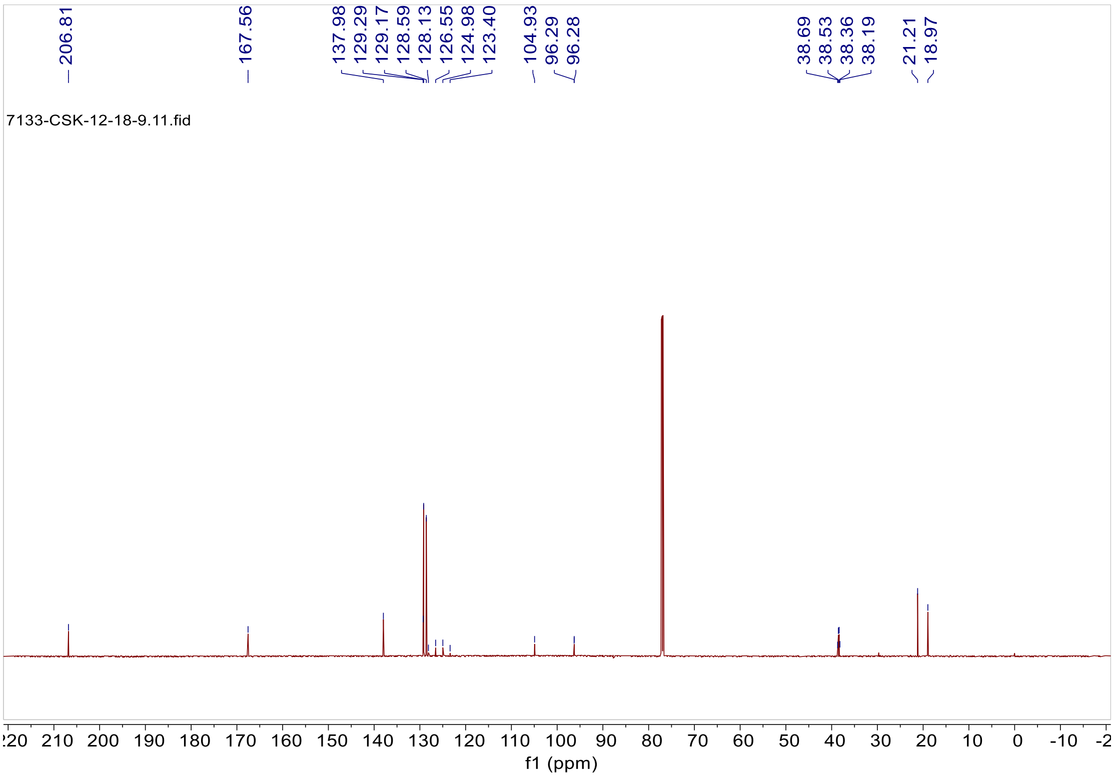

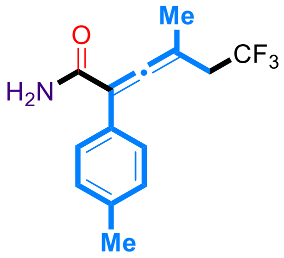

4b

^{19}F NMR (376 MHz, $CDCl_3$)

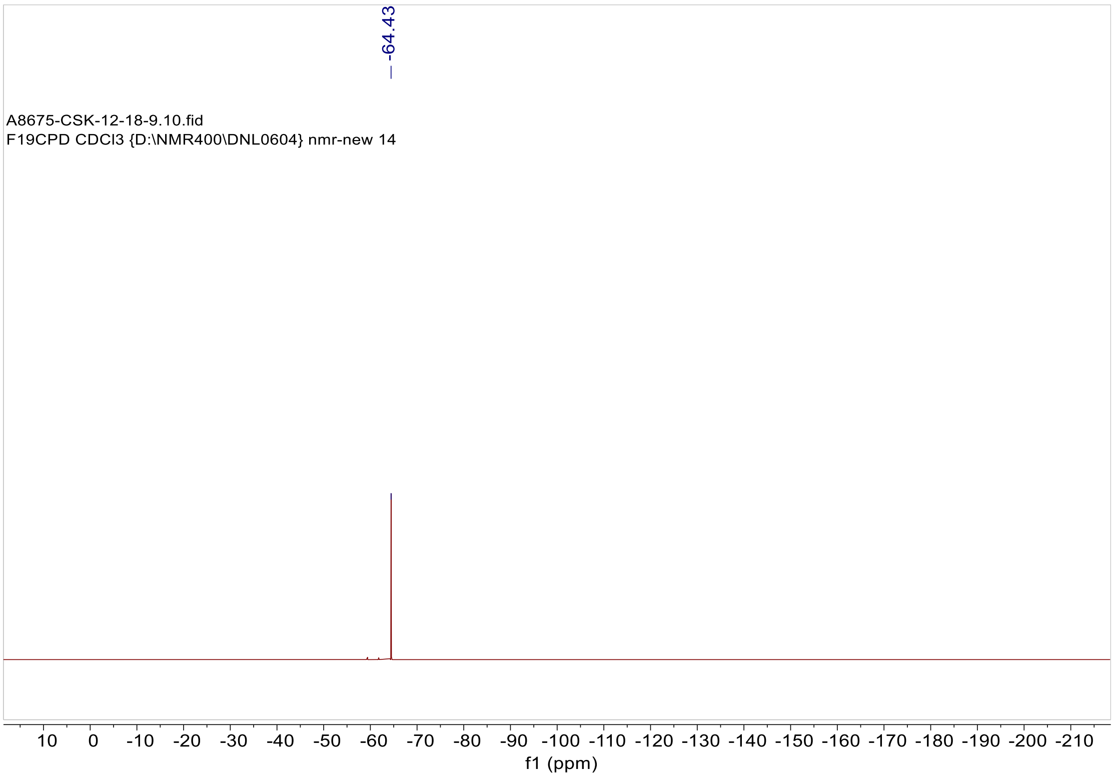

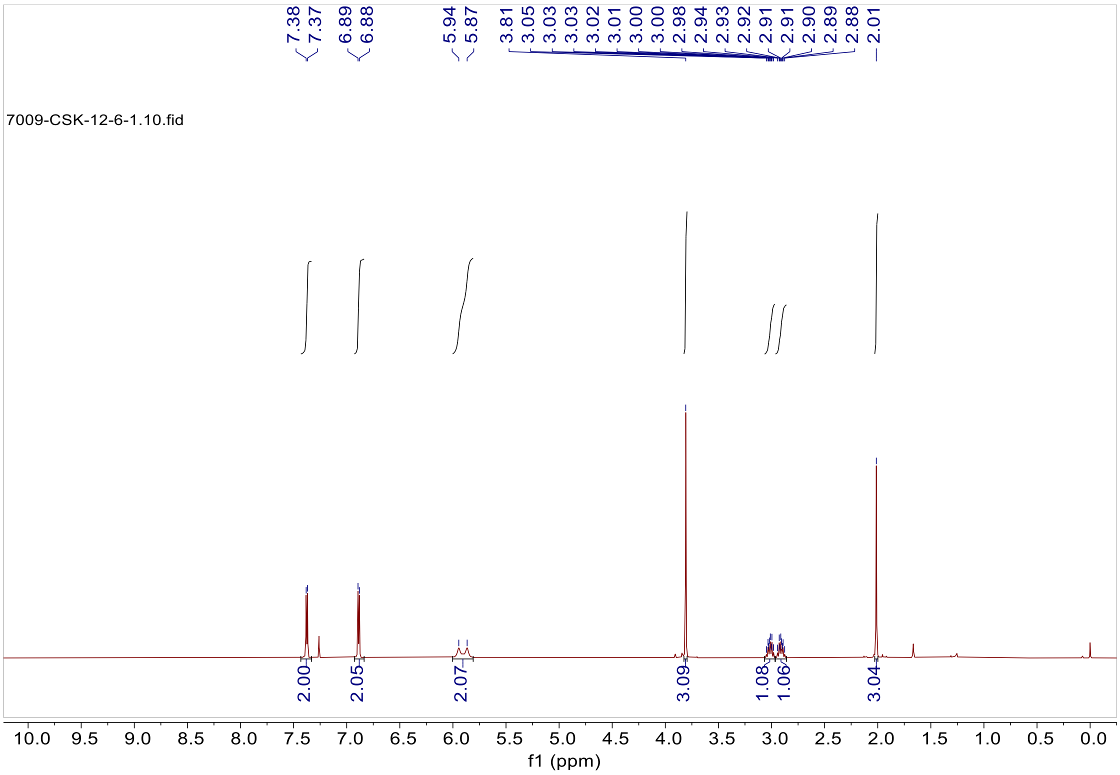

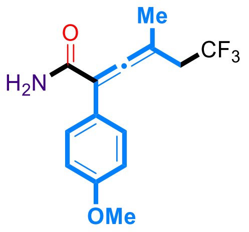

4c

^{1}H NMR (700 MHz, $CDCl_3$)

^{13}C NMR (176 MHz, $CDCl_3$)

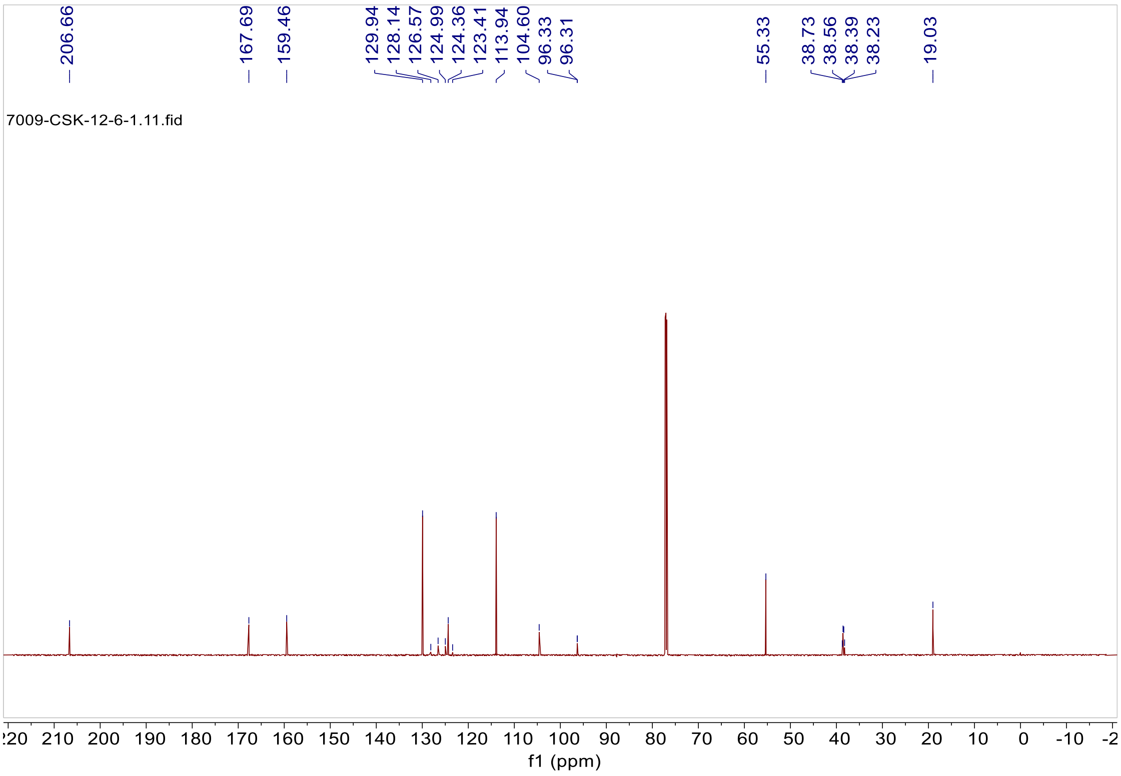

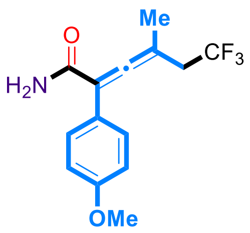

4c ^{19}F NMR (376 MHz, $CDCl_3$)

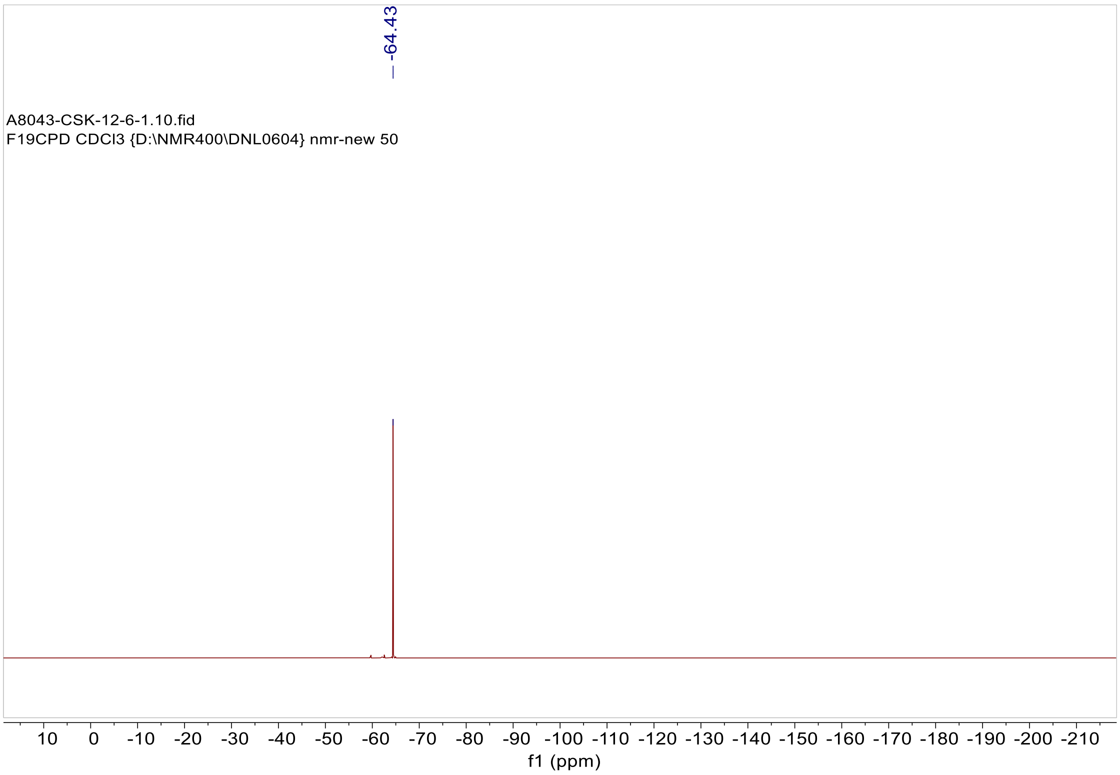

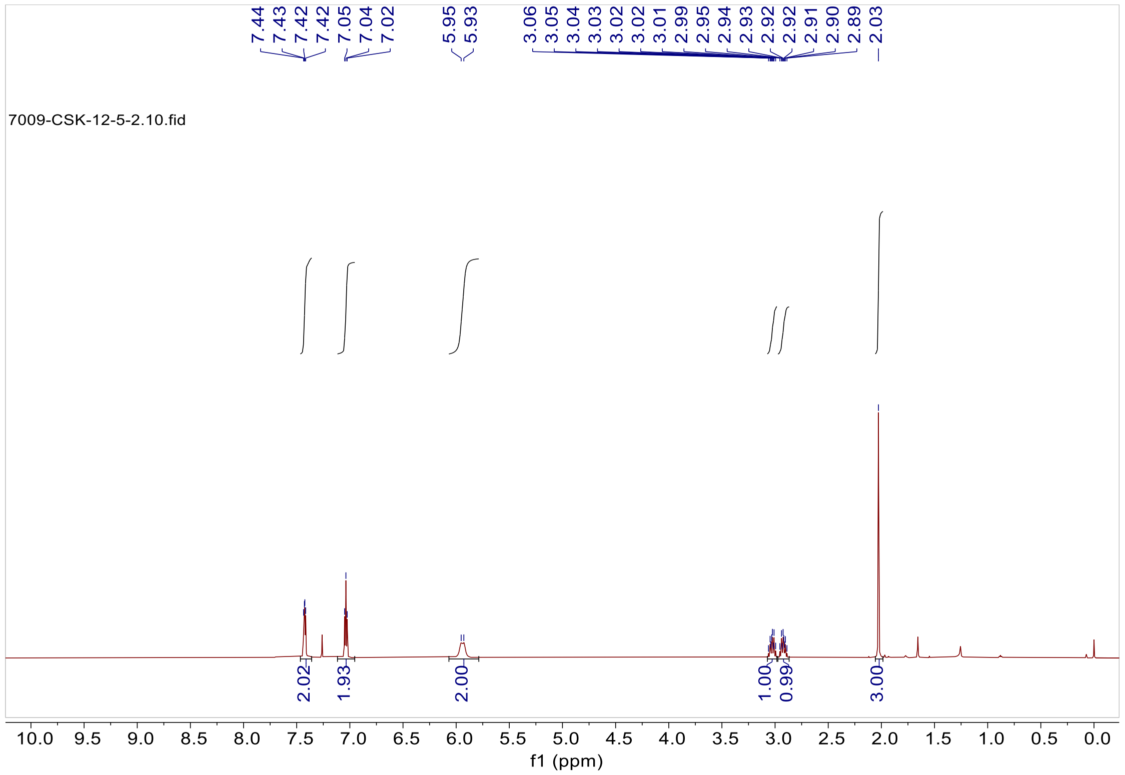

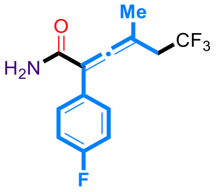

4d

^{1}H NMR (700 MHz, $CDCl_3$)

^{13}C NMR (176 MHz, $CDCl_3$)

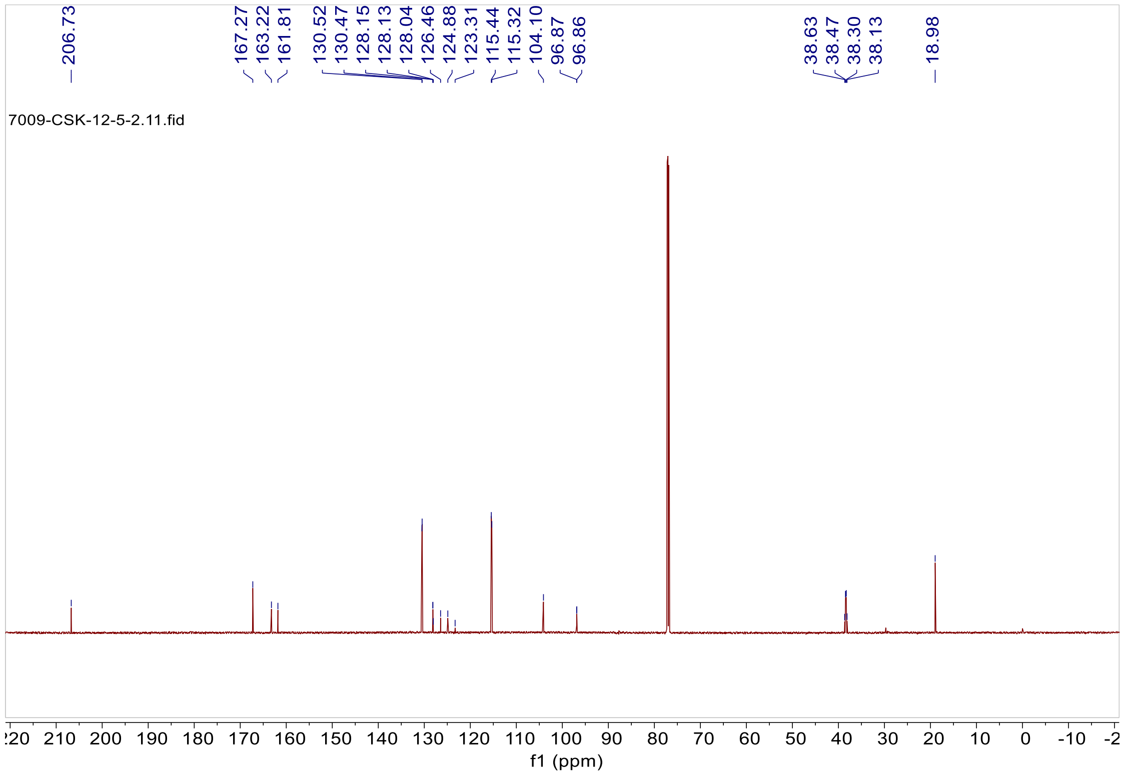

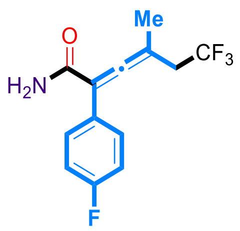

4d ^{19}F NMR (376 MHz, $CDCl_3$)

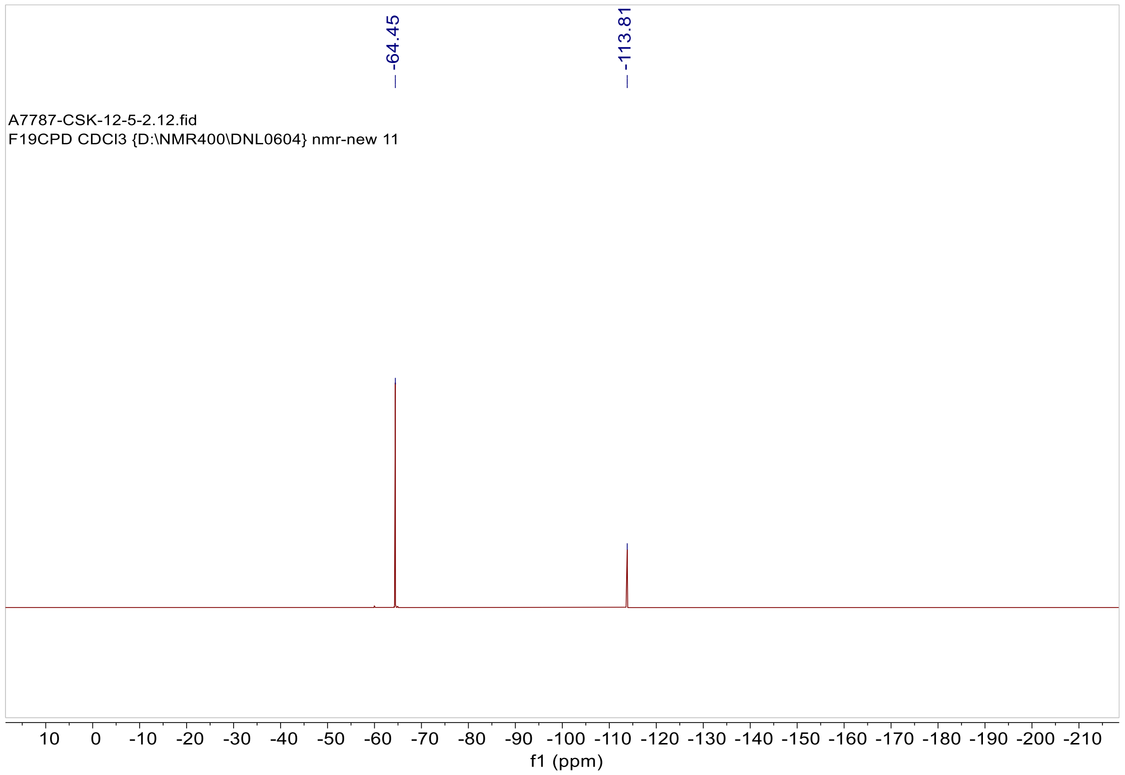

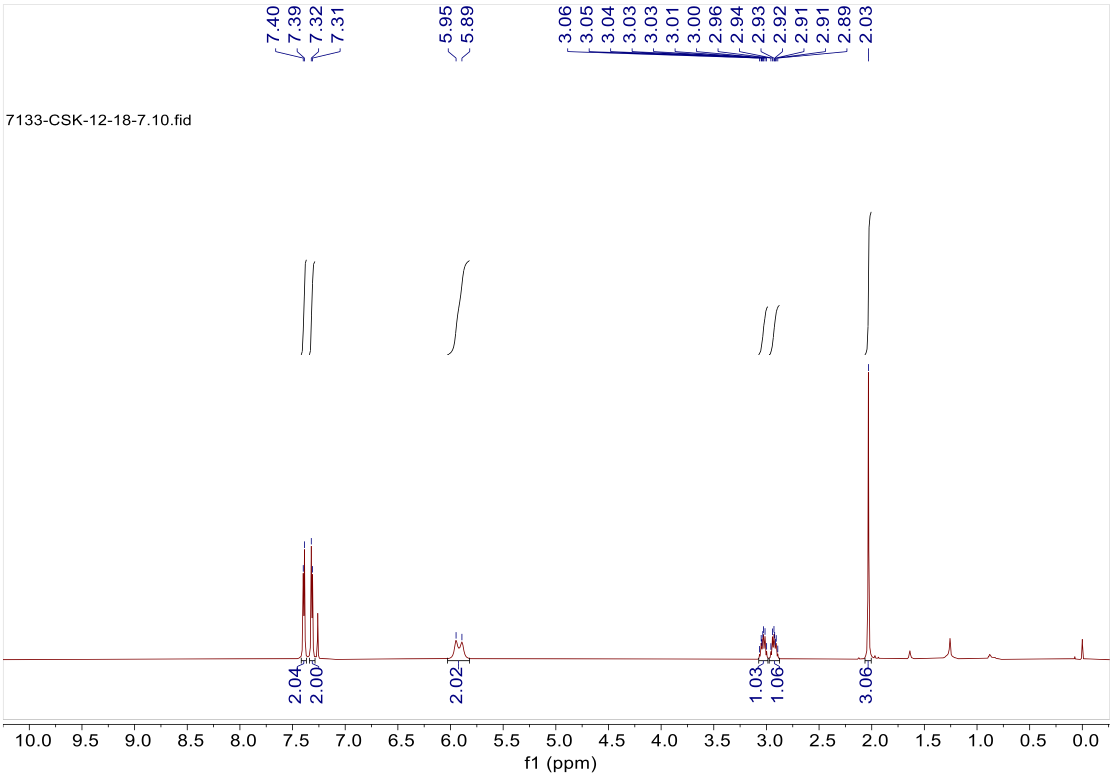

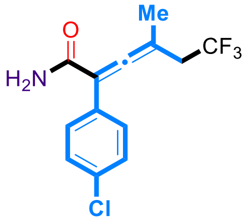

4e

^{1}H NMR (700 MHz, $CDCl_3$)

^{13}C NMR (176 MHz, $CDCl_3$)

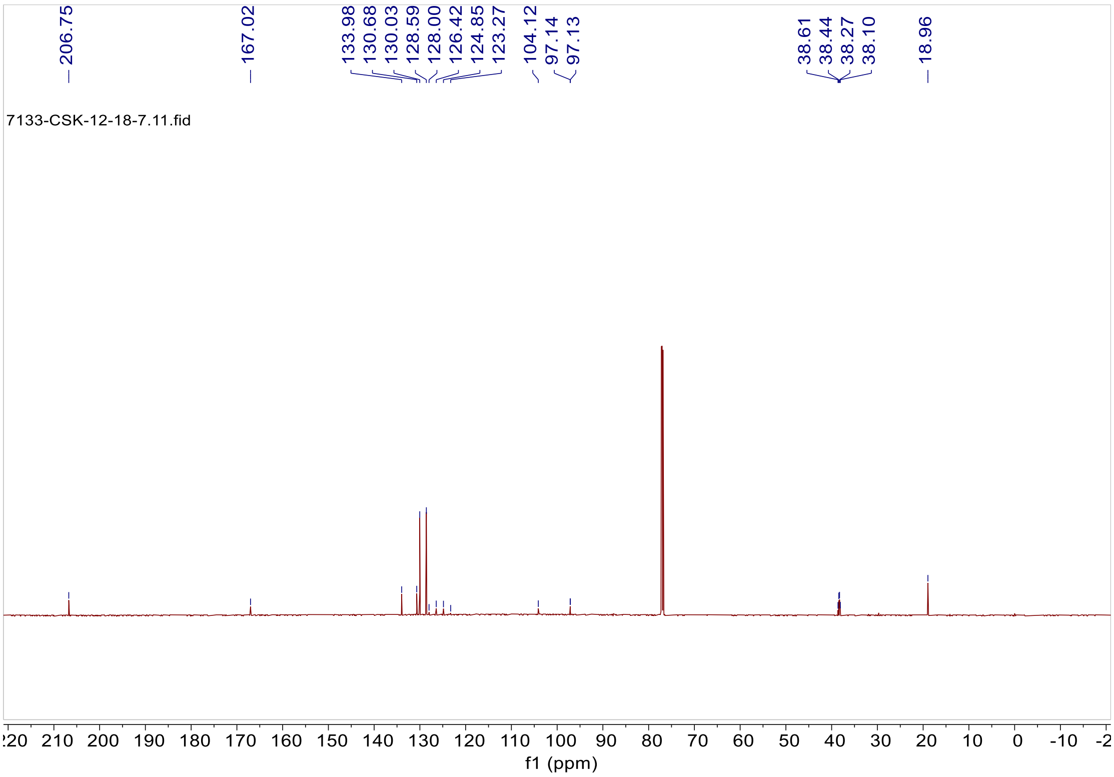

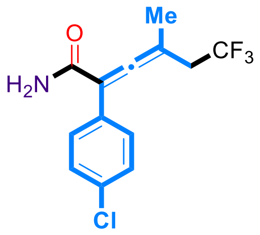

4e ^{19}F NMR (376 MHz, $CDCl_3$)

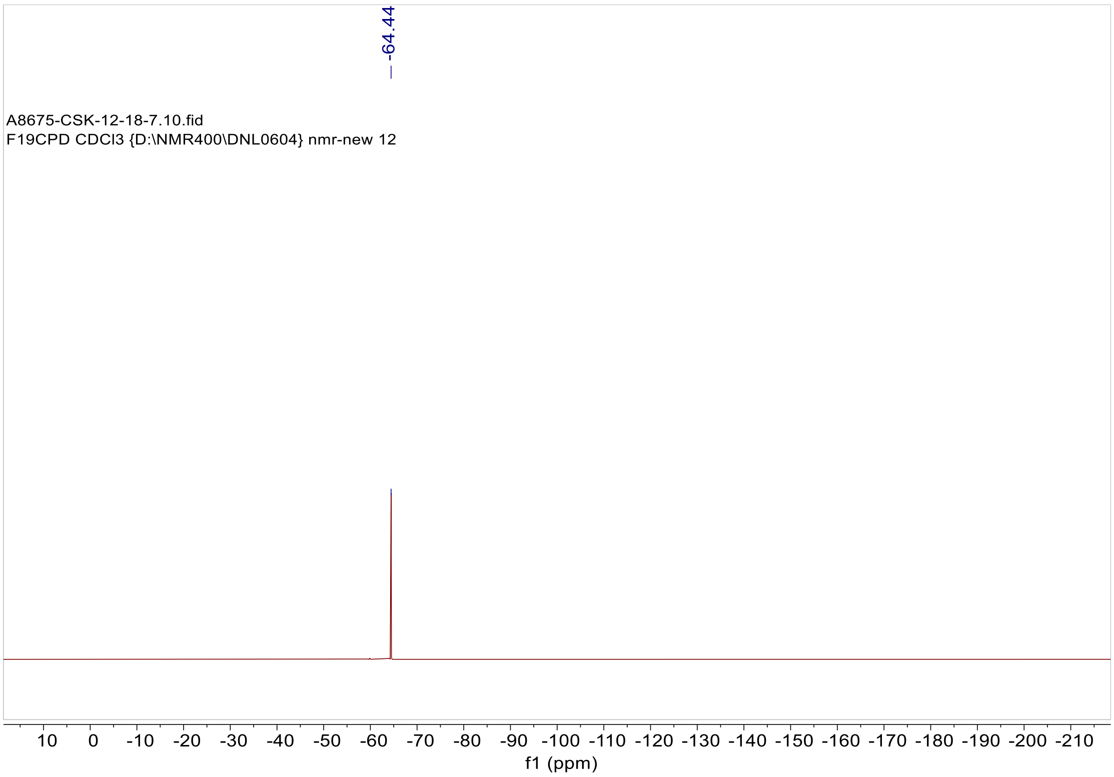

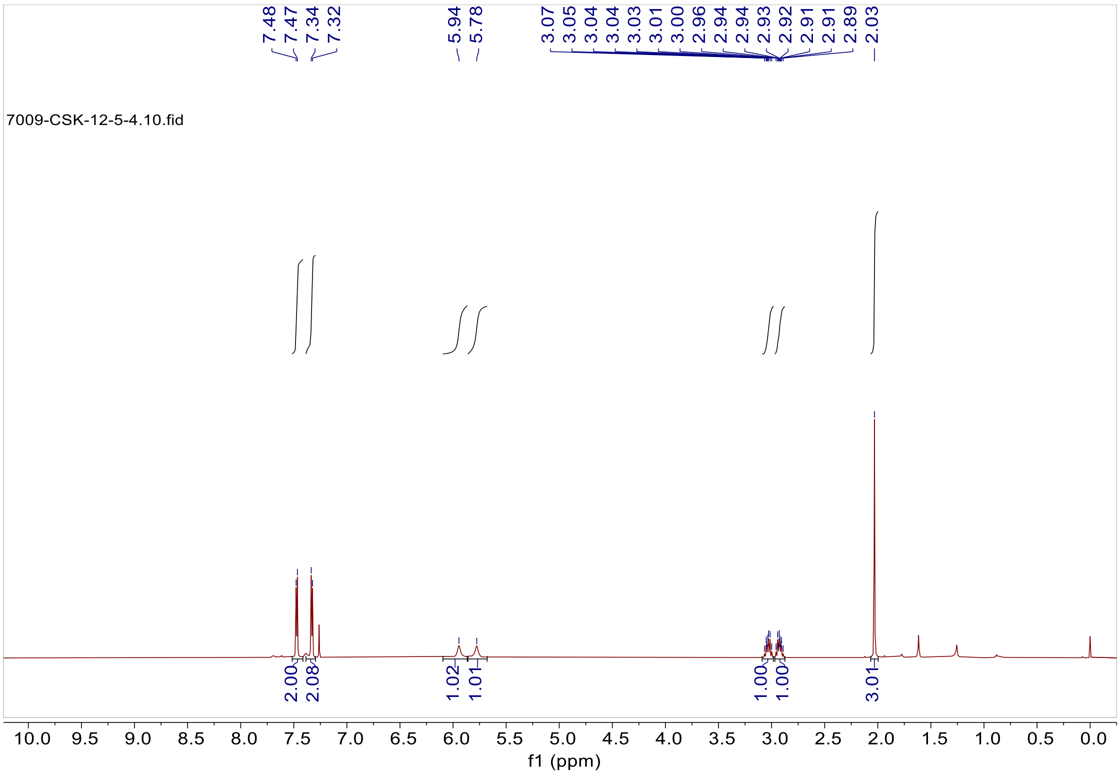

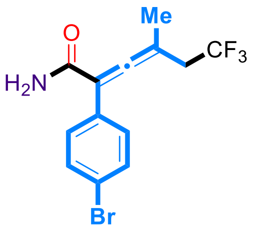

4f

^{1}H NMR (700 MHz, $CDCl_3$)

^{13}C NMR (176 MHz, $CDCl_3$)

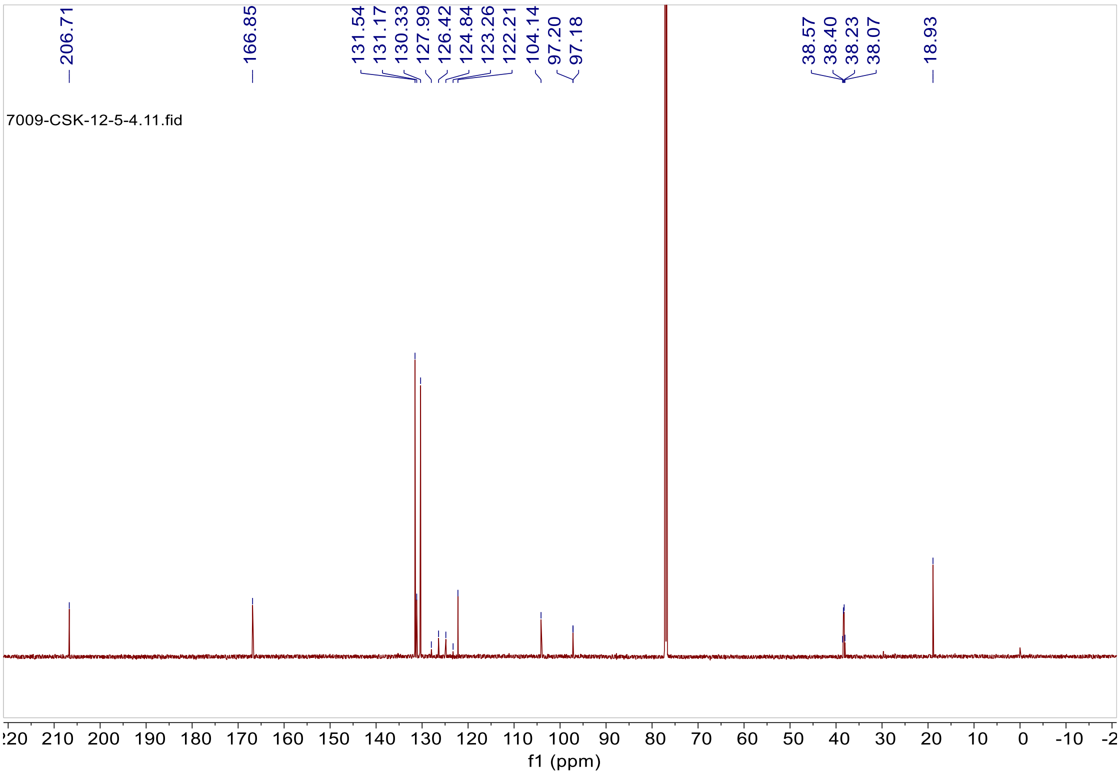

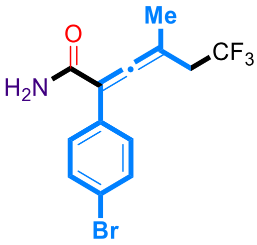

4f ^{19}F NMR (376 MHz, $CDCl_3$)

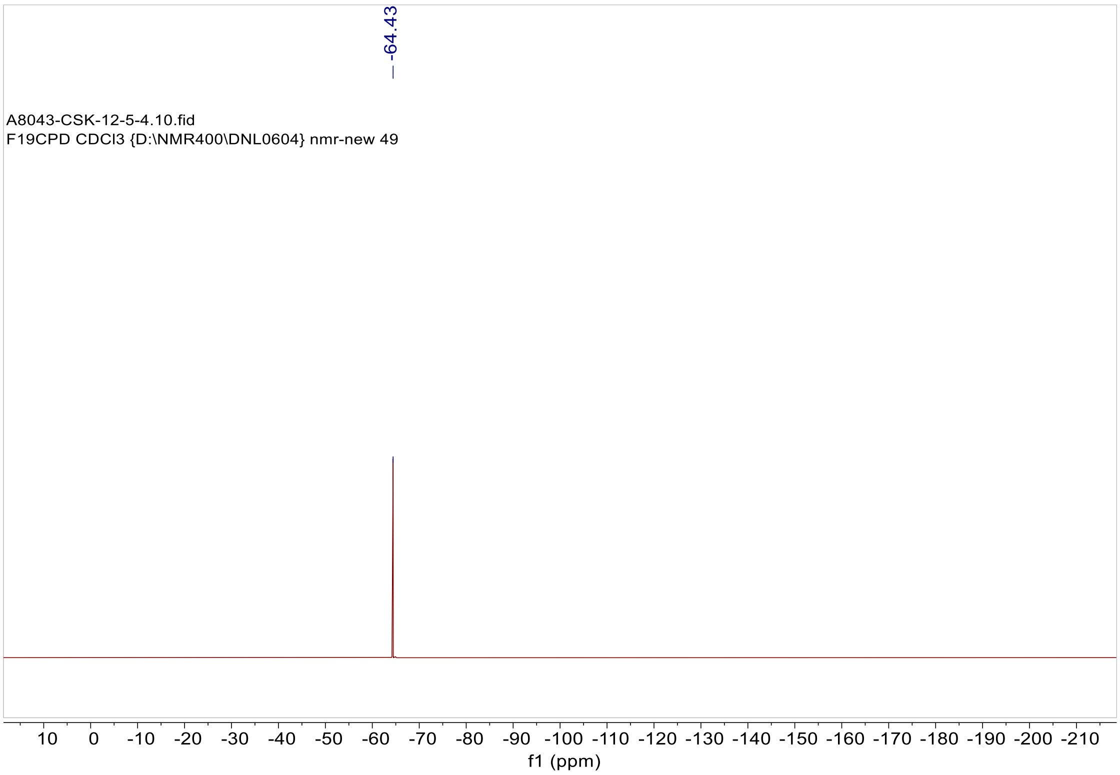

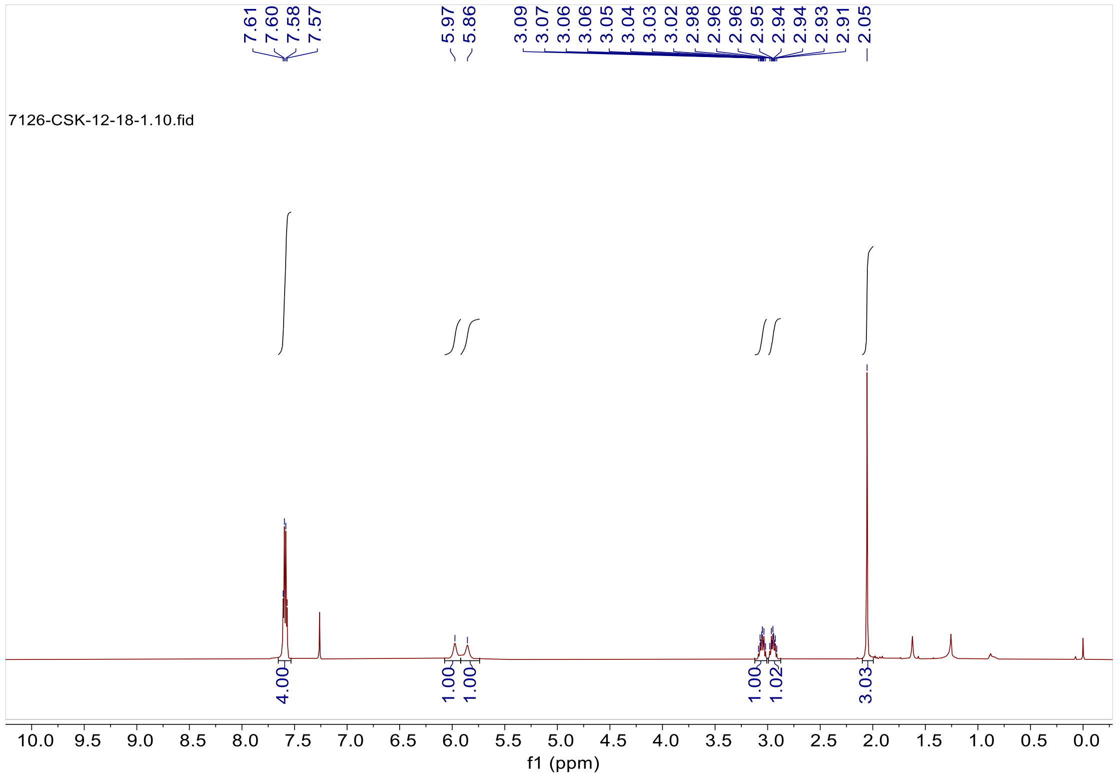

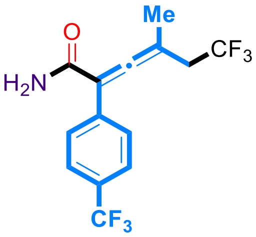

4g

^{1}H NMR (700 MHz, $CDCl_3$)
^{13}C NMR (176 MHz, $CDCl_3$)

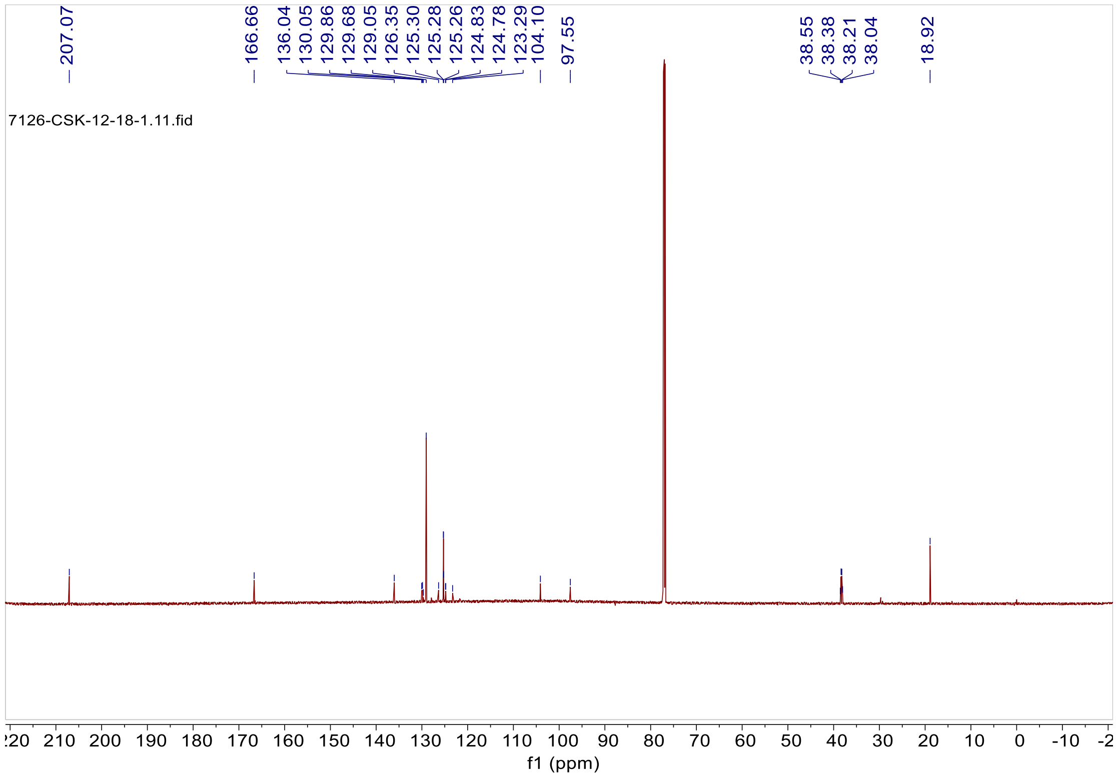

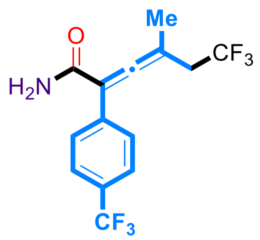

4g ^{19}F NMR (376 MHz, $CDCl_3$)

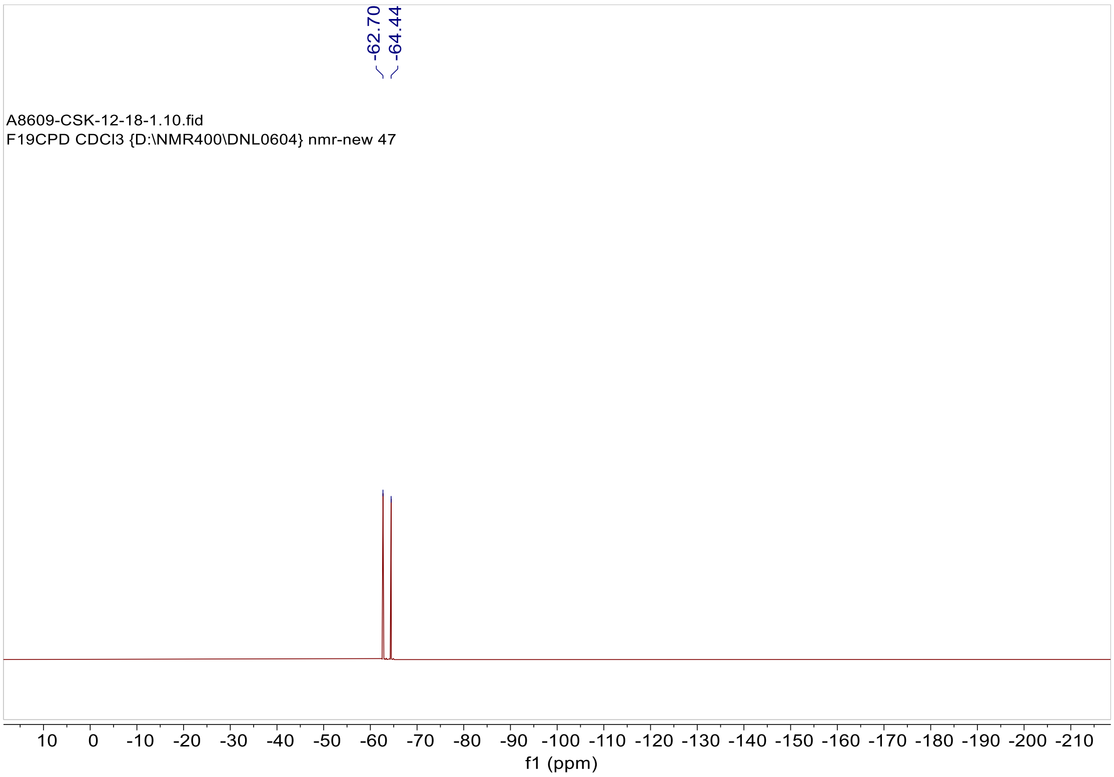

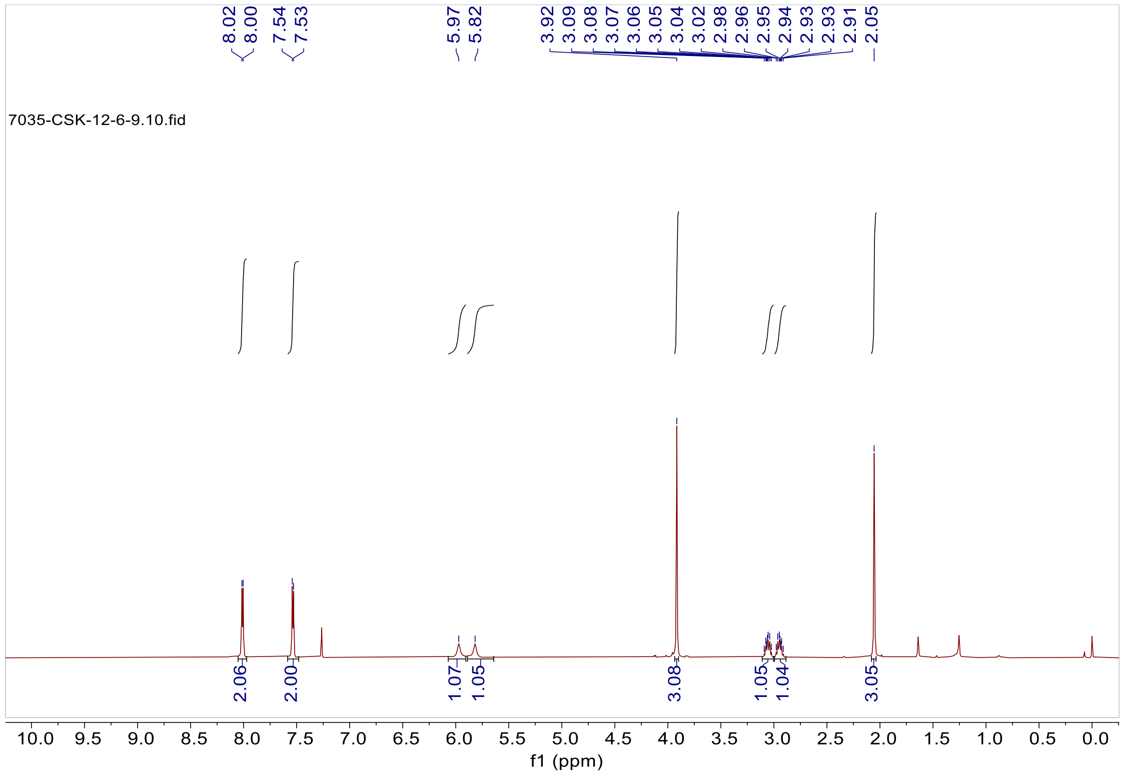

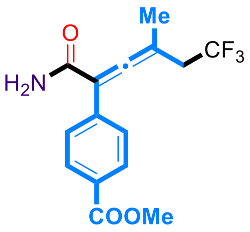

4h

^{1}H NMR (700 MHz, $CDCl_3$)

^{13}C NMR (176 MHz, $CDCl_3$)

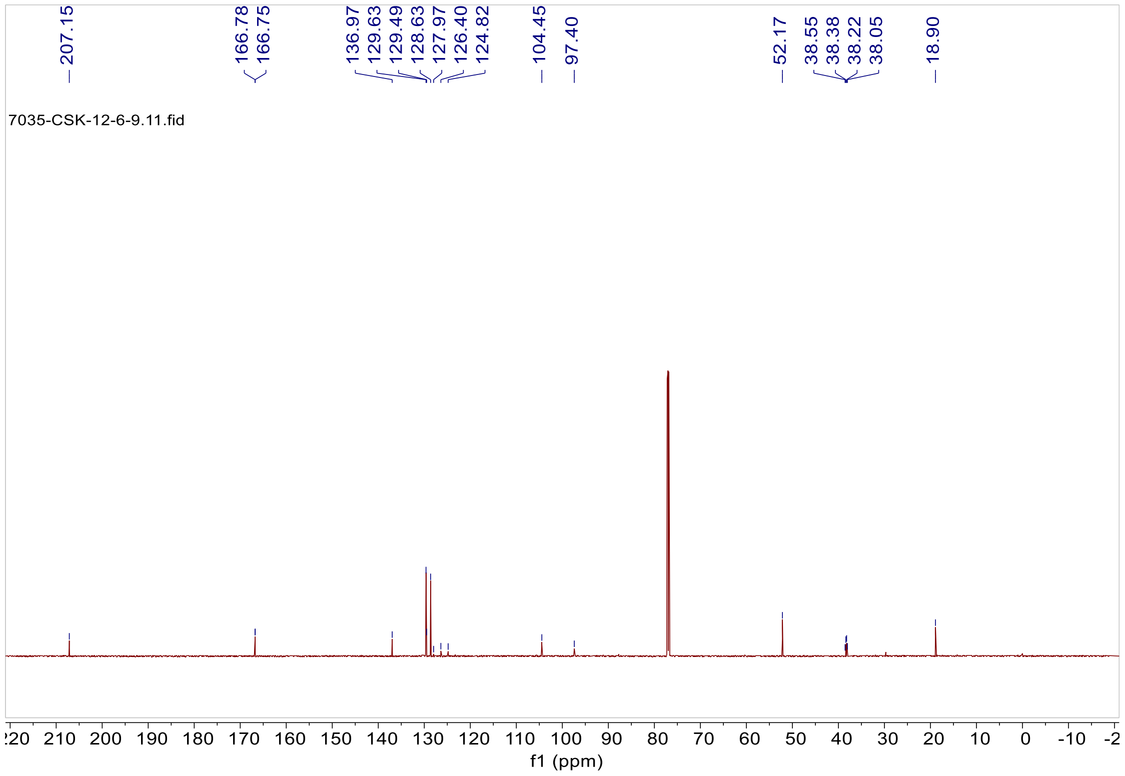

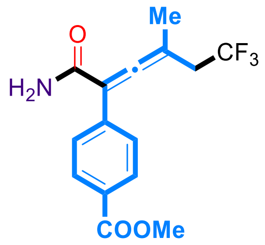

4h ^{19}F NMR (376 MHz, $CDCl_3$)

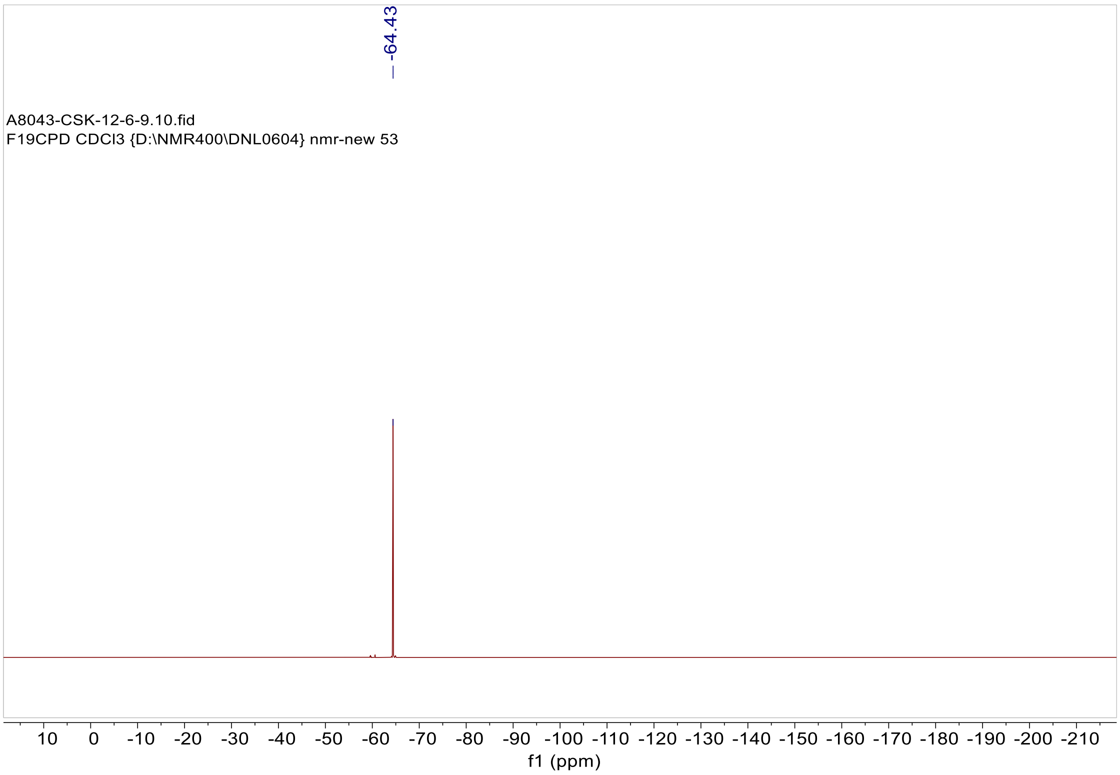

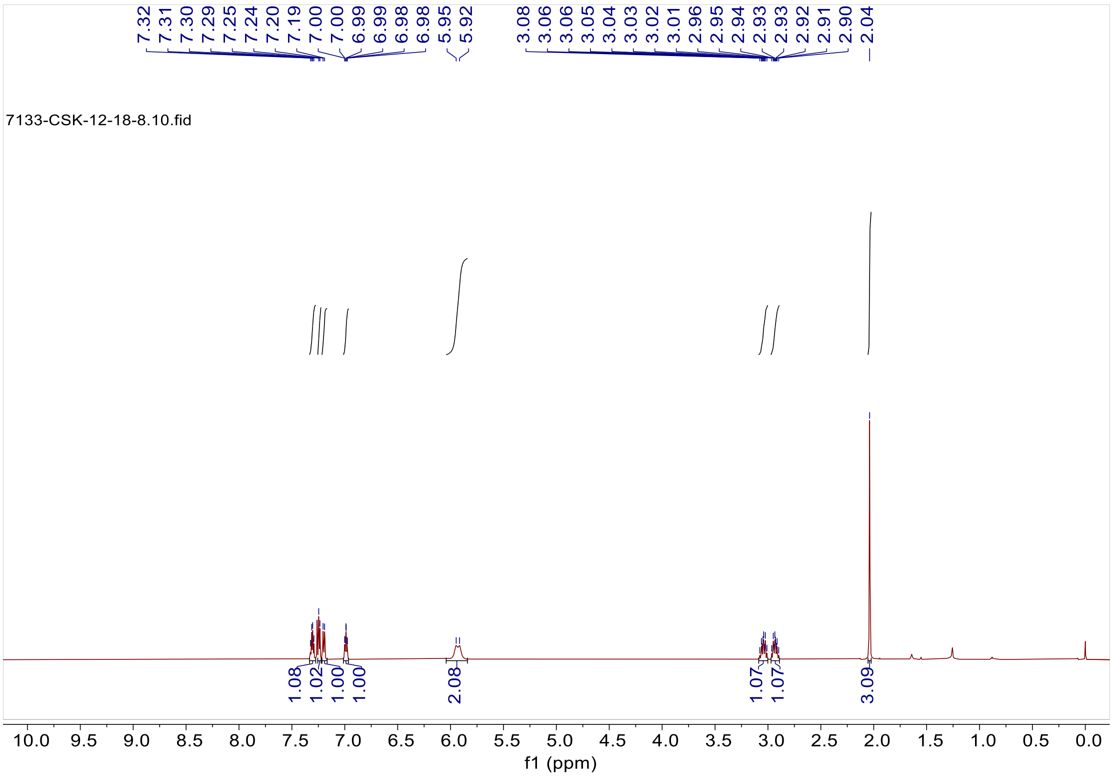

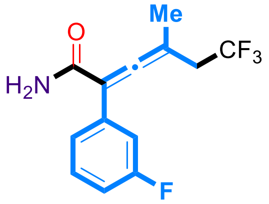

4j

^{1}H NMR (700 MHz, $CDCl_3$)

^{13}C NMR (176 MHz, $CDCl_3$)

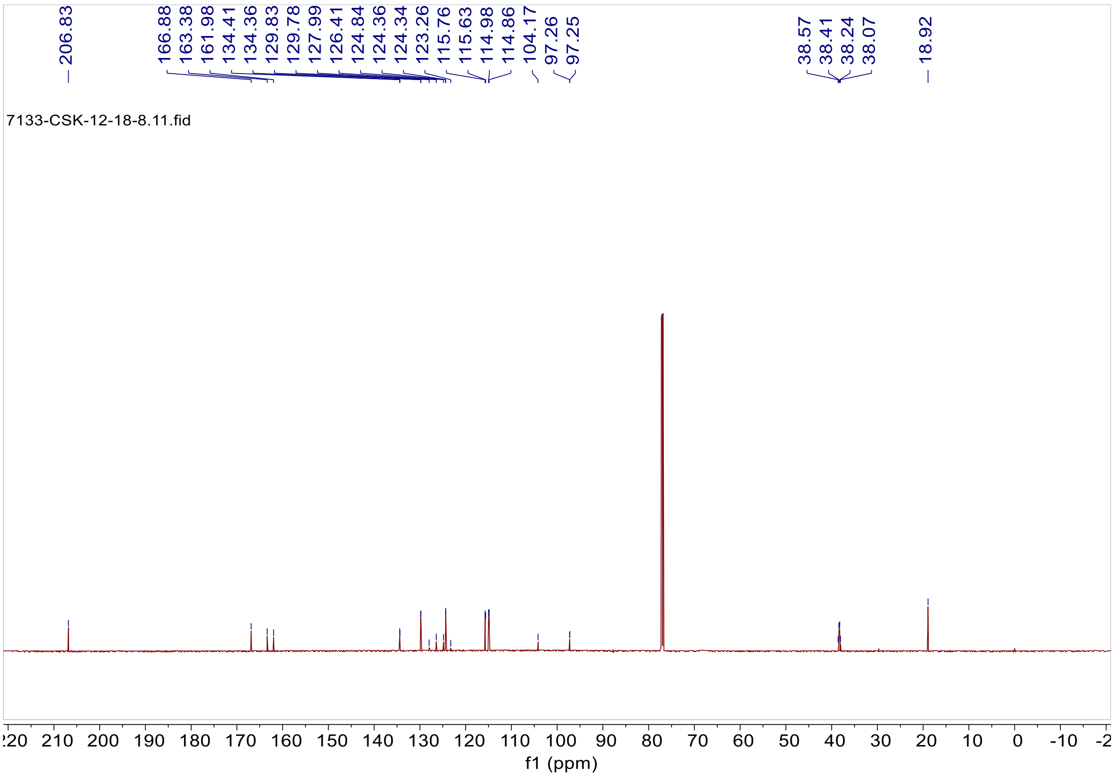

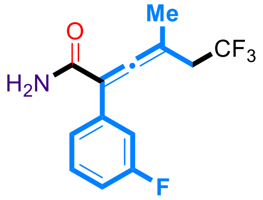

4j ^{19}F NMR (376 MHz, $CDCl_3$)

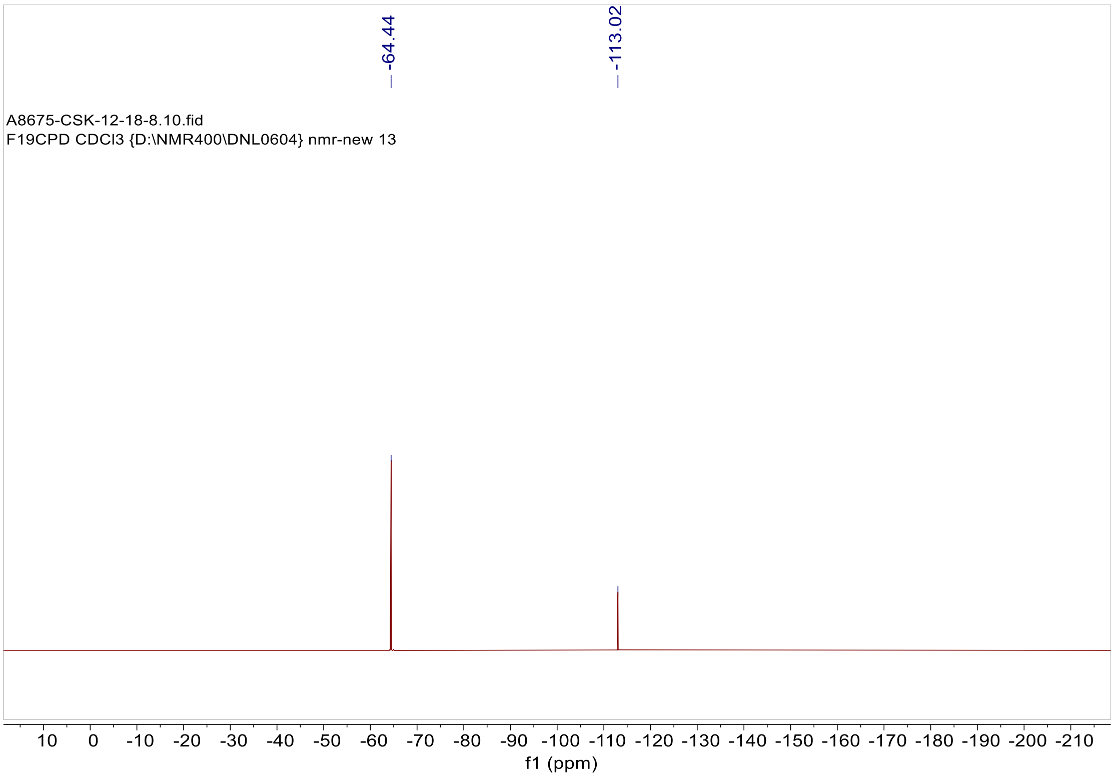

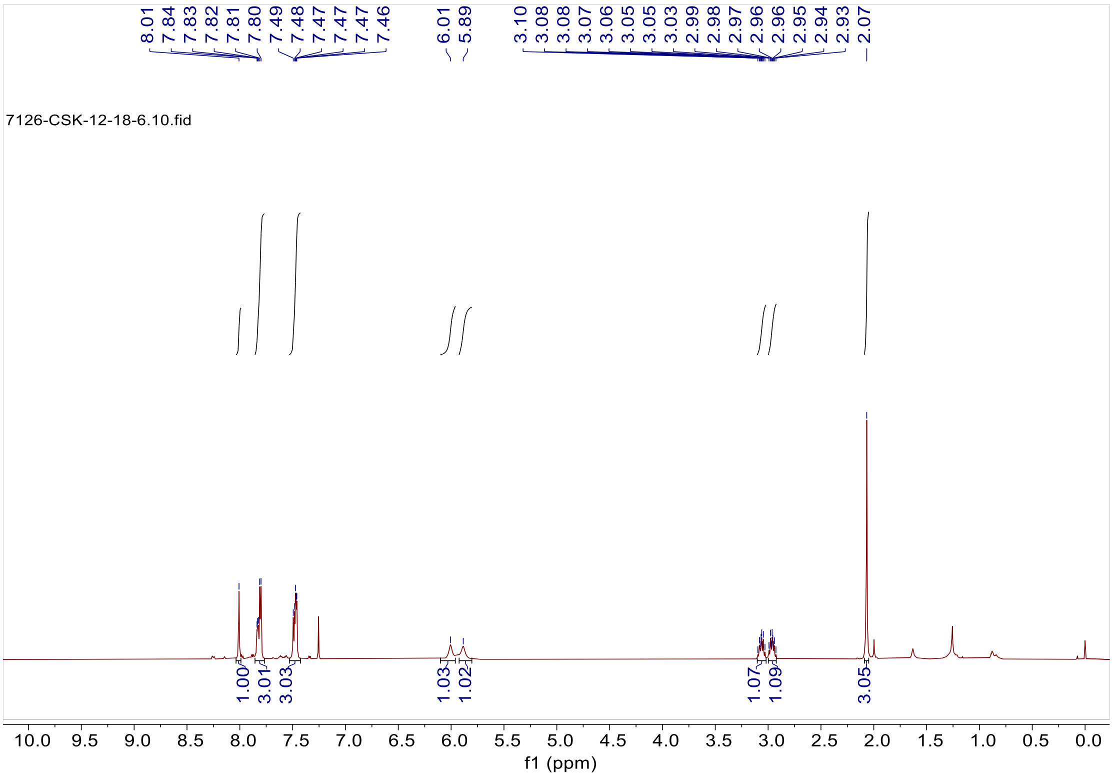

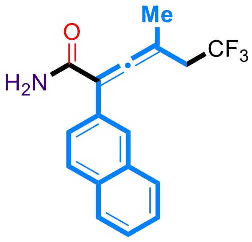

4k

^{1}H NMR (700 MHz, $CDCl_3$)

^{13}C NMR (176 MHz, $CDCl_3$)

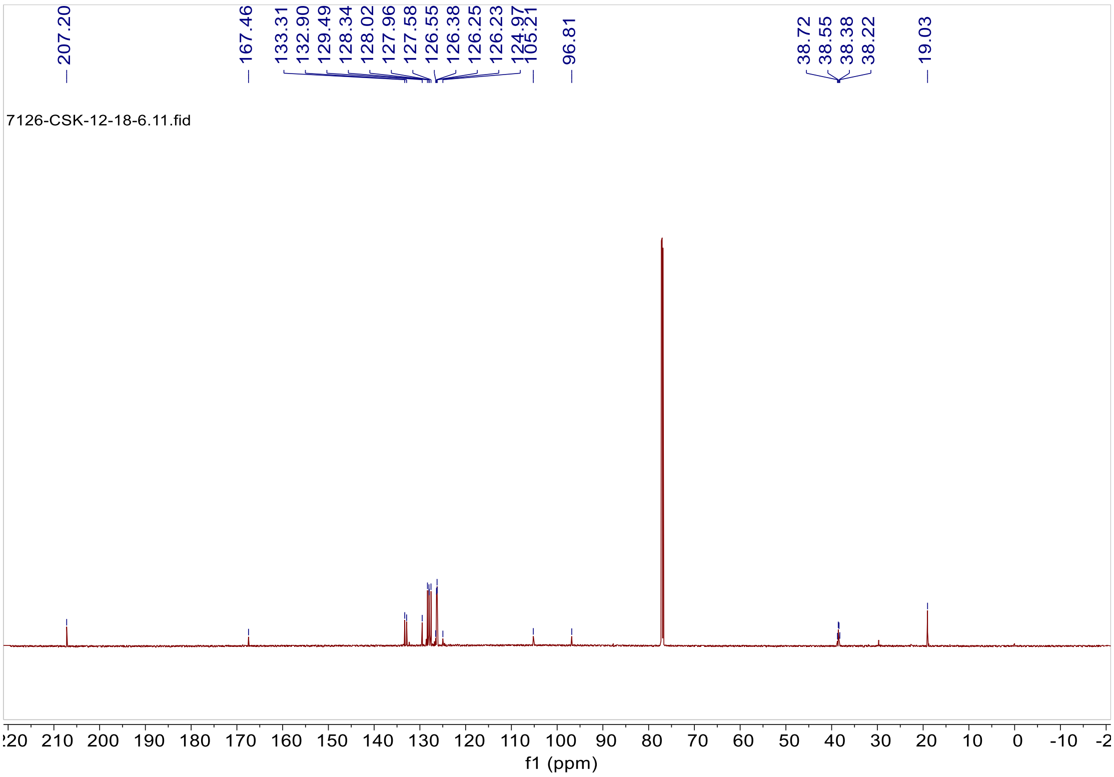

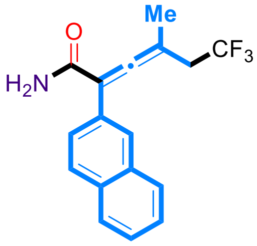

4k ^{19}F NMR (376 MHz, $CDCl_3$)

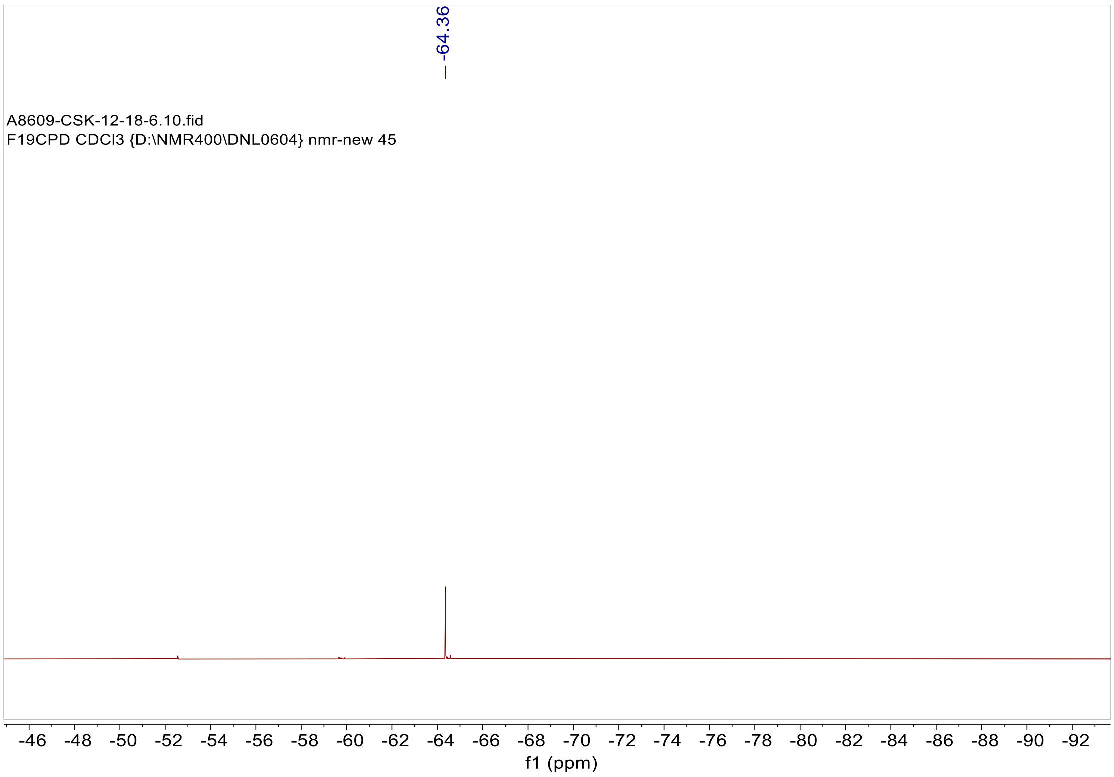

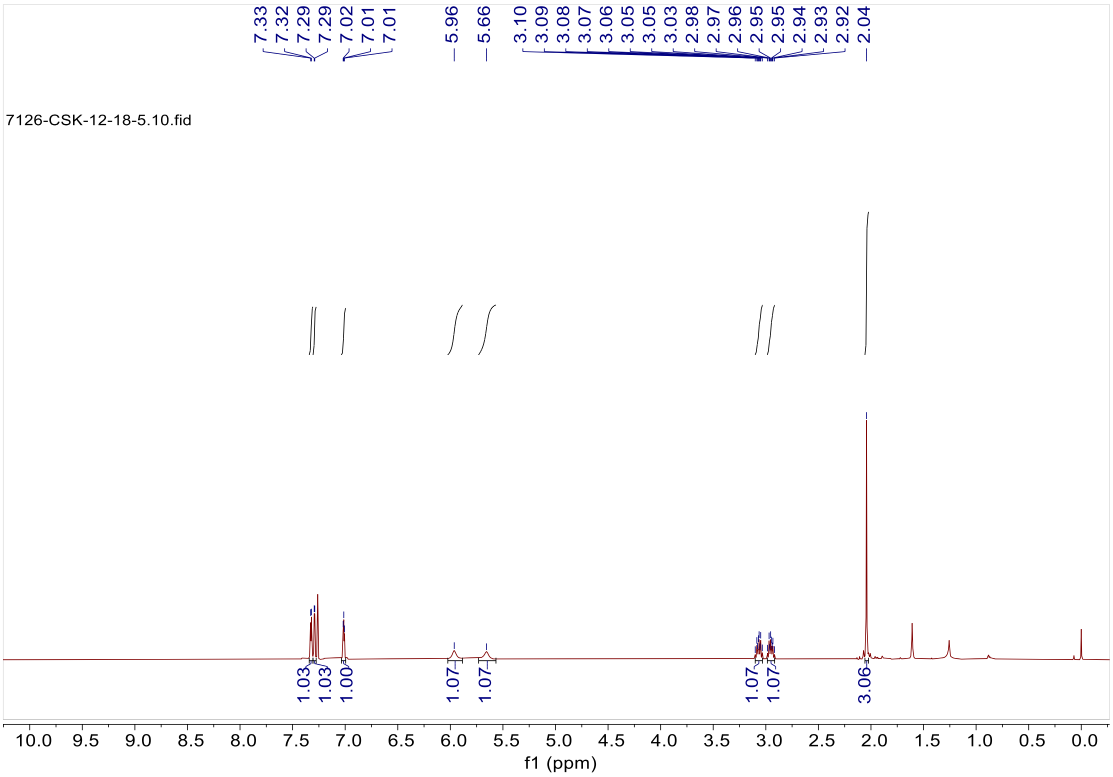

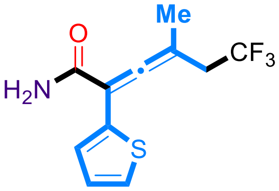

4l

^{1}H NMR (700 MHz, $CDCl_3$)
^{13}C NMR (176 MHz, $CDCl_3$)

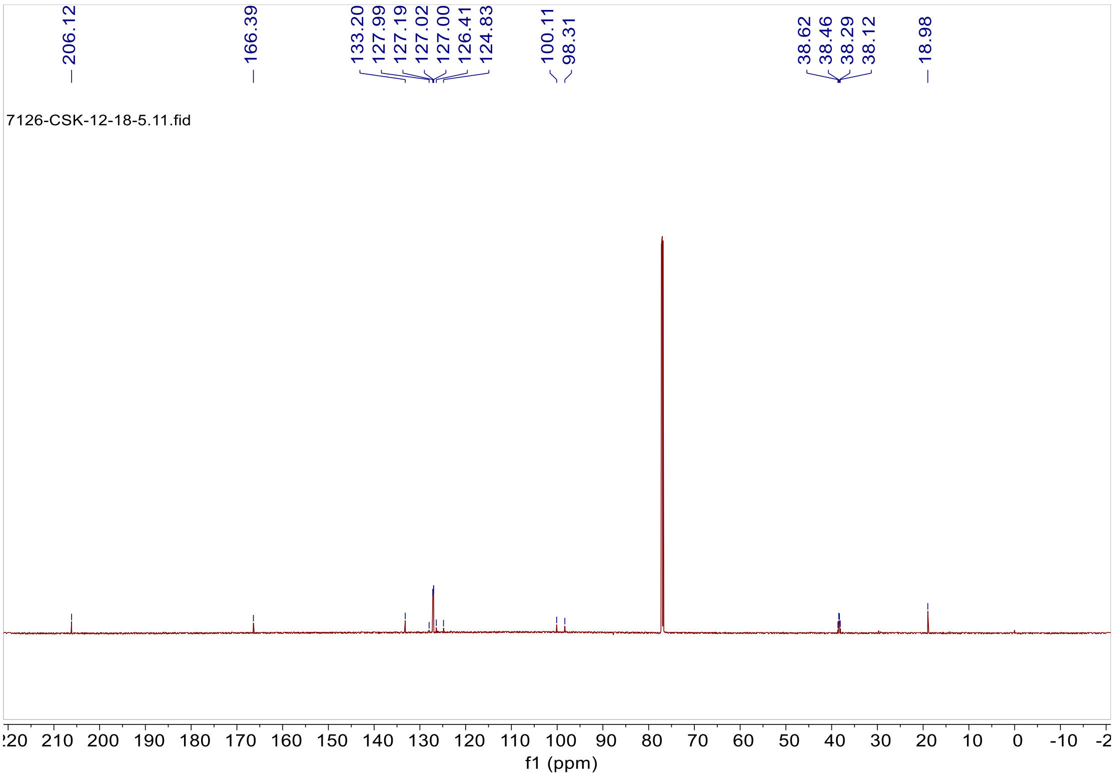

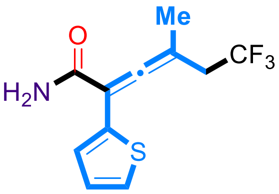

4l

^{19}F NMR (376 MHz, $CDCl_3$)

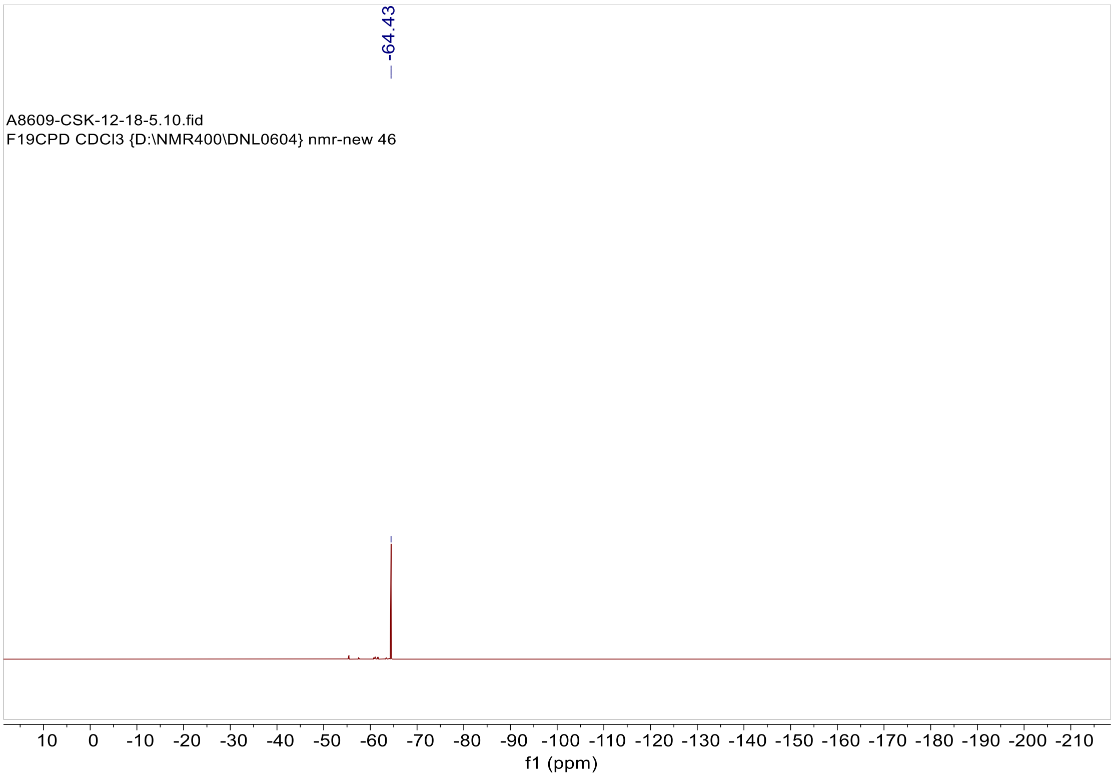

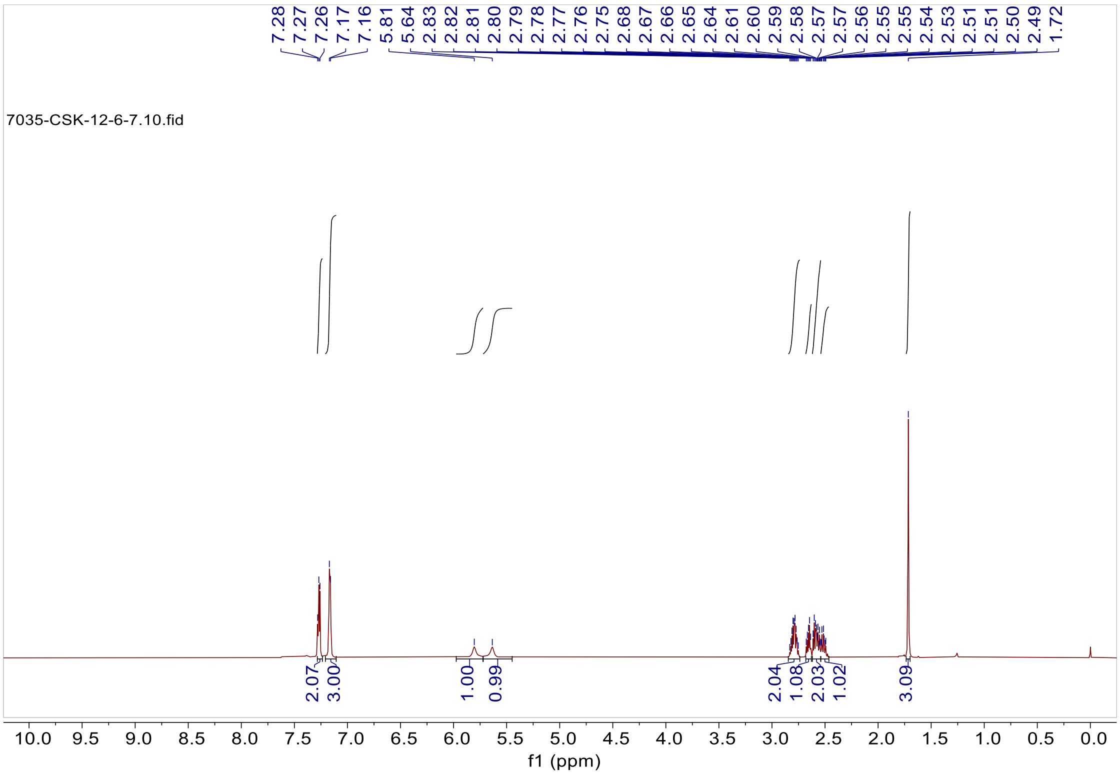

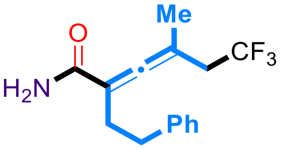

4m

^{1}H NMR (700 MHz, $CDCl_3$)

^{13}C NMR (176 MHz, $CDCl_3$)

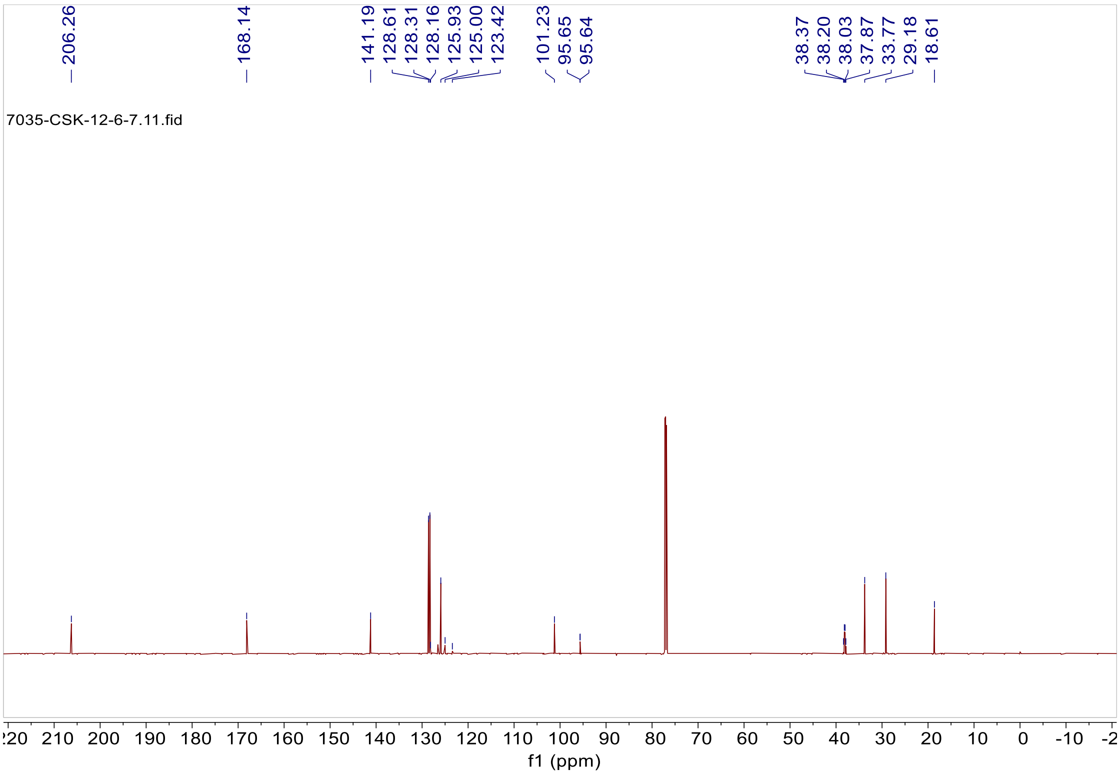

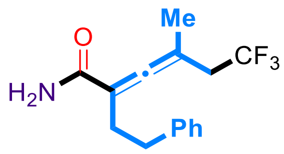

4m ^{19}F NMR (376 MHz, $CDCl_3$)

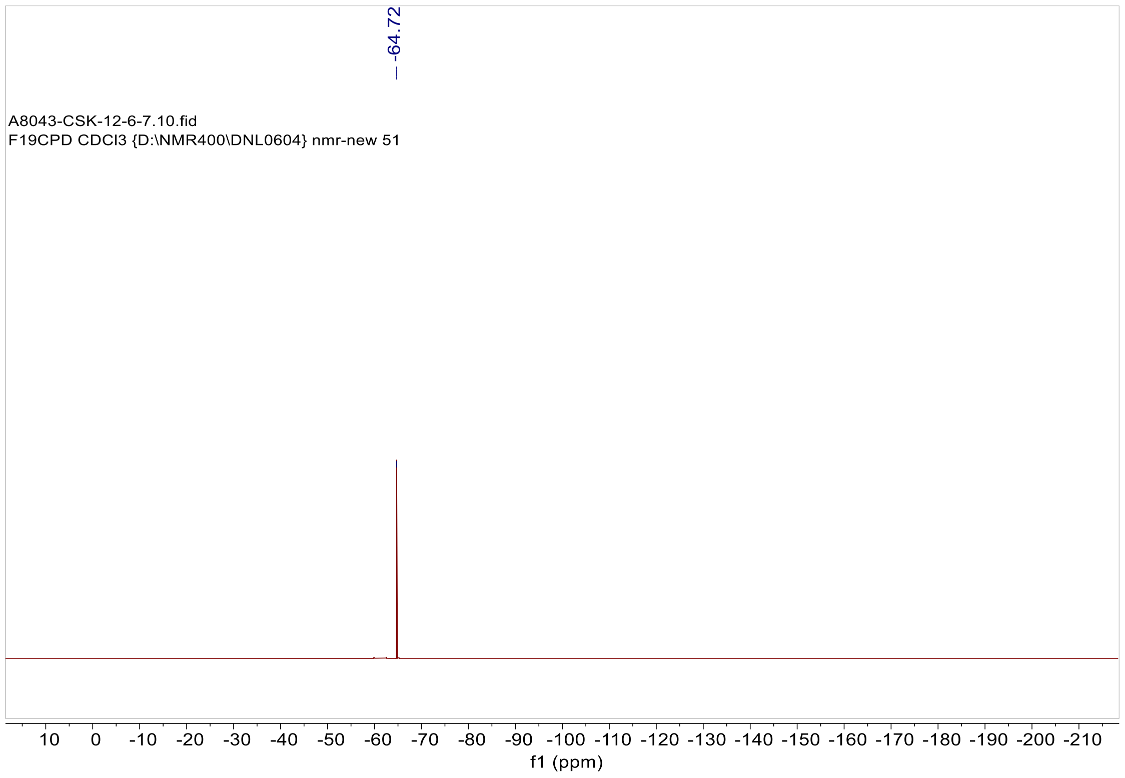

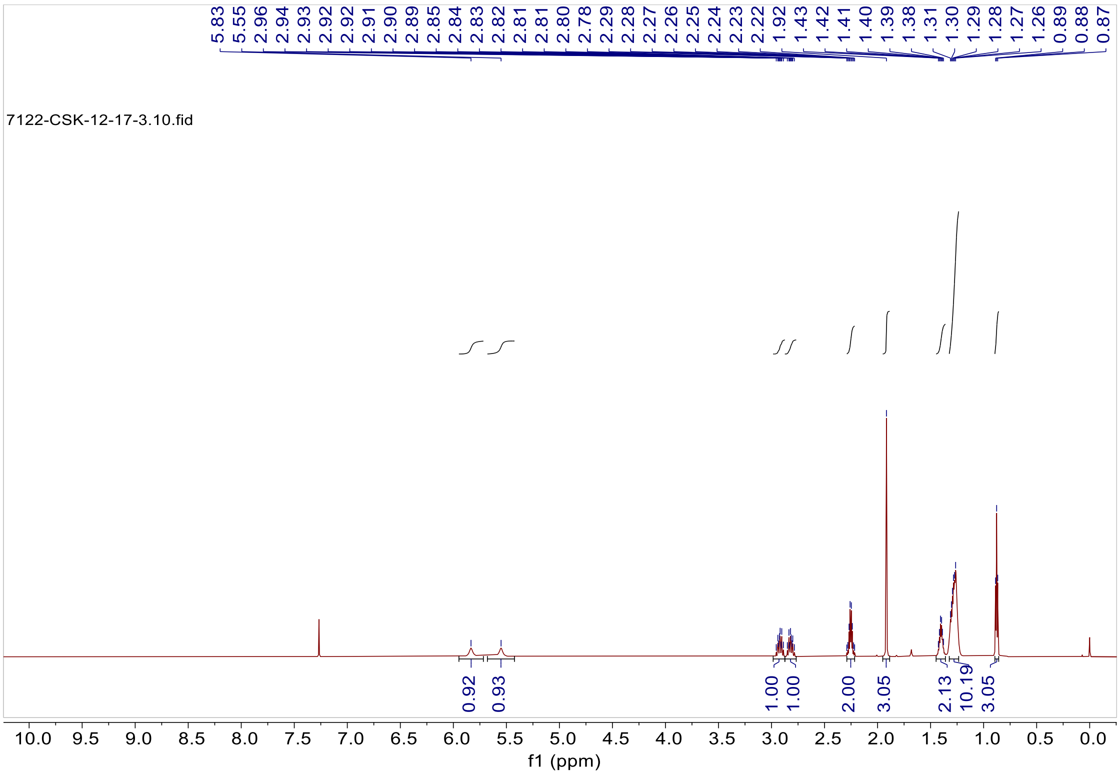

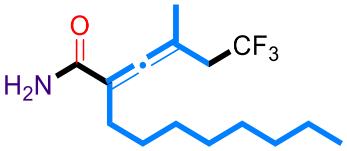

4n

^{1}H NMR (700 MHz, $CDCl_3$)
^{13}C NMR (176 MHz, $CDCl_3$)

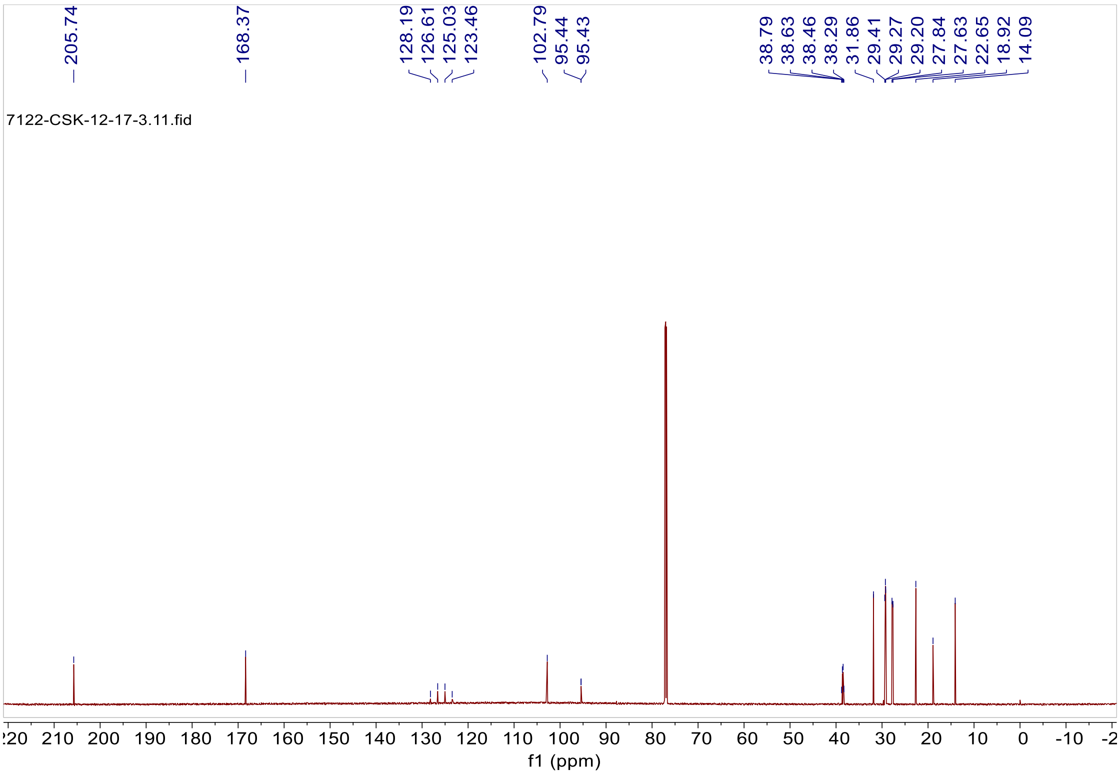

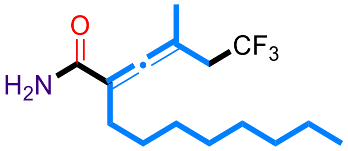

4n

^{19}F NMR (376 MHz, $CDCl_3$)

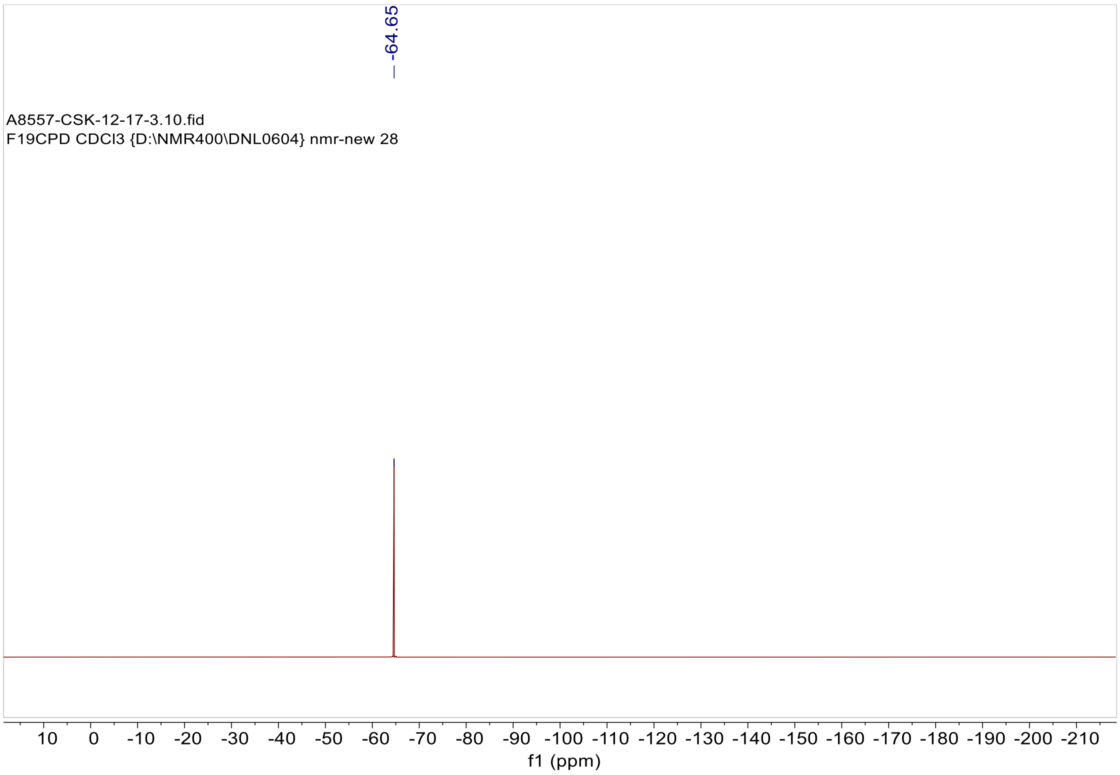

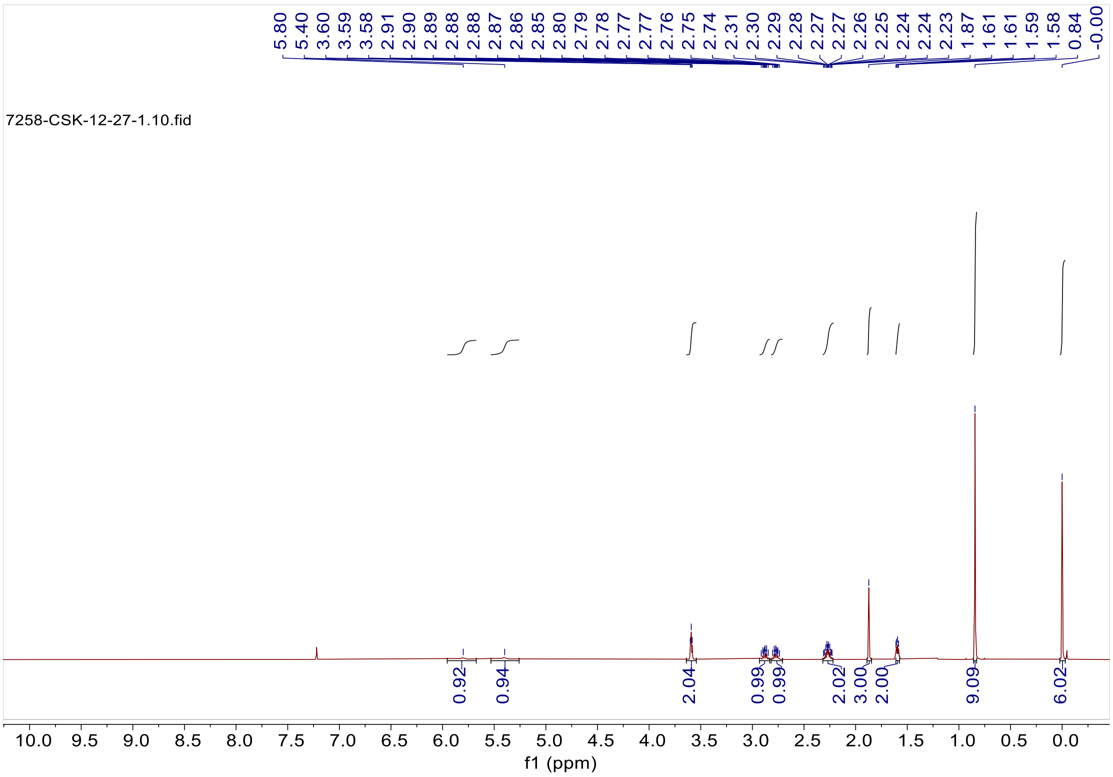

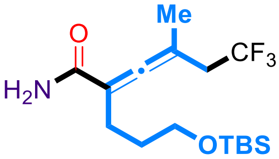

4o

^{1}H NMR (700 MHz, $CDCl_3$)
^{13}C NMR (176 MHz, $CDCl_3$)

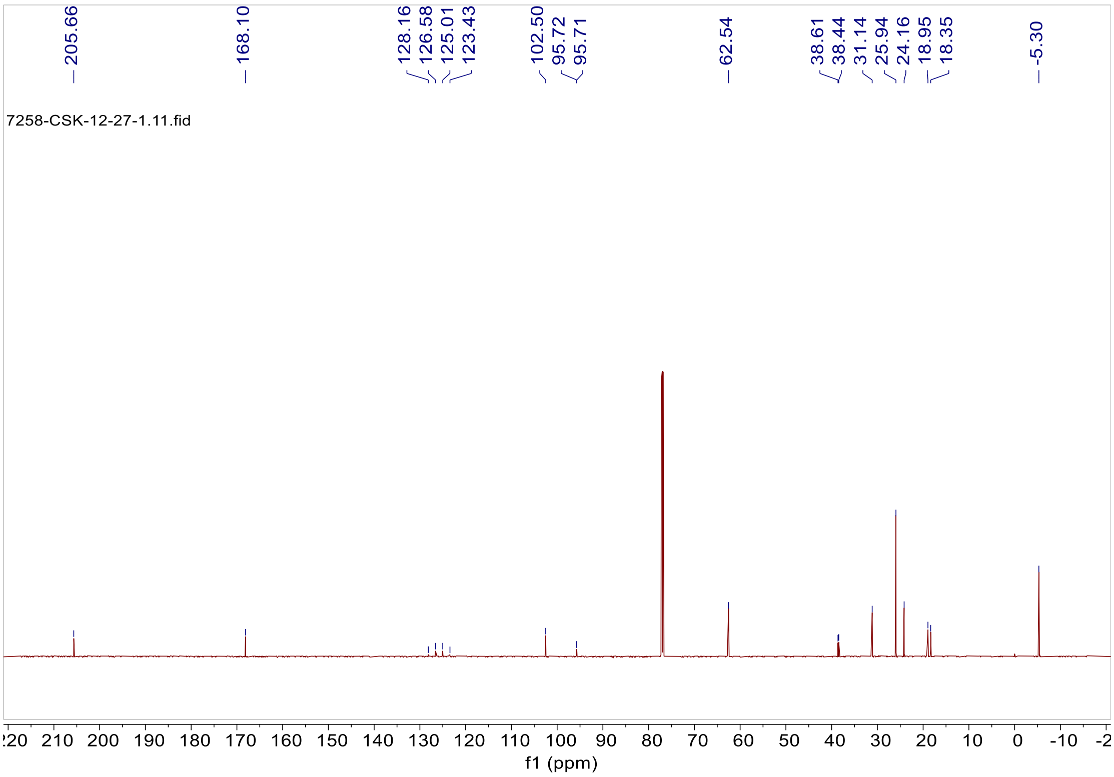

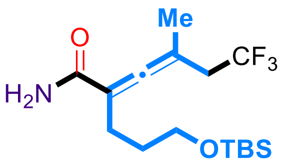

4o

^{19}F NMR (376 MHz, $CDCl_3$)

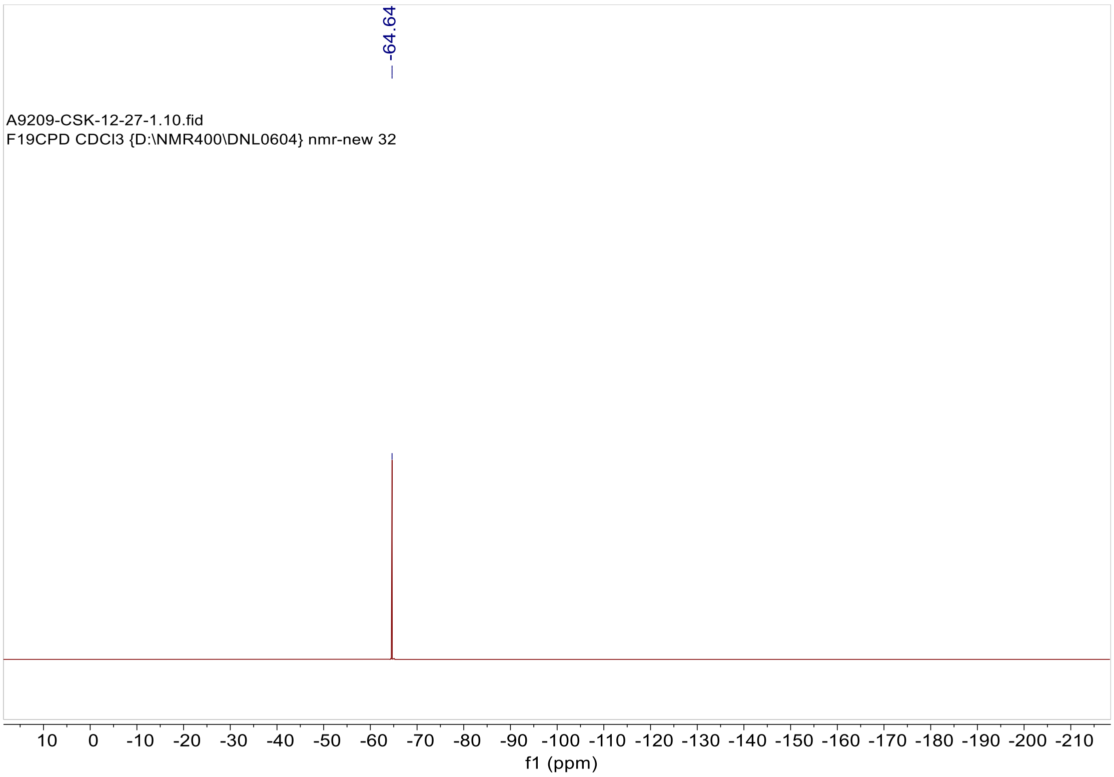

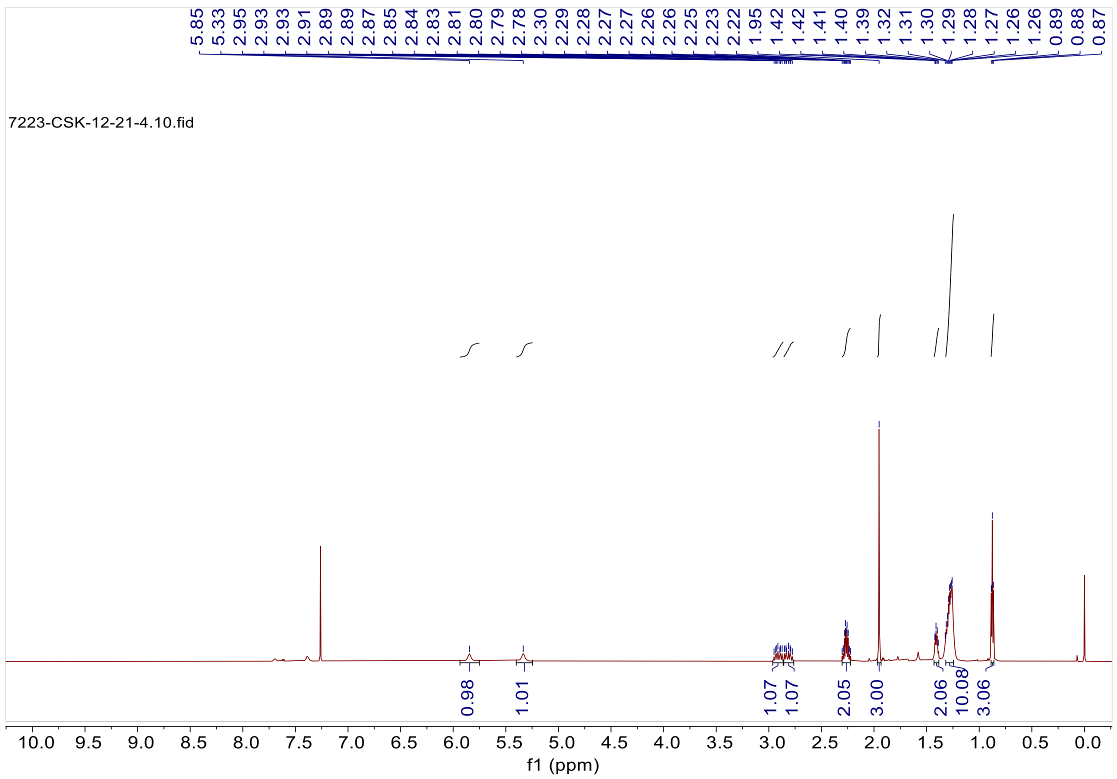

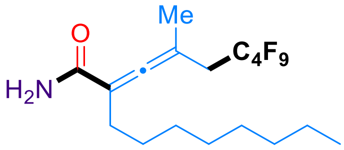

4p

^1H NMR (700 MHz, $CDCl_3$)
^{13}C NMR (176 MHz, $CDCl_3$)

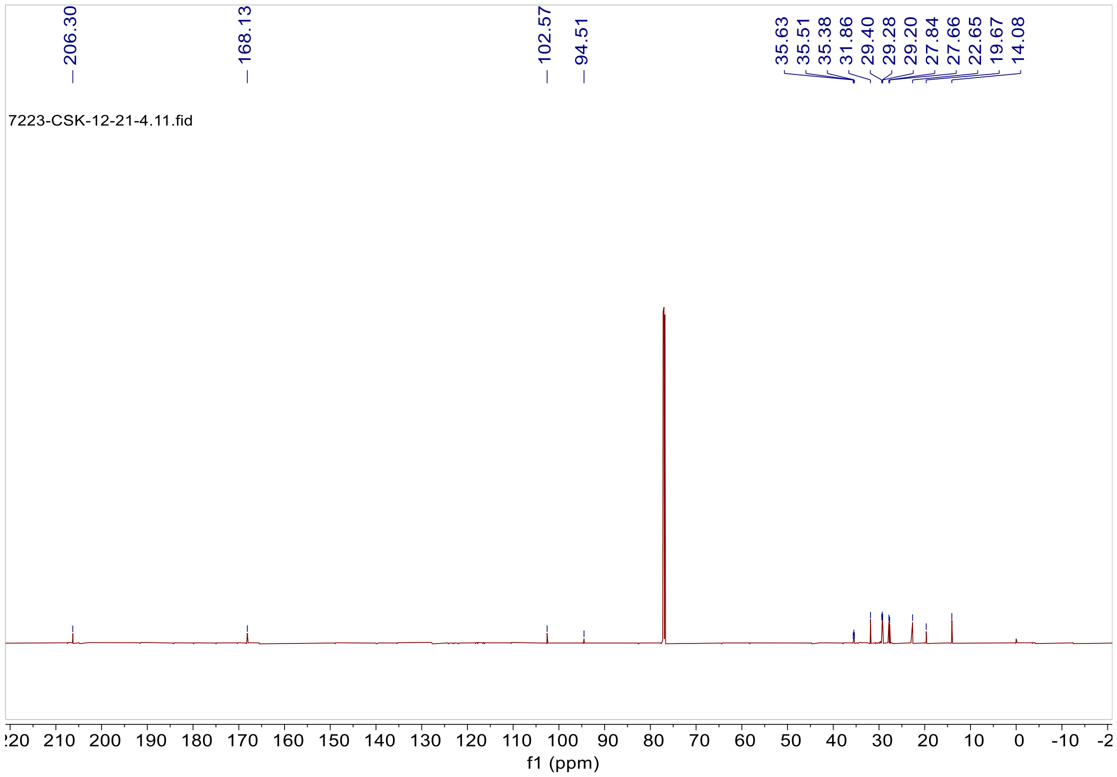

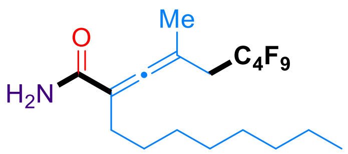

4p ^{19}F NMR (376 MHz, $CDCl_3$)

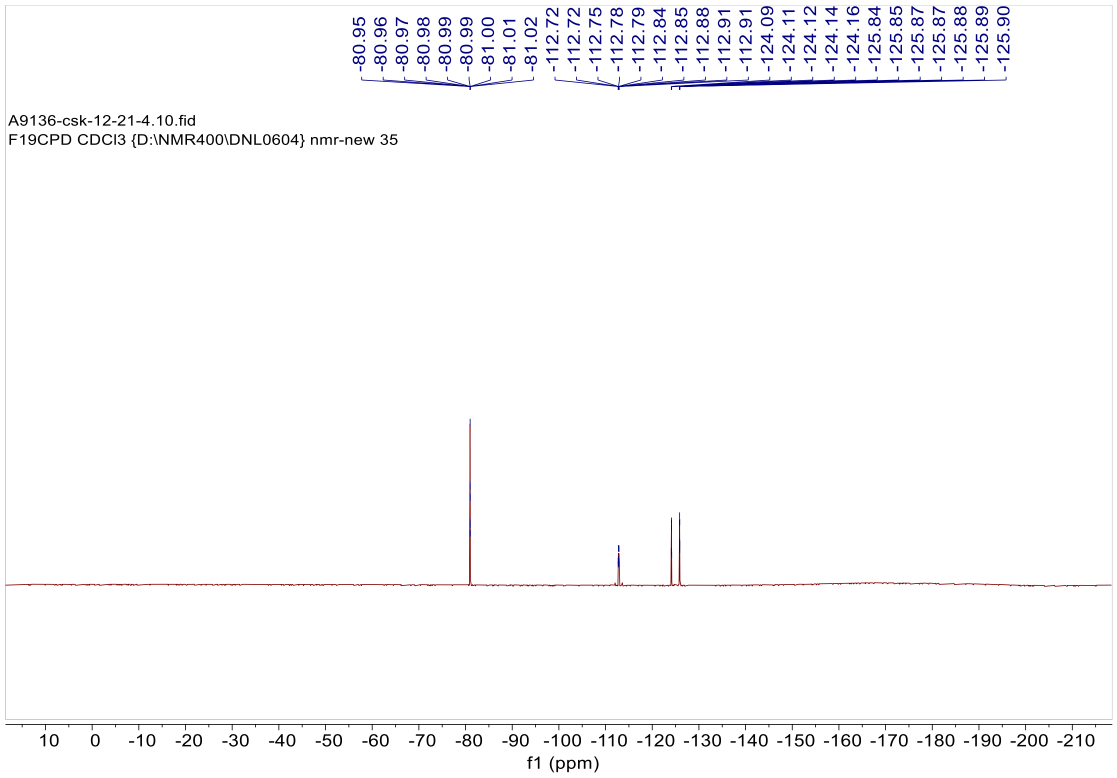

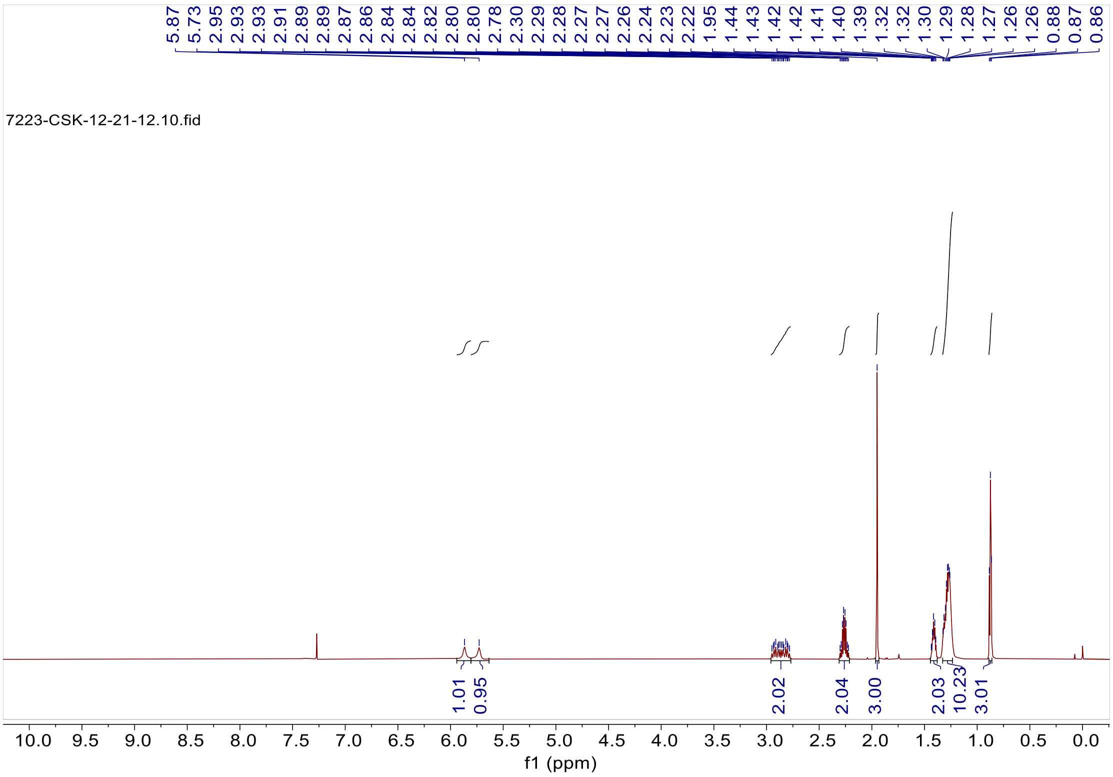

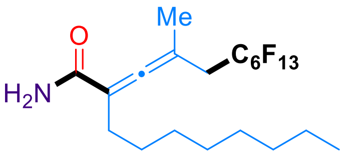

4q

^{1}H NMR (700 MHz, $CDCl_3$)

^{13}C NMR (176 MHz, $CDCl_3$)

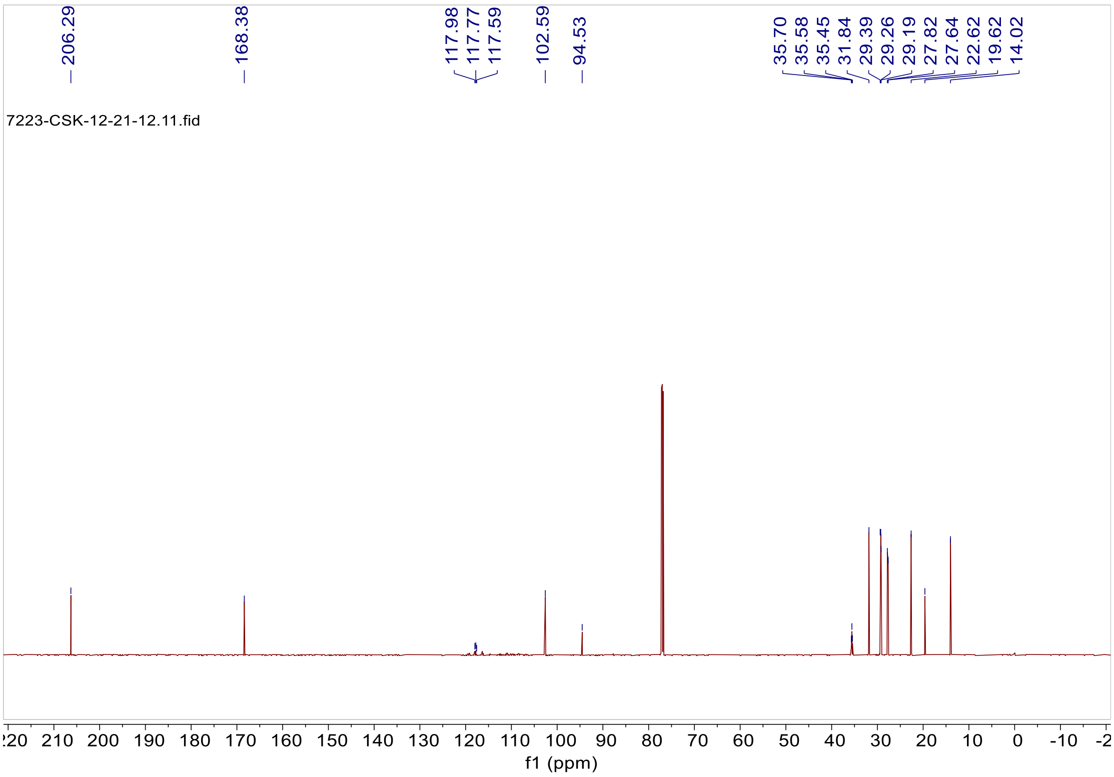

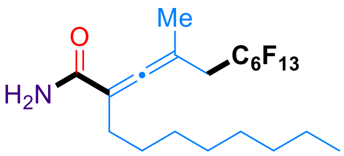

4q ^{19}F NMR (376 MHz, $CDCl_3$)

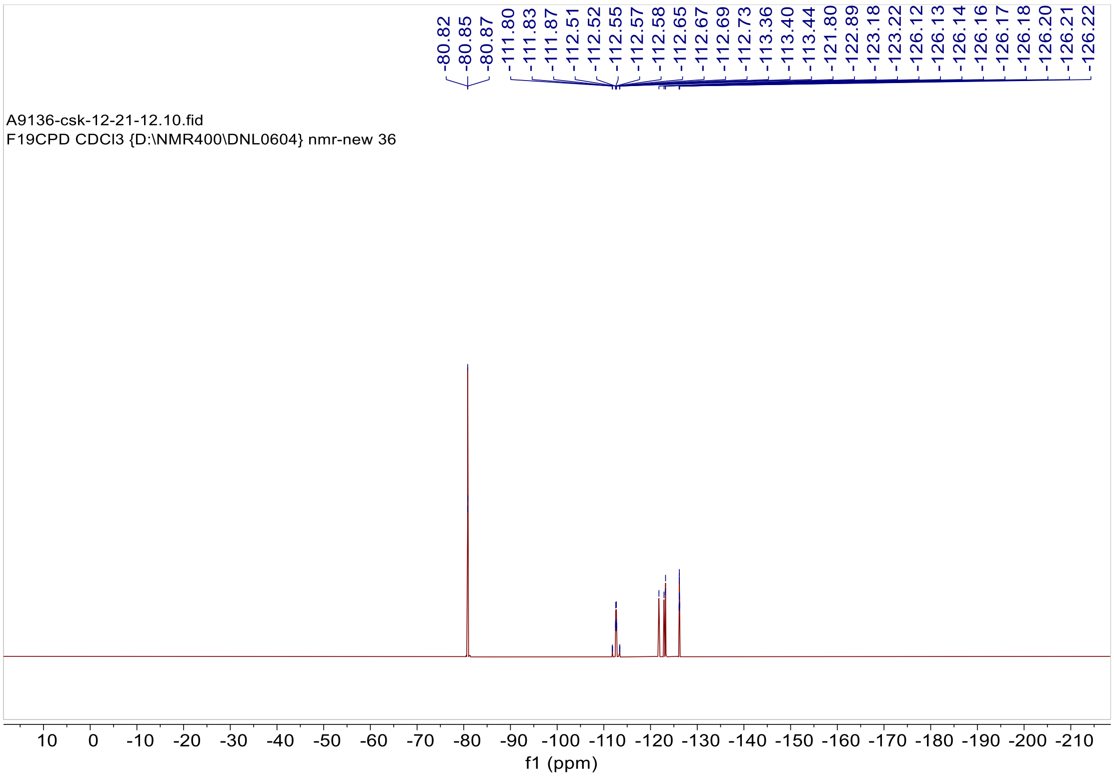

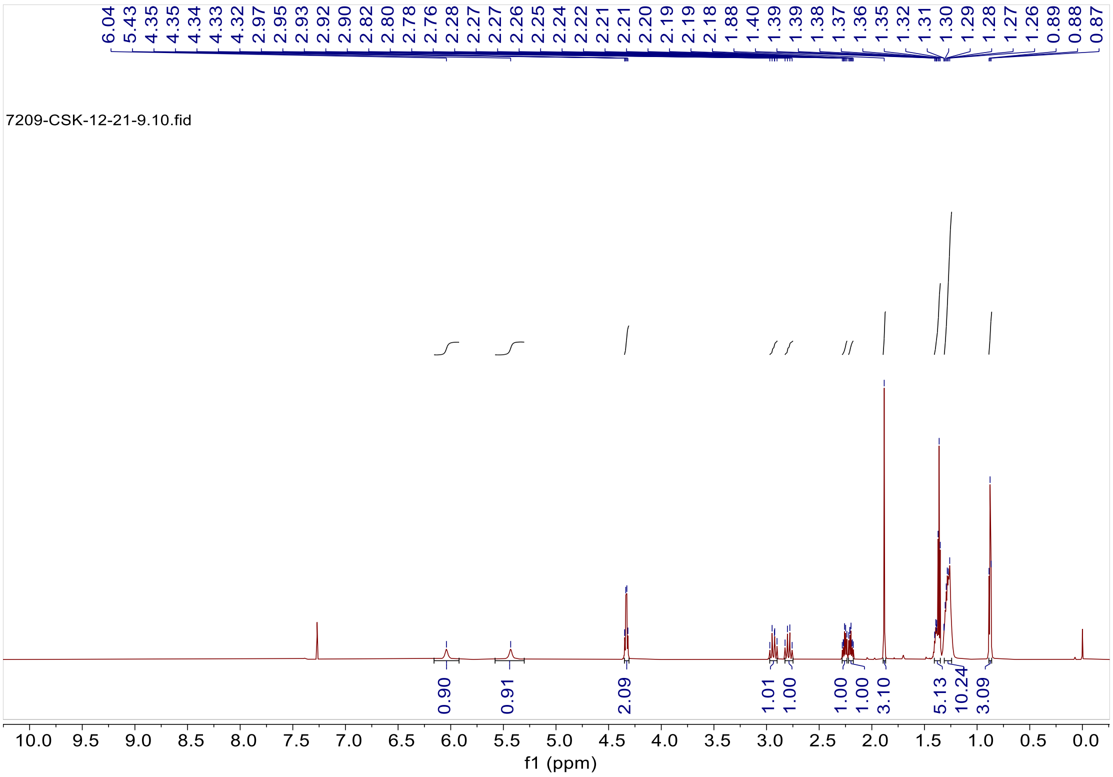

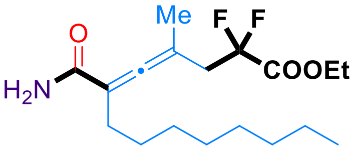

4r

^{1}H NMR (700 MHz, CDCl$_3$)

^{13}C NMR (176 MHz, CDCl$_3$)

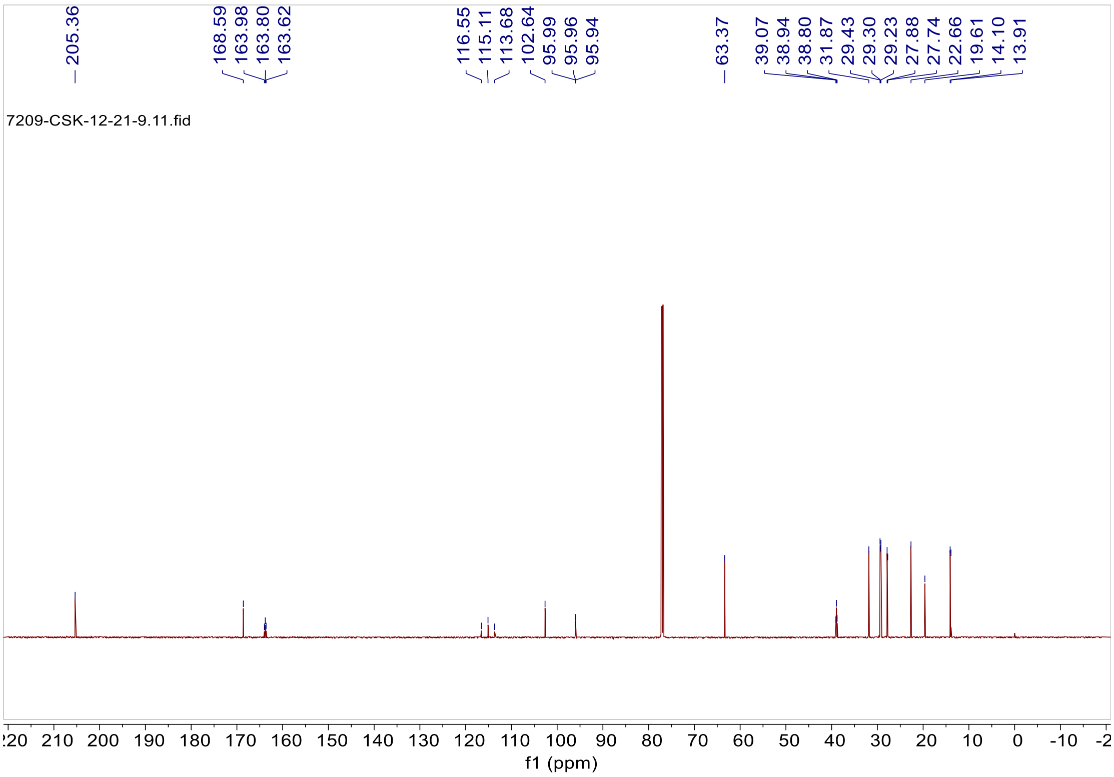

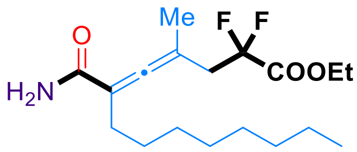

4r

^{19}F NMR (376 MHz, $CDCl_3$)

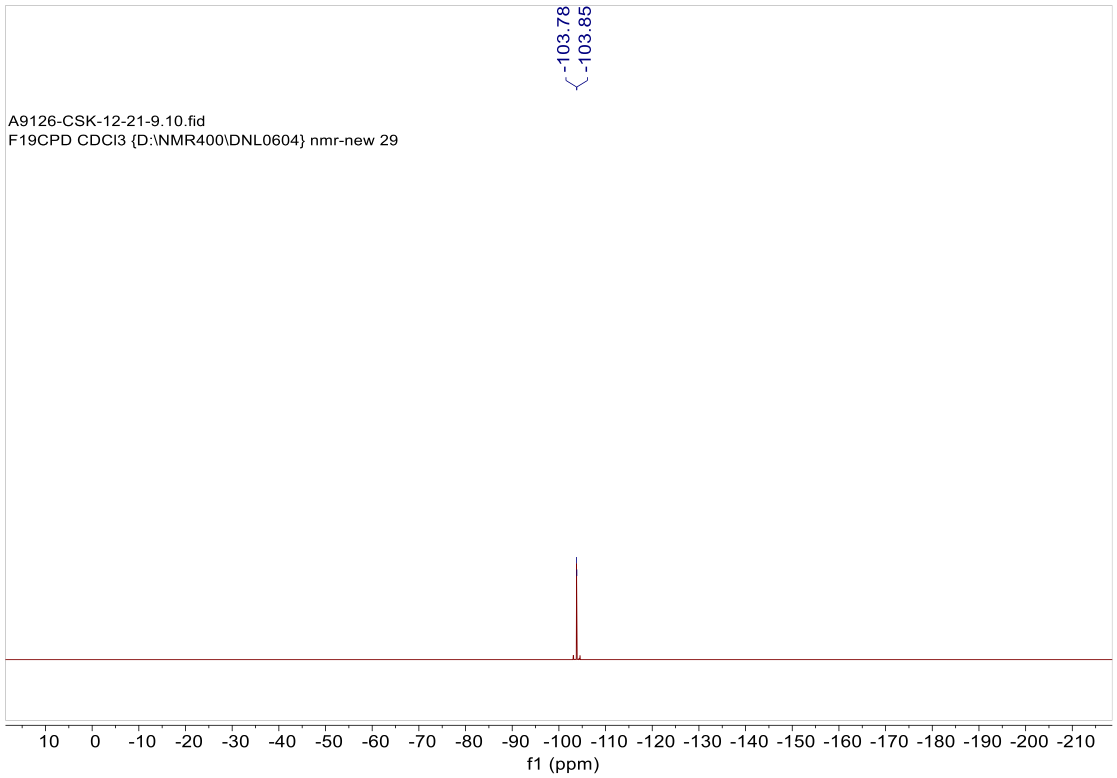

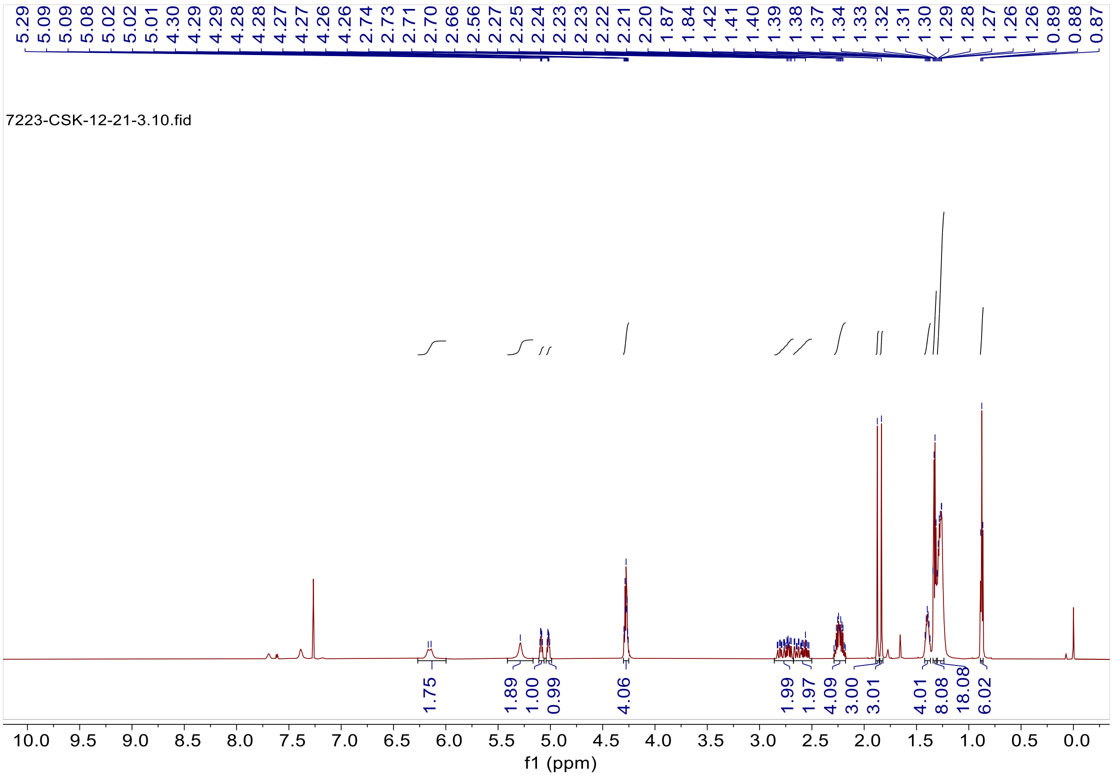

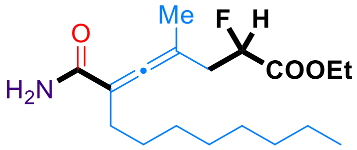

4s

^{1}H NMR (700 MHz, $CDCl_3$)
^{13}C NMR (176 MHz, $CDCl_3$)

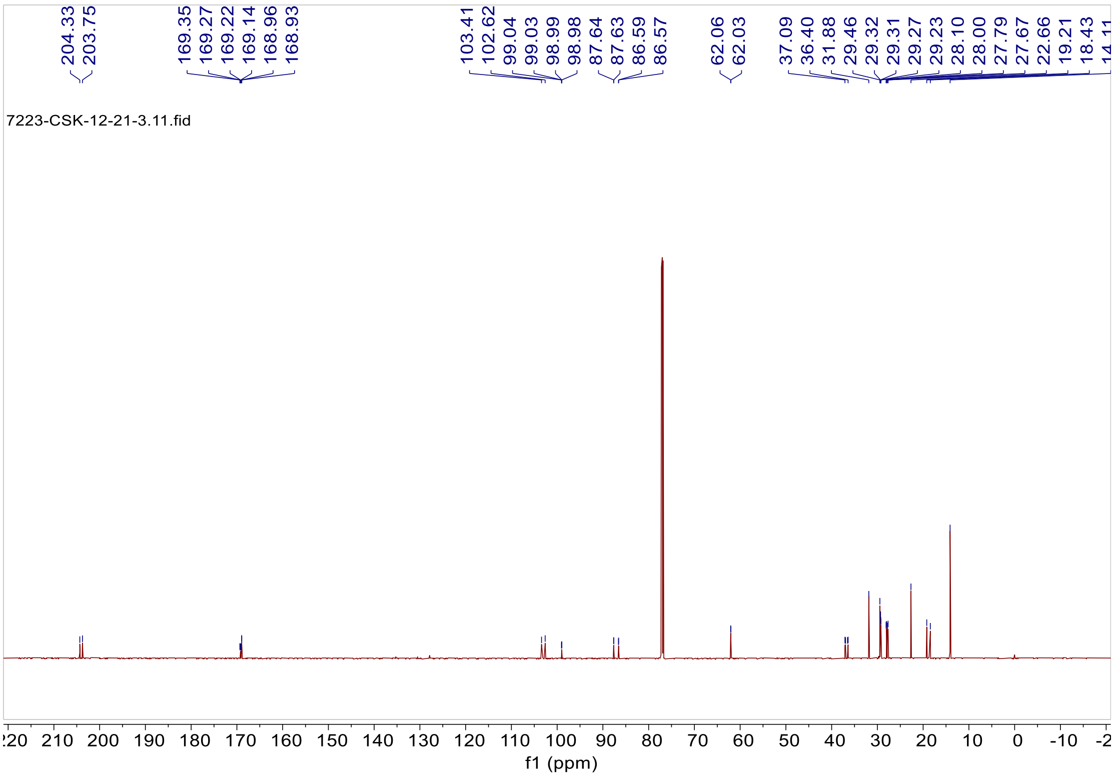

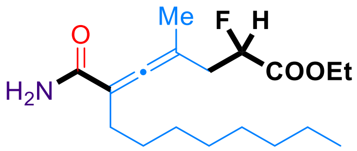

4s

^{19}F NMR (376 MHz, $CDCl_3$)

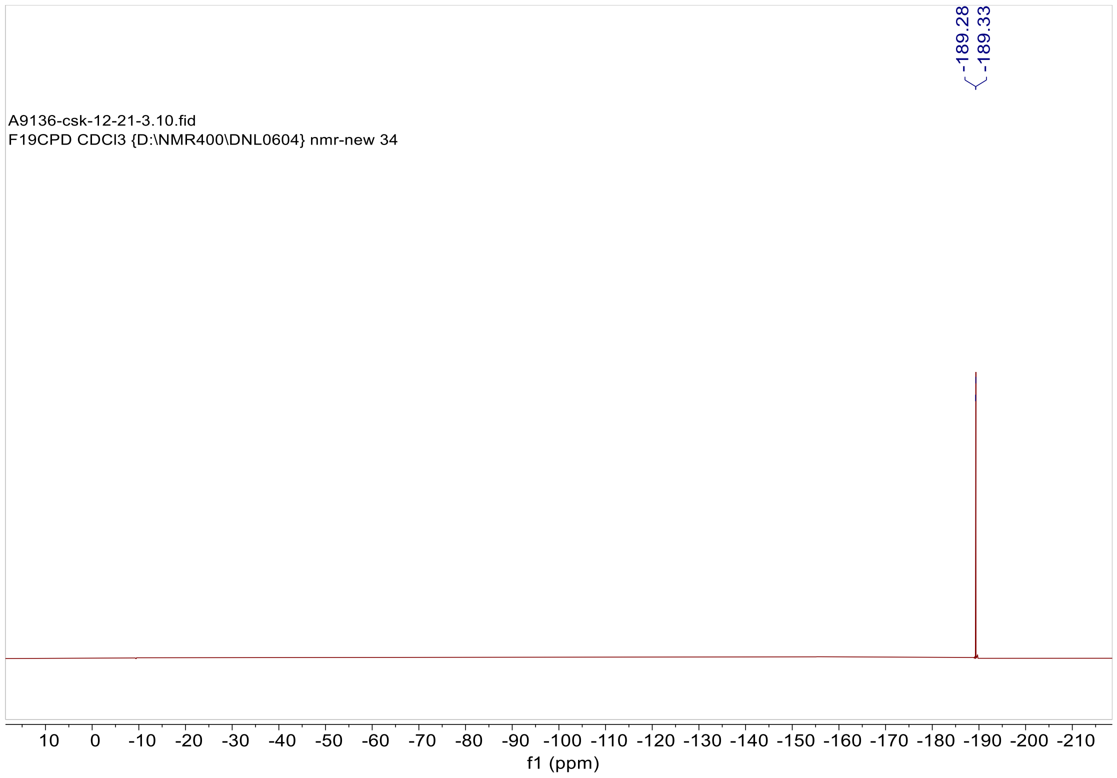

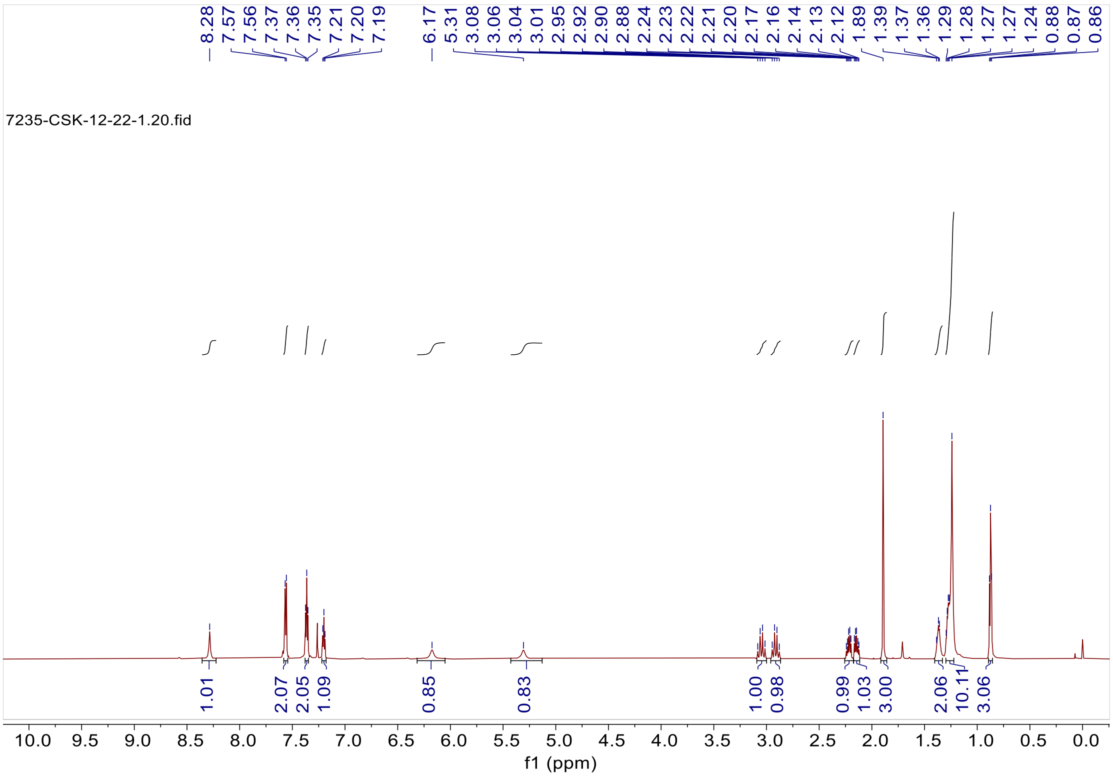

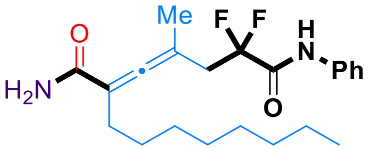

4t

^{1}H NMR (700 MHz, $CDCl_3$)
^{13}C NMR (176 MHz, $CDCl_3$)

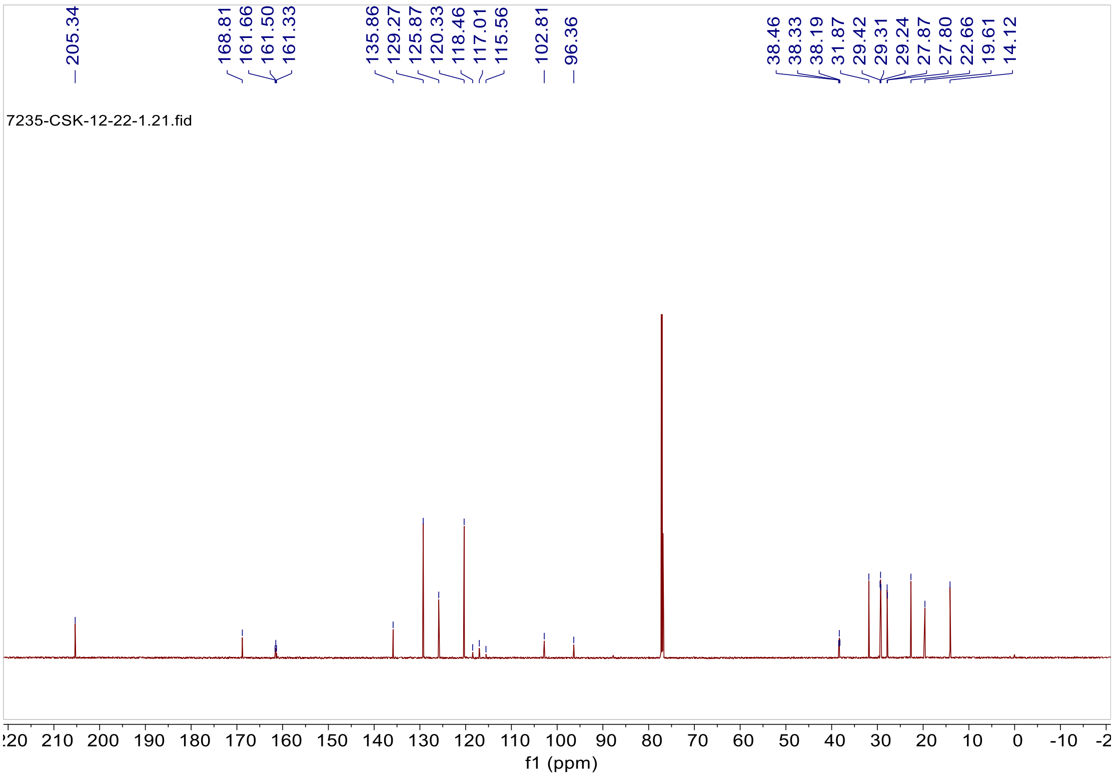

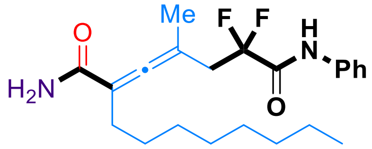

4t

^{19}F NMR (376 MHz, $CDCl_3$)

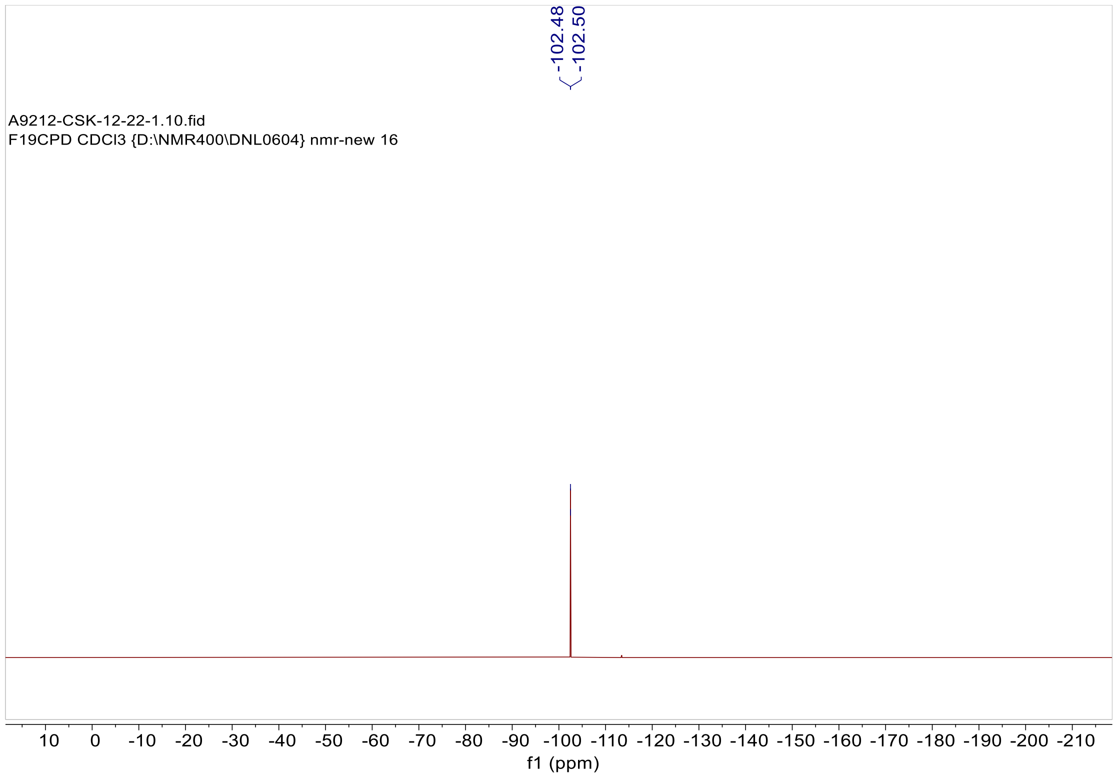

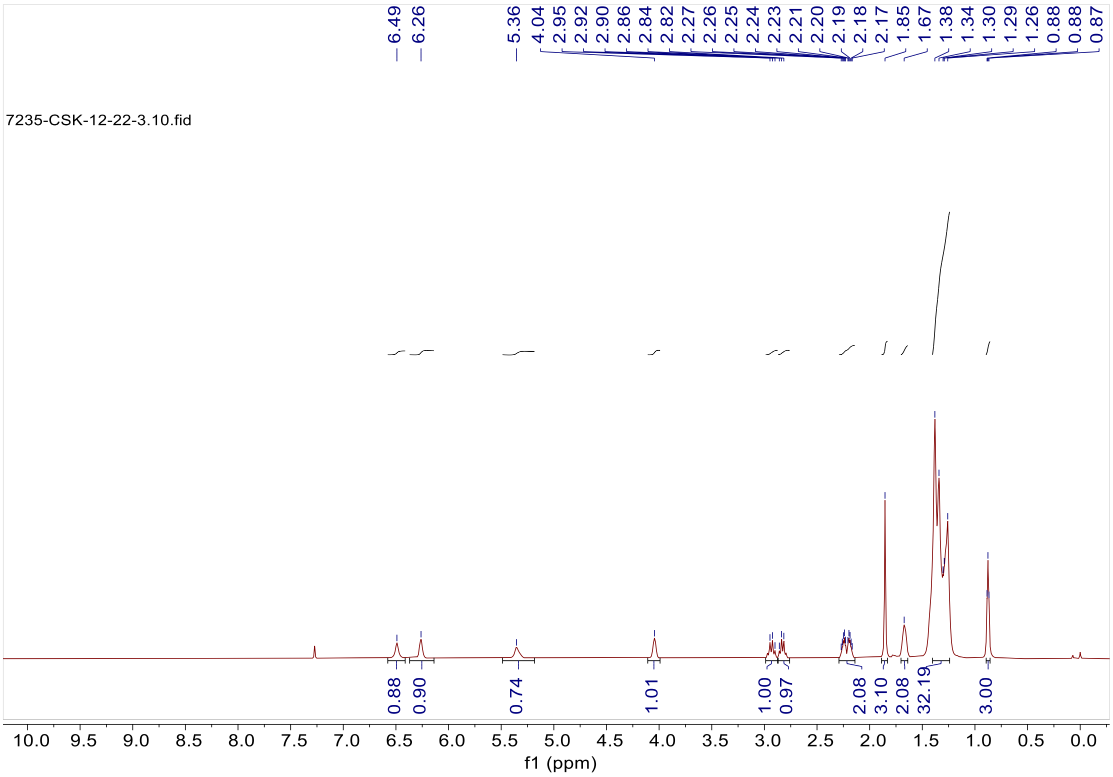

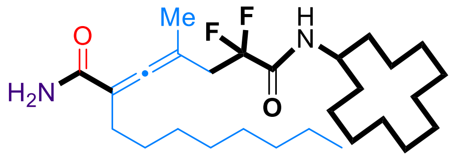

4u

^{1}H NMR (700 MHz, $CDCl_3$)

^{13}C NMR (176 MHz, $CDCl_3$)

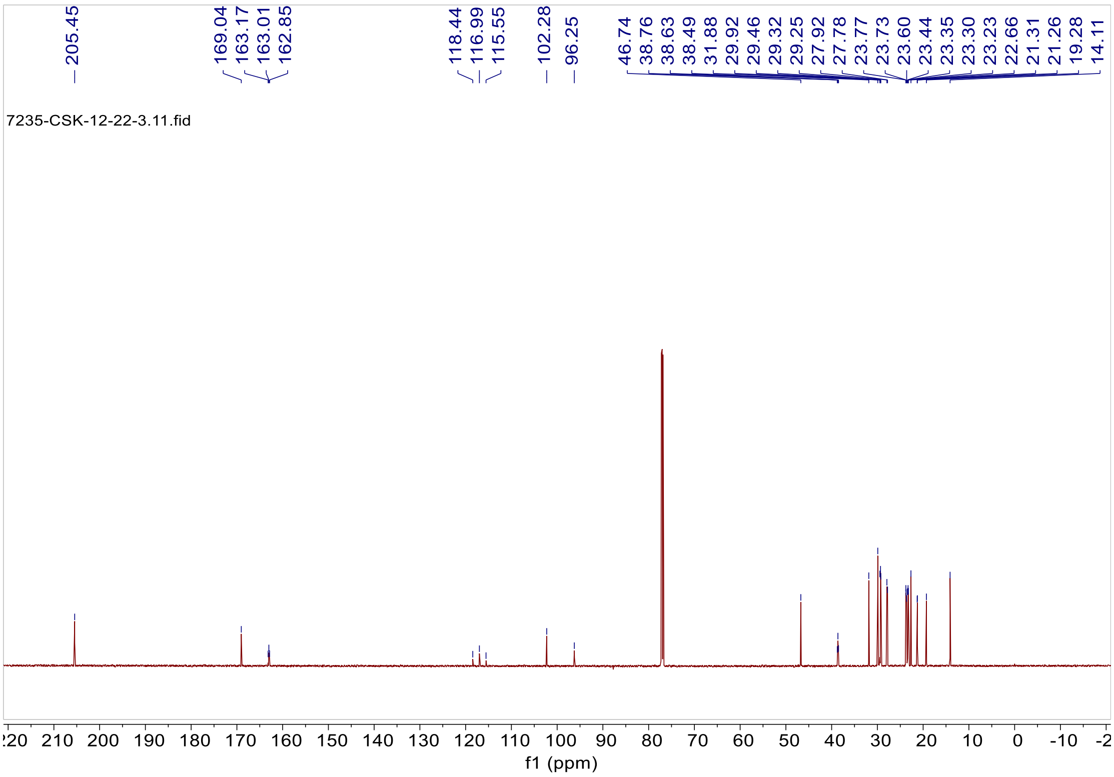

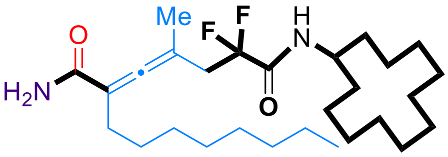

4u

^{19}F NMR (376 MHz, $CDCl_3$)

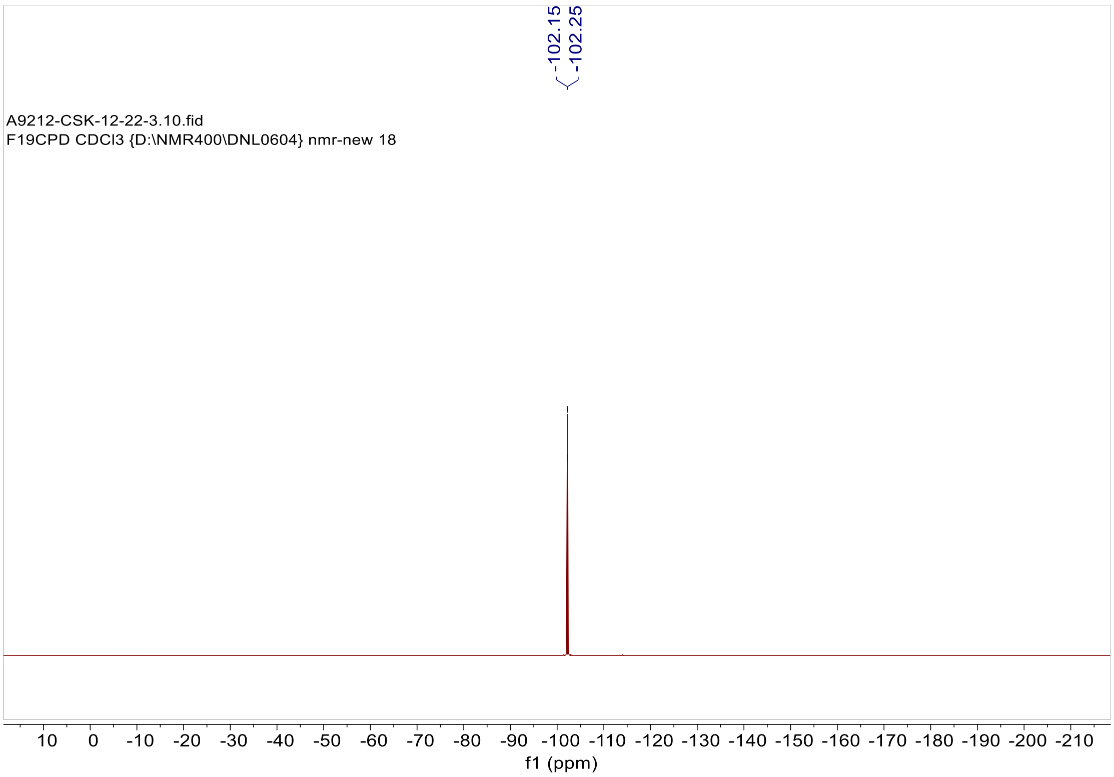

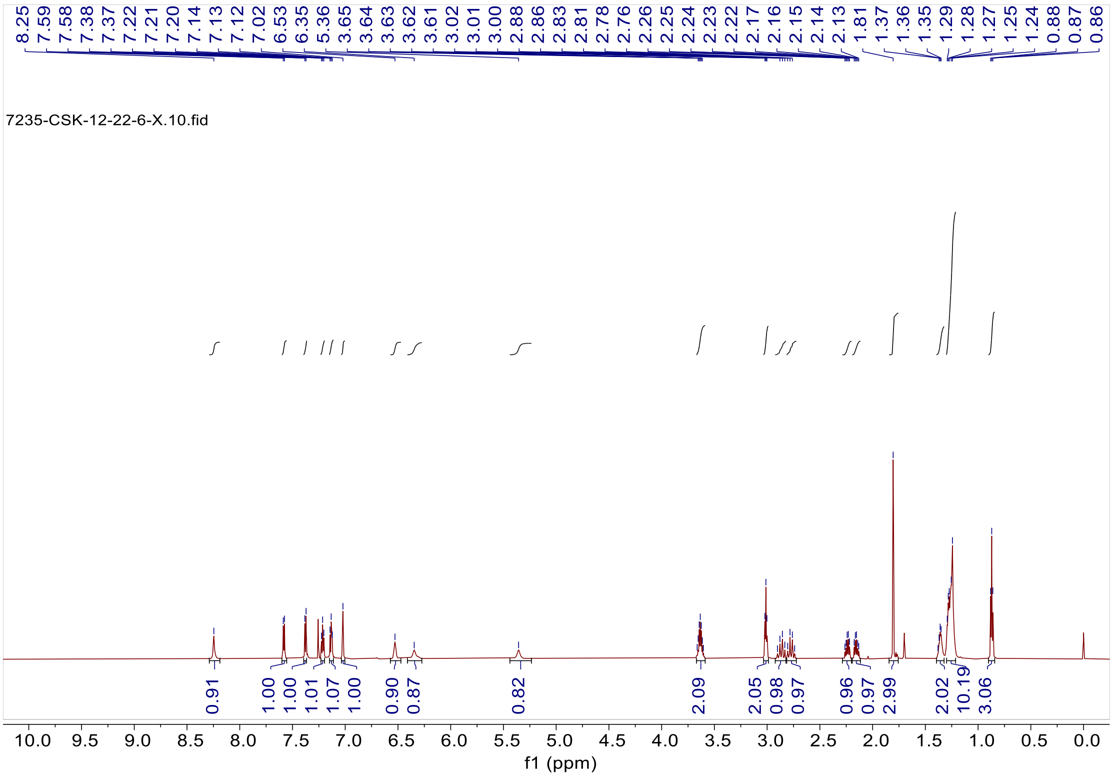

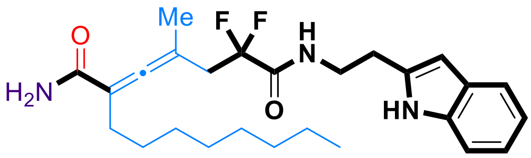

4v

^{1}H NMR (700 MHz, $CDCl_3$)
^{13}C NMR (176 MHz, $CDCl_3$)

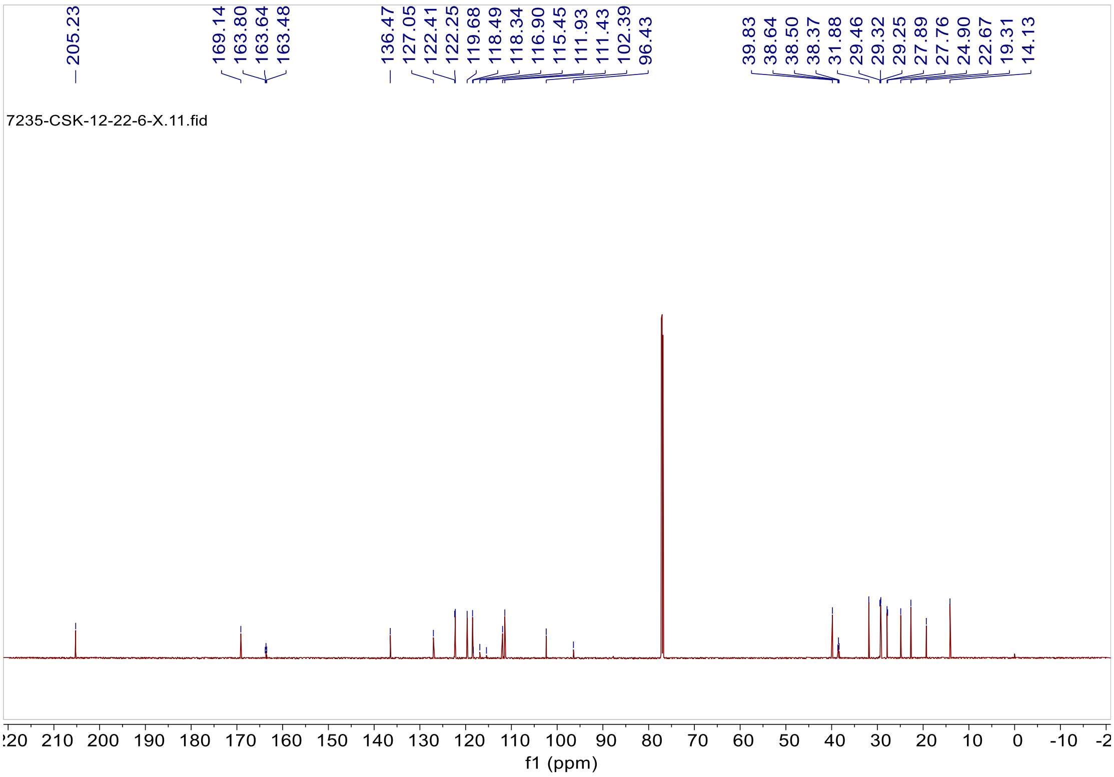

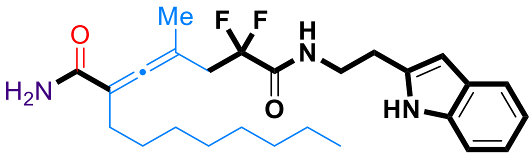

4v ^{19}F NMR (376 MHz, $CDCl_3$)

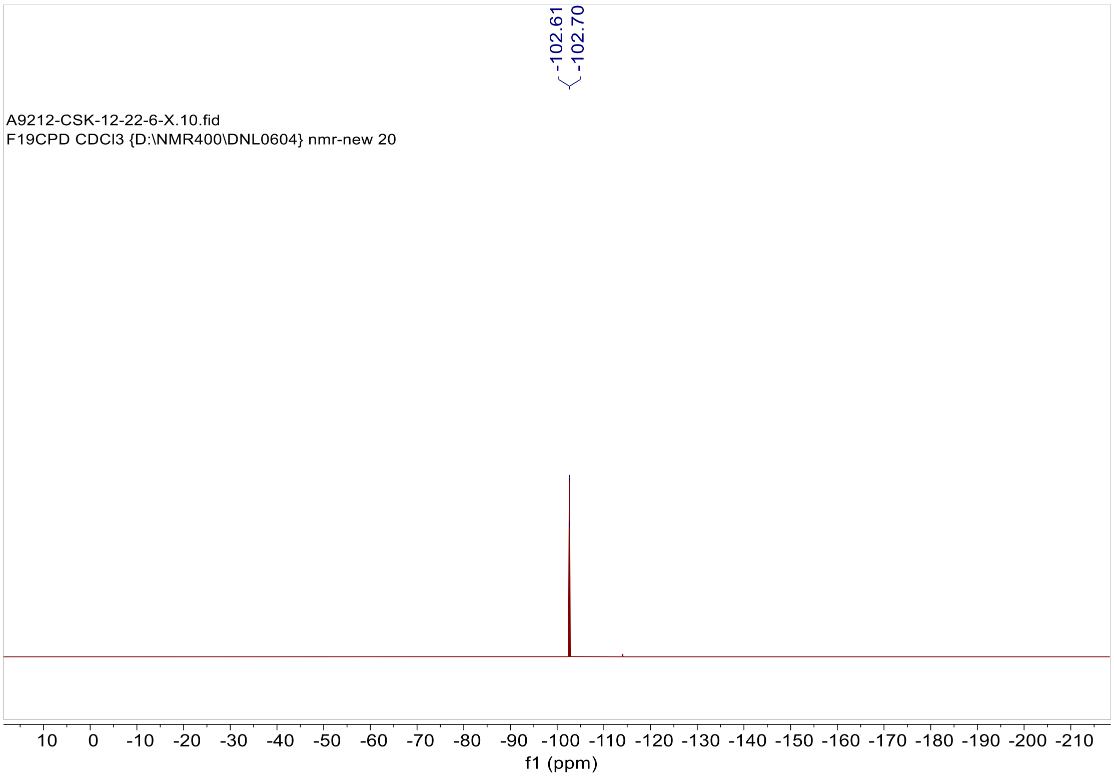

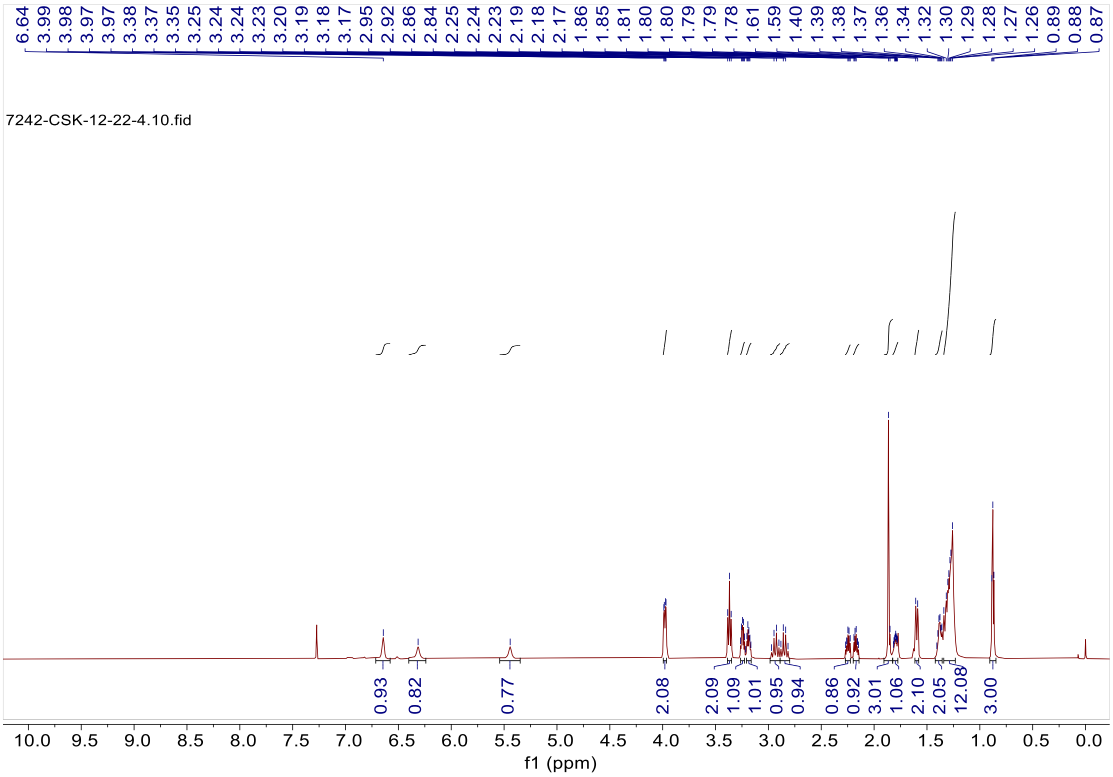

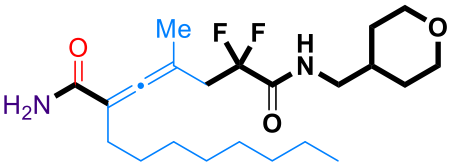

4w

^{1}H NMR (700 MHz, $CDCl_3$)

^{13}C NMR (176 MHz, $CDCl_3$)

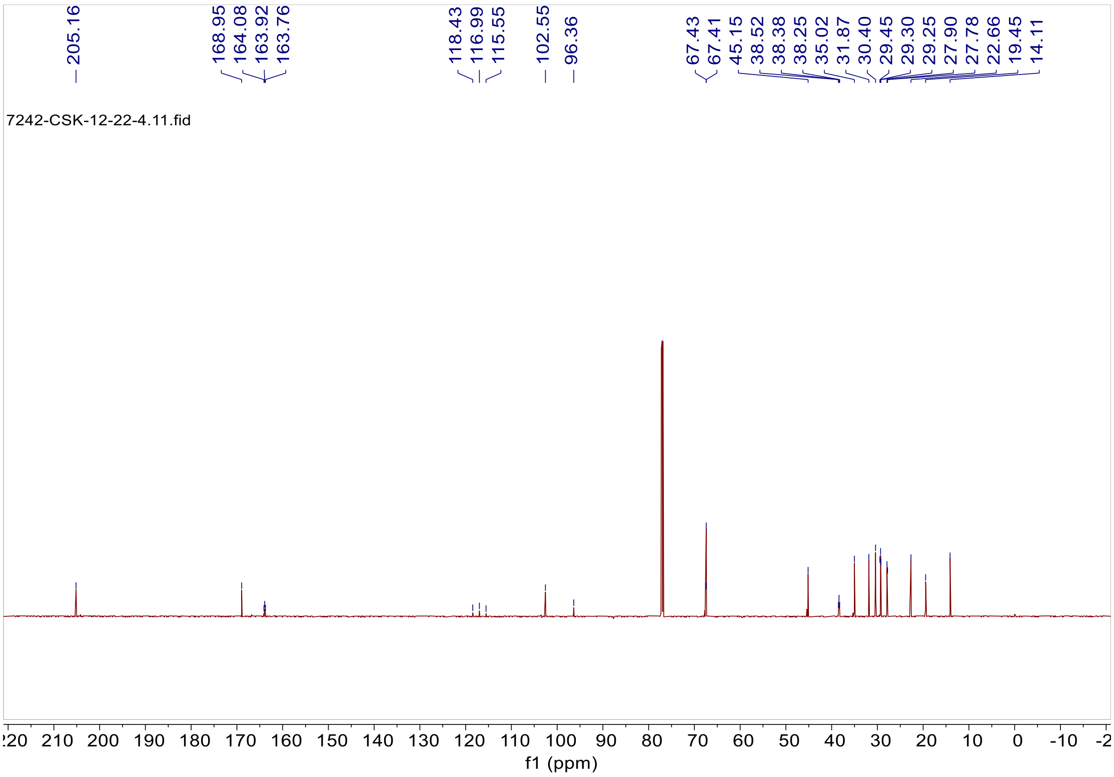

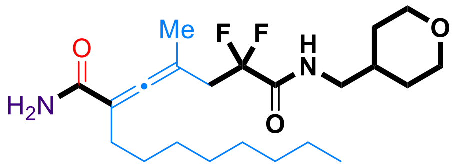

4w

^{19}F NMR (376 MHz, $CDCl_3$)

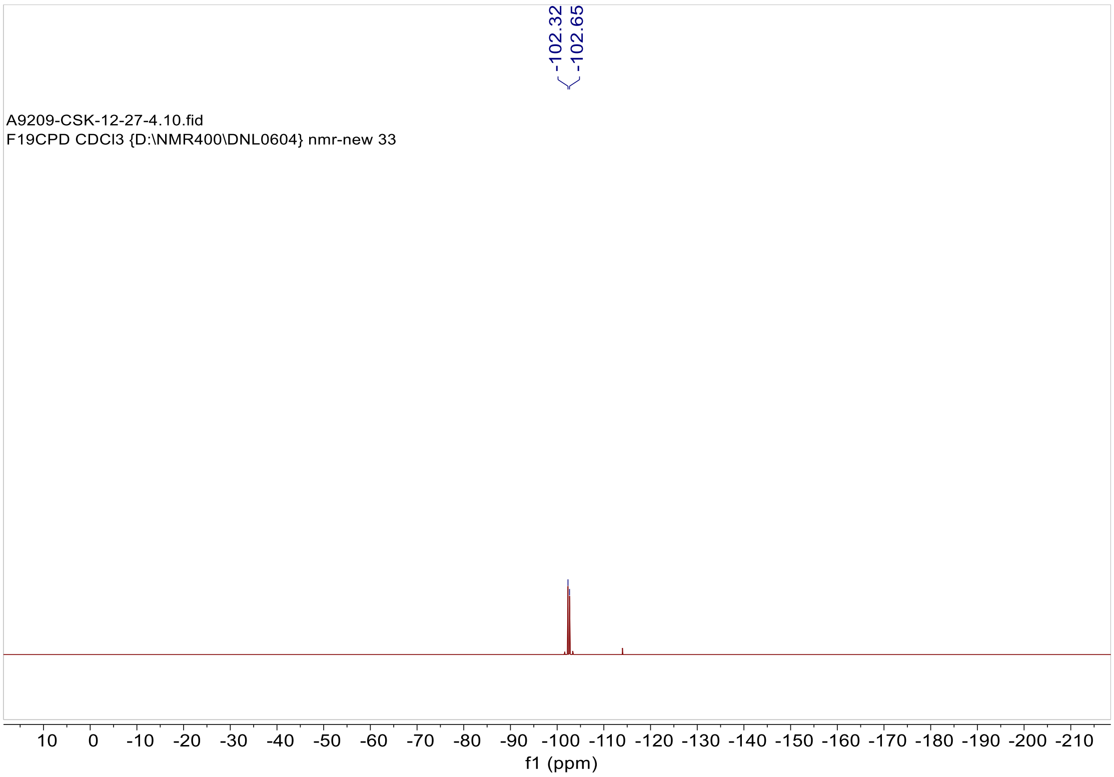

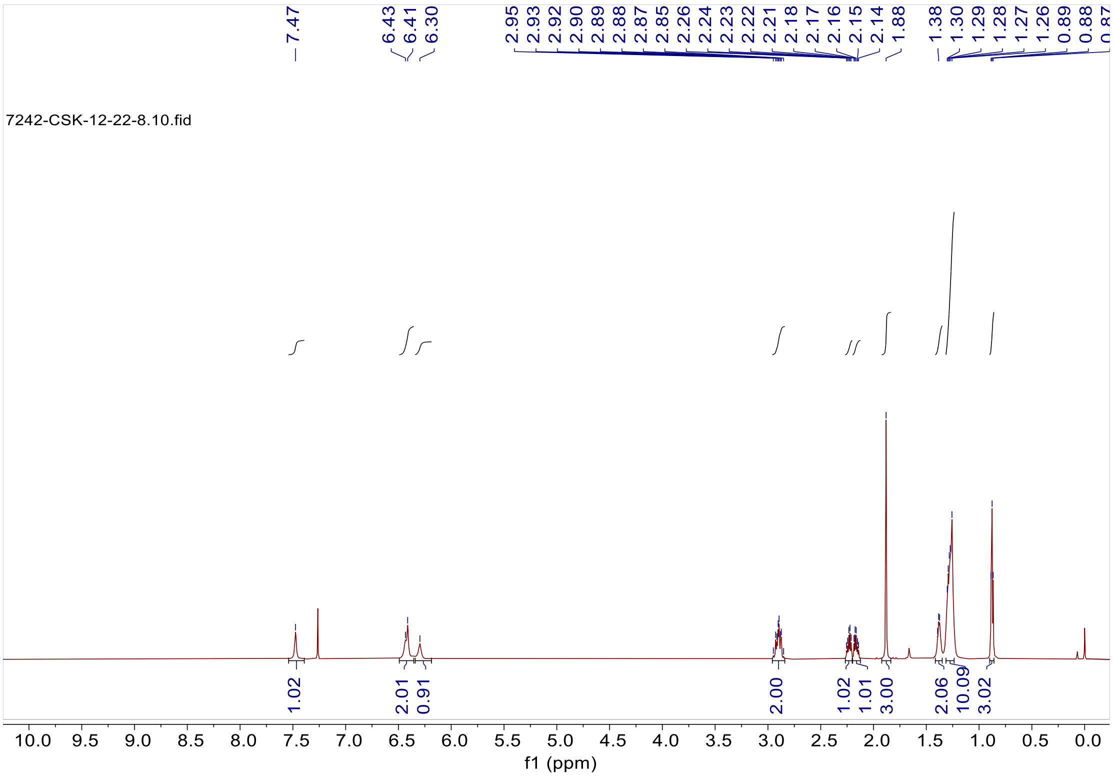

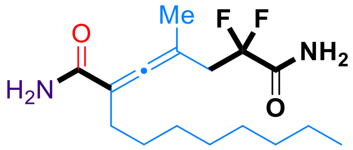

4x

^1H NMR (700 MHz, $CDCl_3$)
^{13}C NMR (176 MHz, $CDCl_3$)

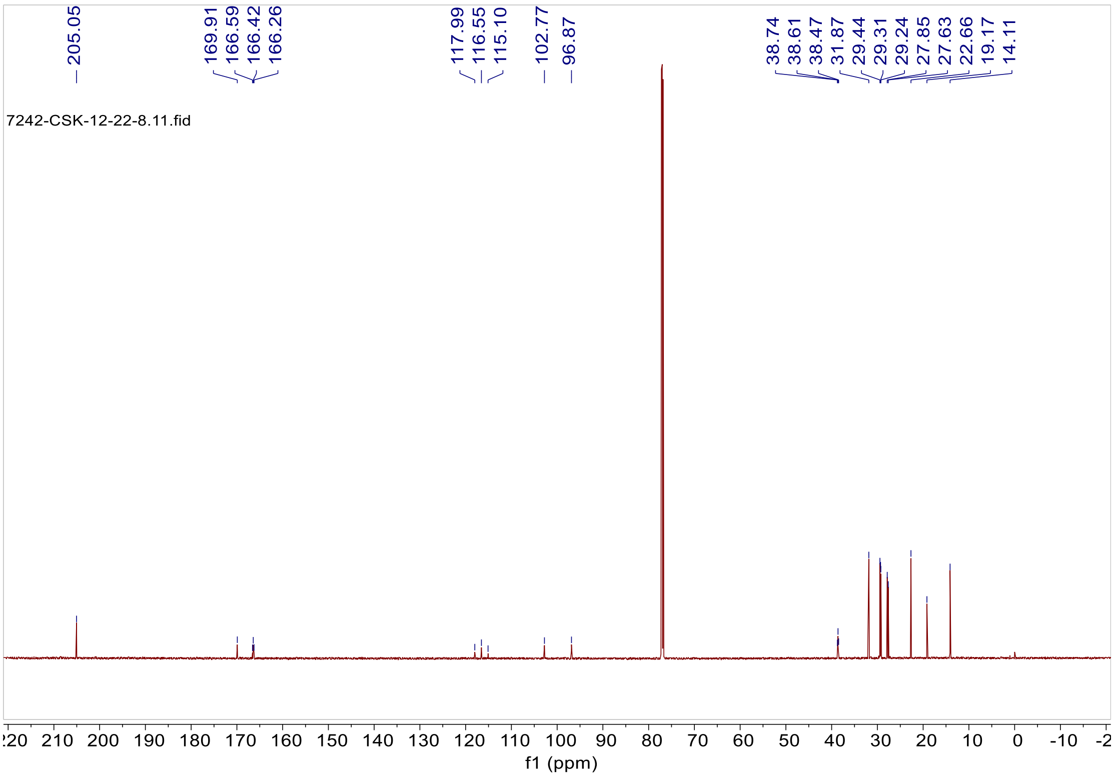

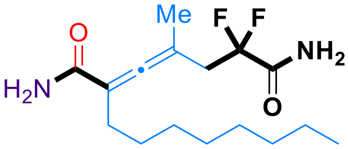

4x ^{19}F NMR (376 MHz, $CDCl_3$)

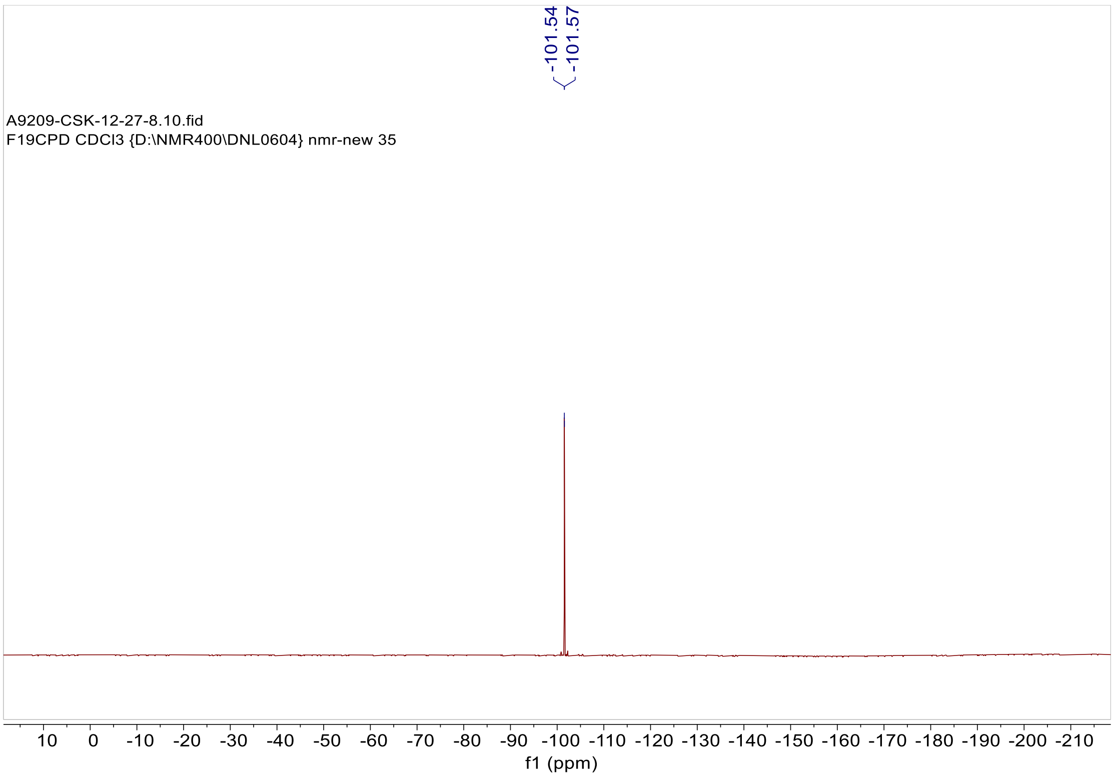

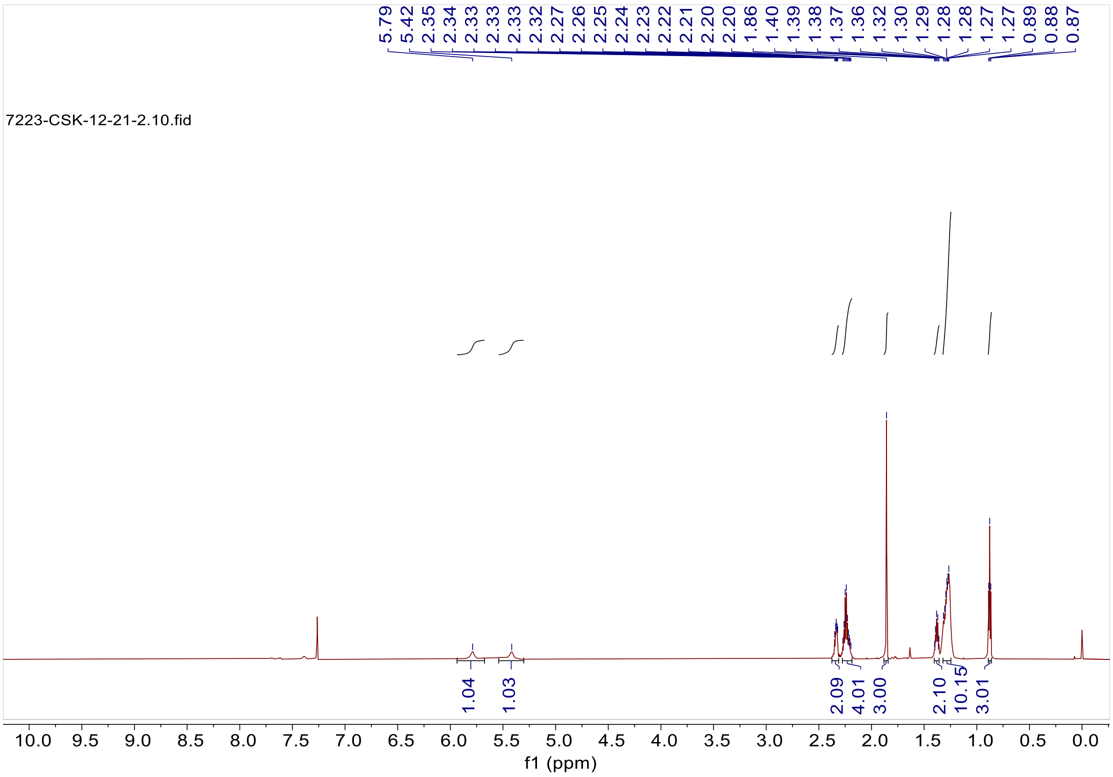

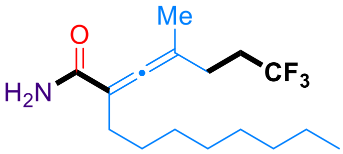

4y

^{1}H NMR (700 MHz, $CDCl_3$)

^{13}C NMR (176 MHz, $CDCl_3$)

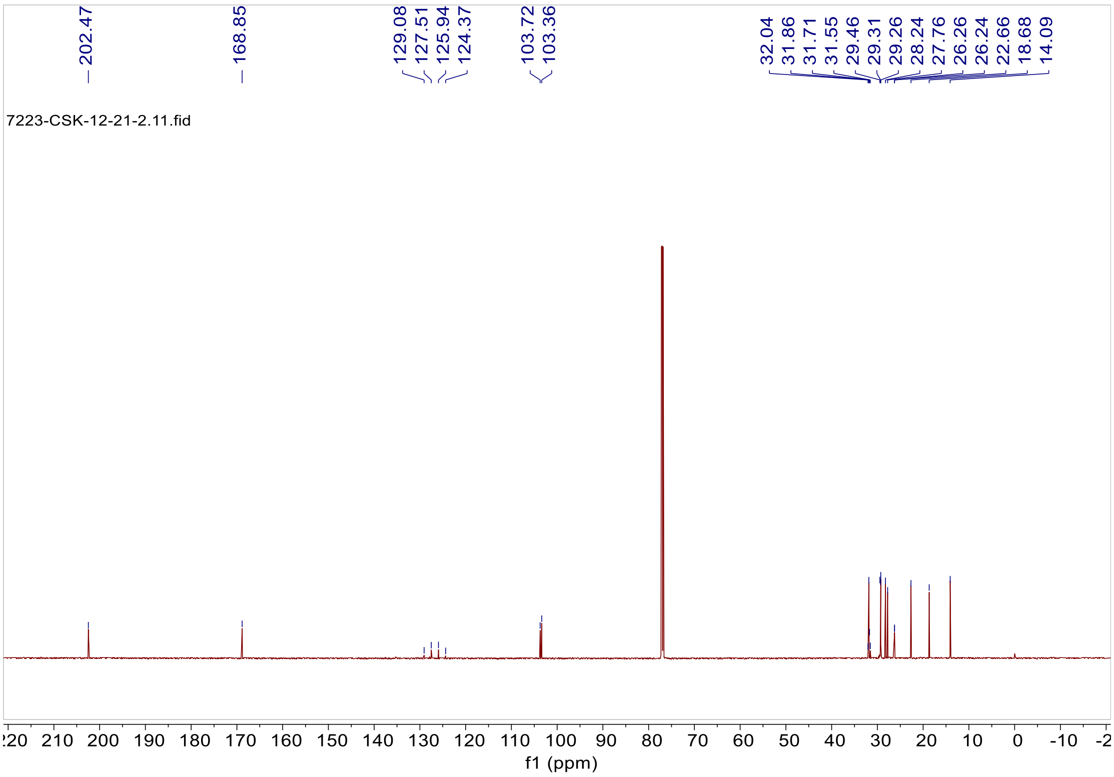

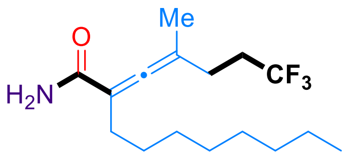

4y ^{19}F NMR (376 MHz, $CDCl_3$)

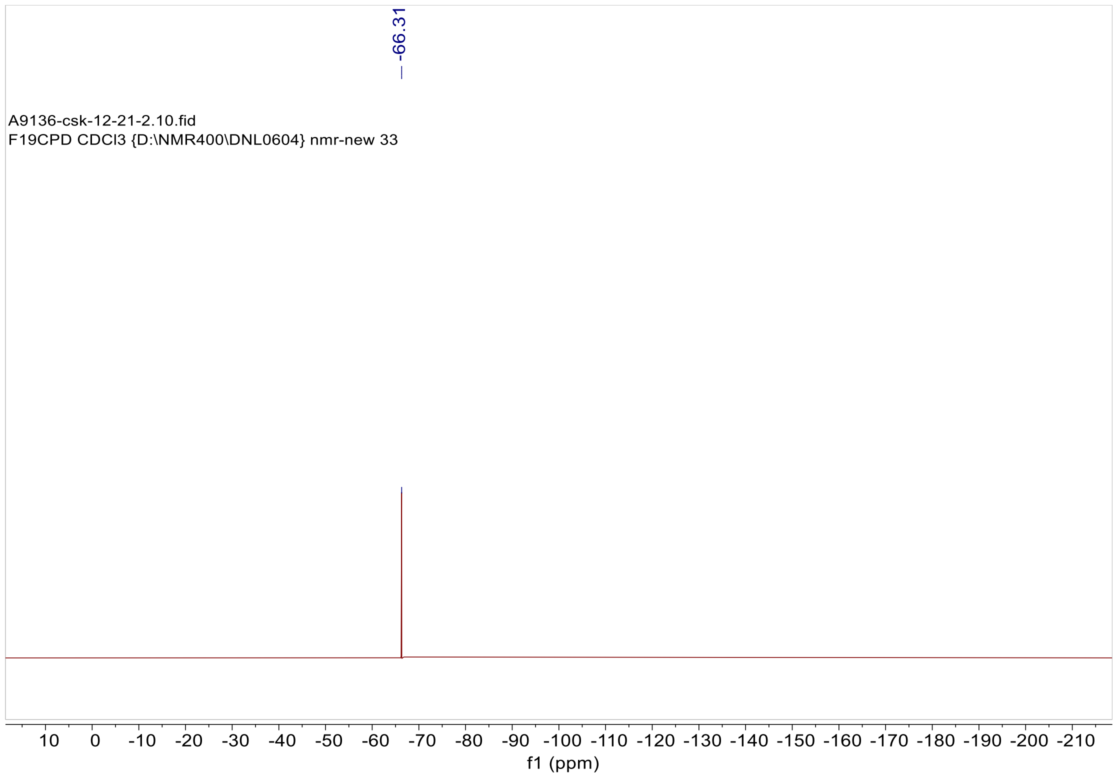

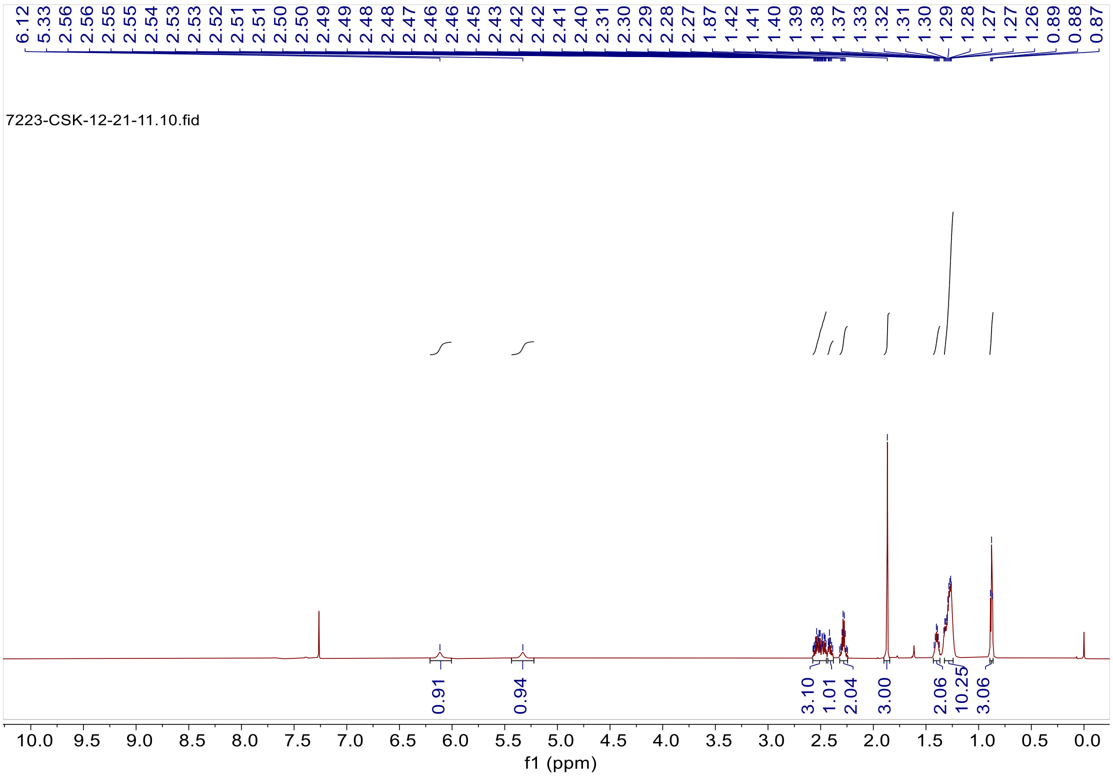

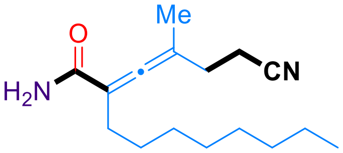

4z

^{1}H NMR (700 MHz, $CDCl_3$)
^{13}C NMR (176 MHz, $CDCl_3$)

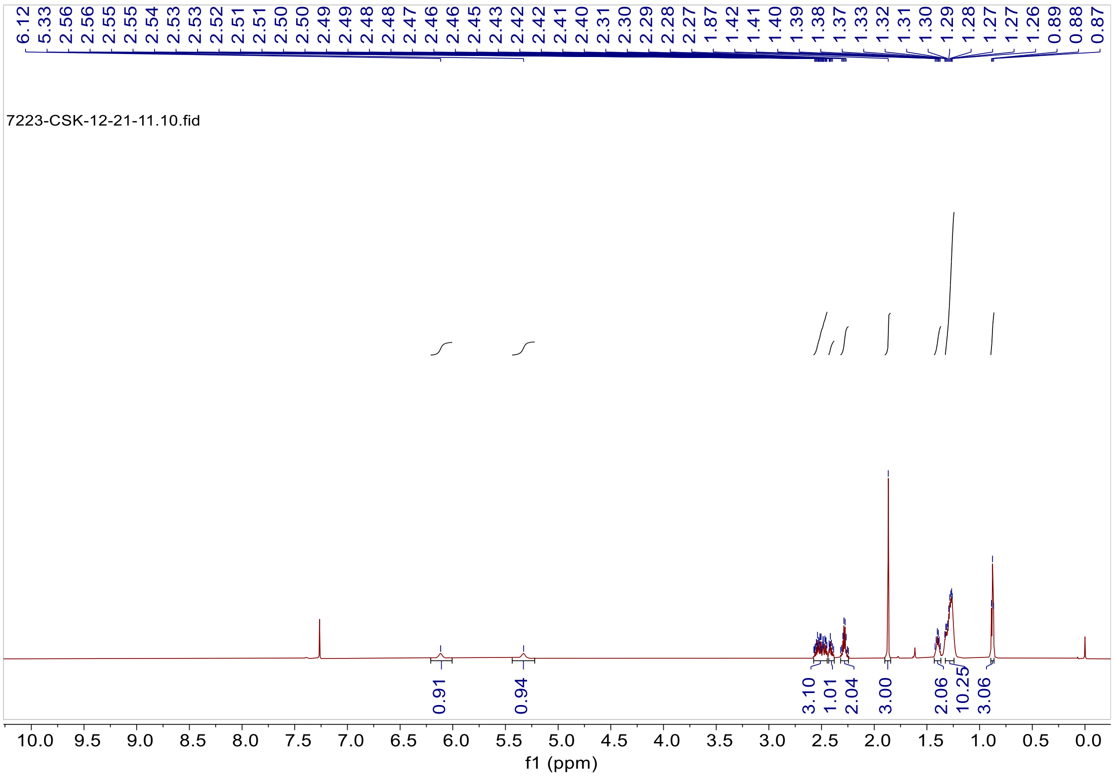

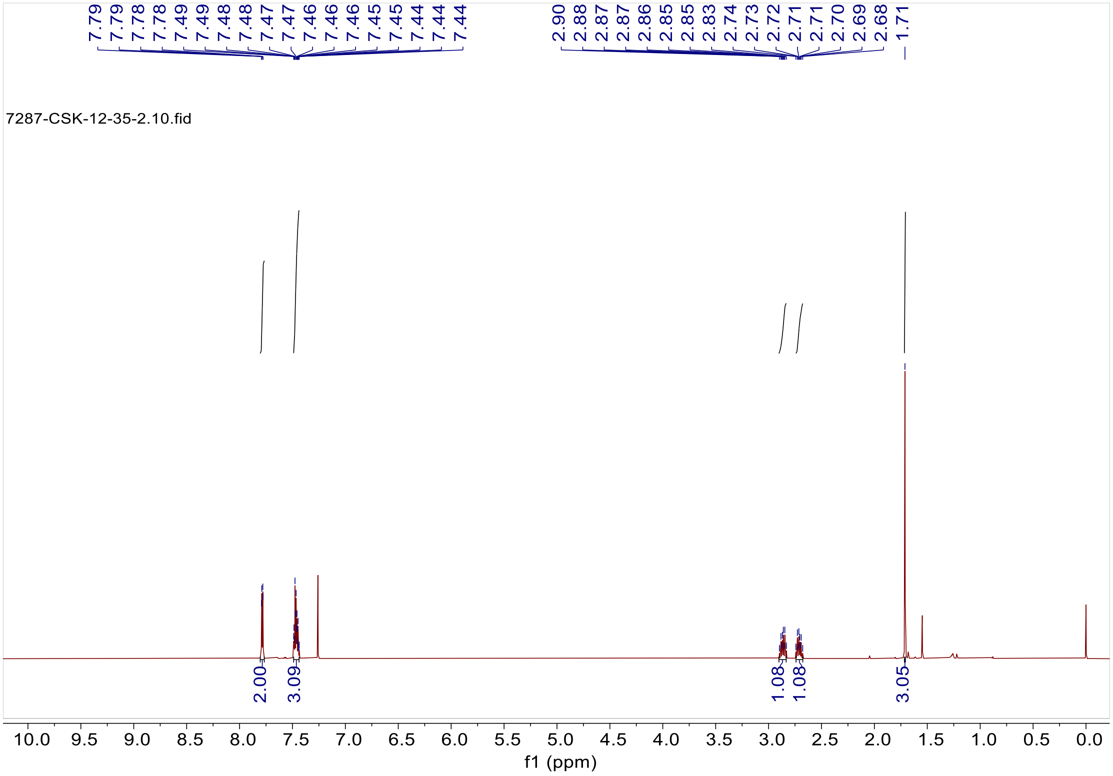

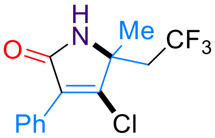

5a

^{1}H NMR (700 MHz, $CDCl_3$)
^{13}C NMR (176 MHz, $CDCl_3$)

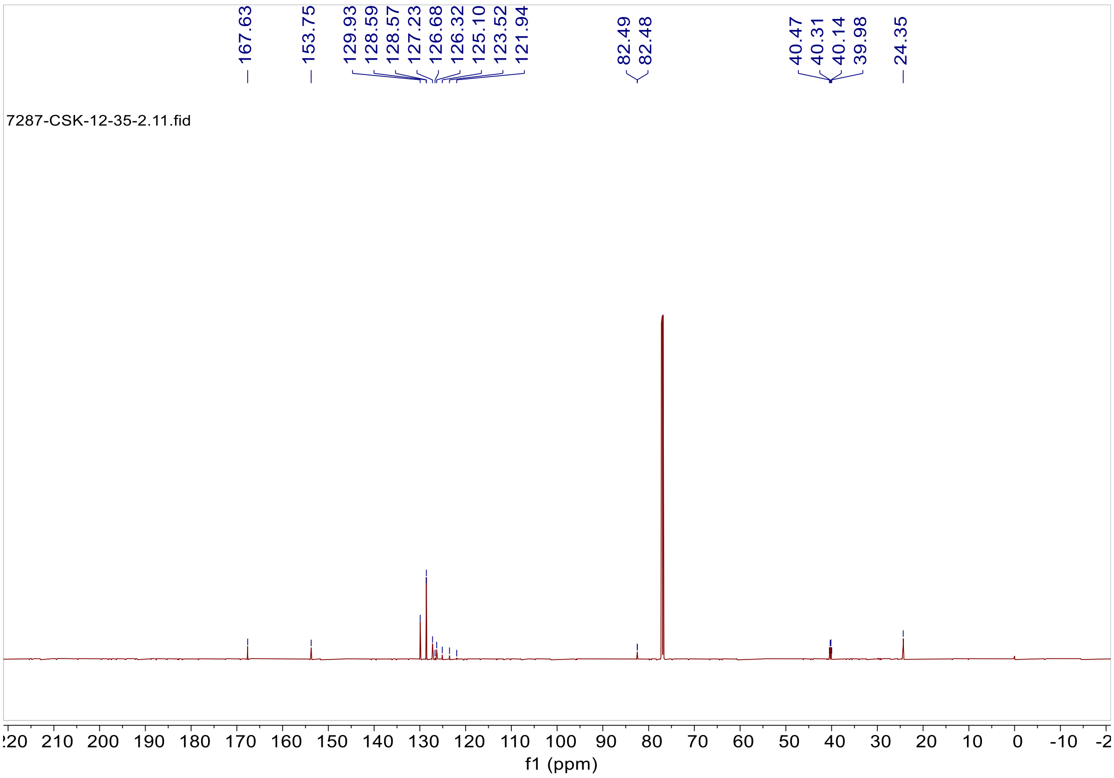

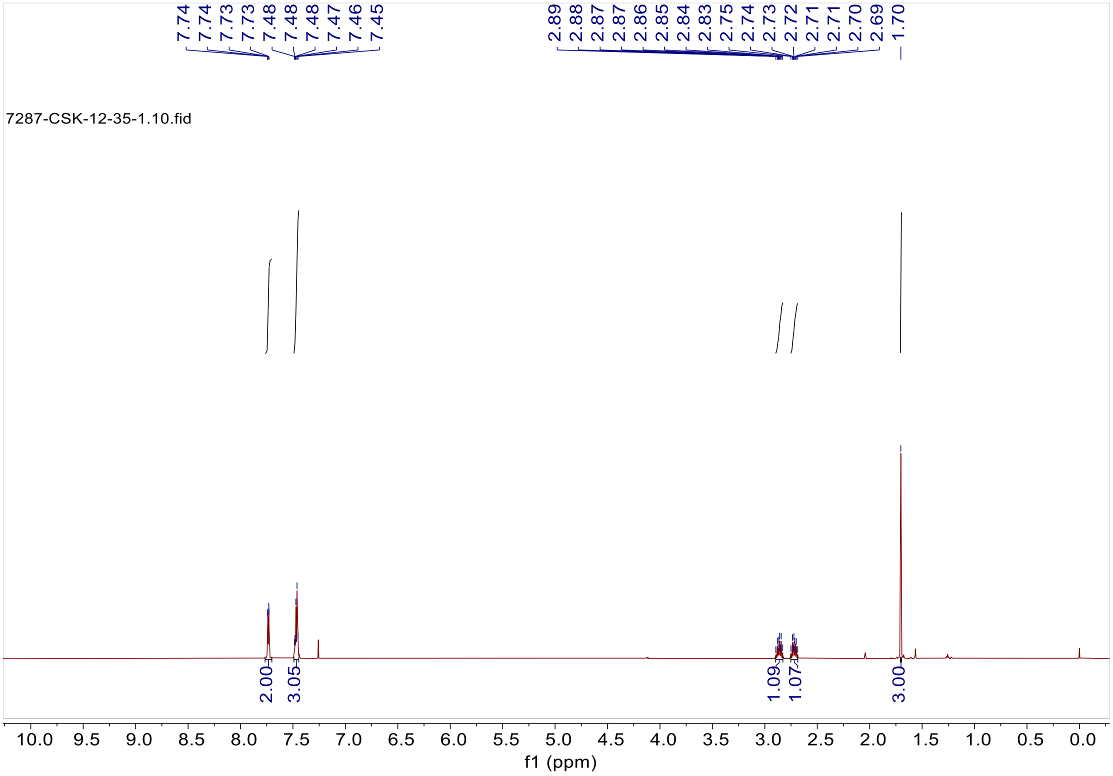

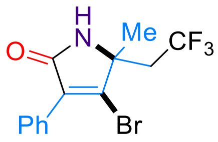

5b

^{1}H NMR (700 MHz, $CDCl_3$)
^{13}C NMR (176 MHz, $CDCl_3$)

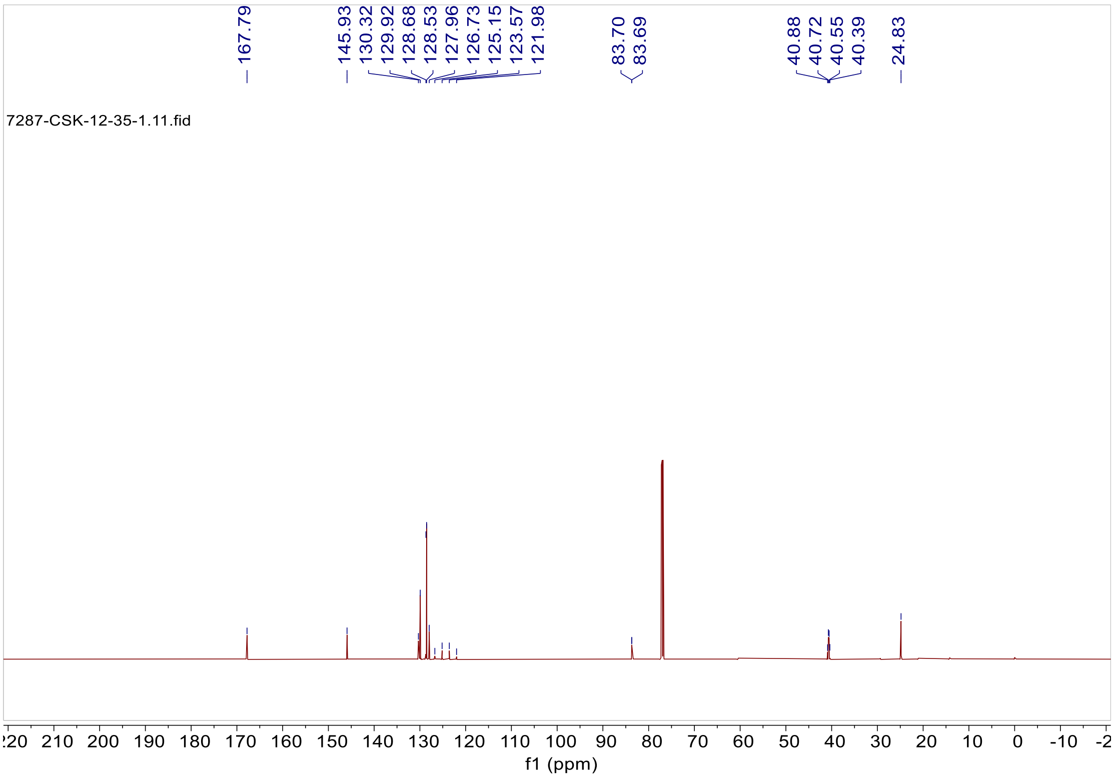

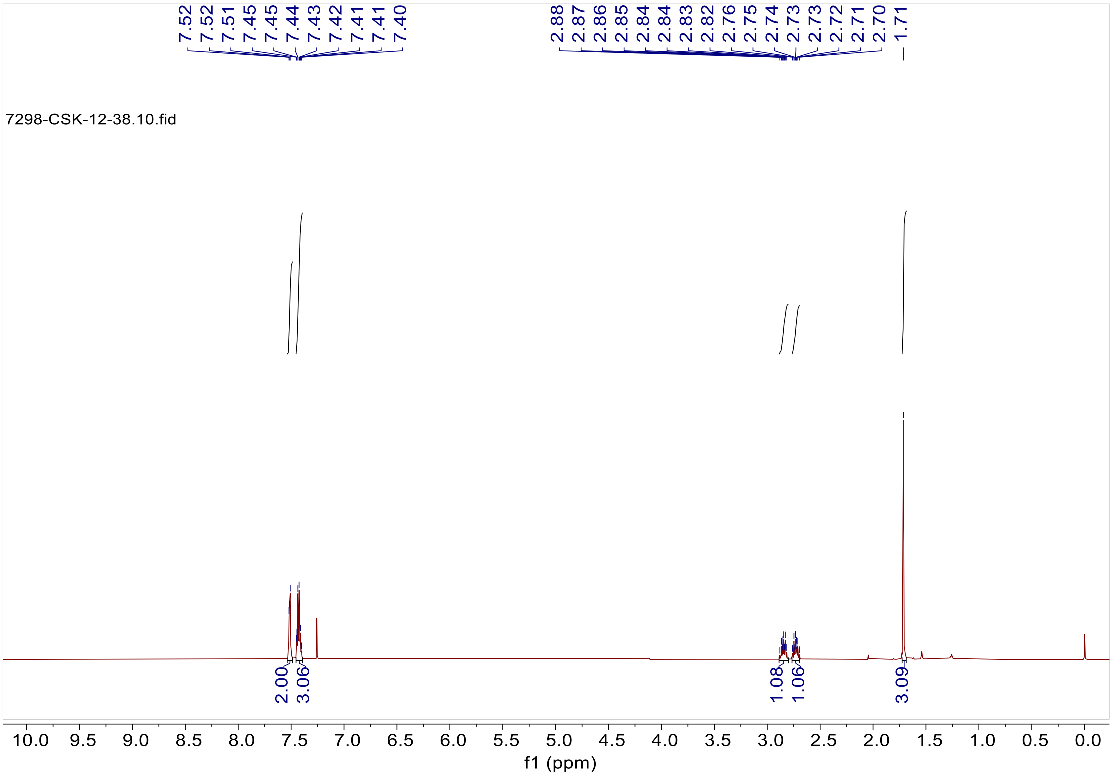

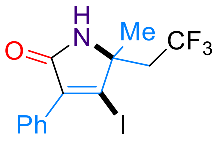

5c

^{1}H NMR (700 MHz, $CDCl_3$)
^{13}C NMR (176 MHz, $CDCl_3$)

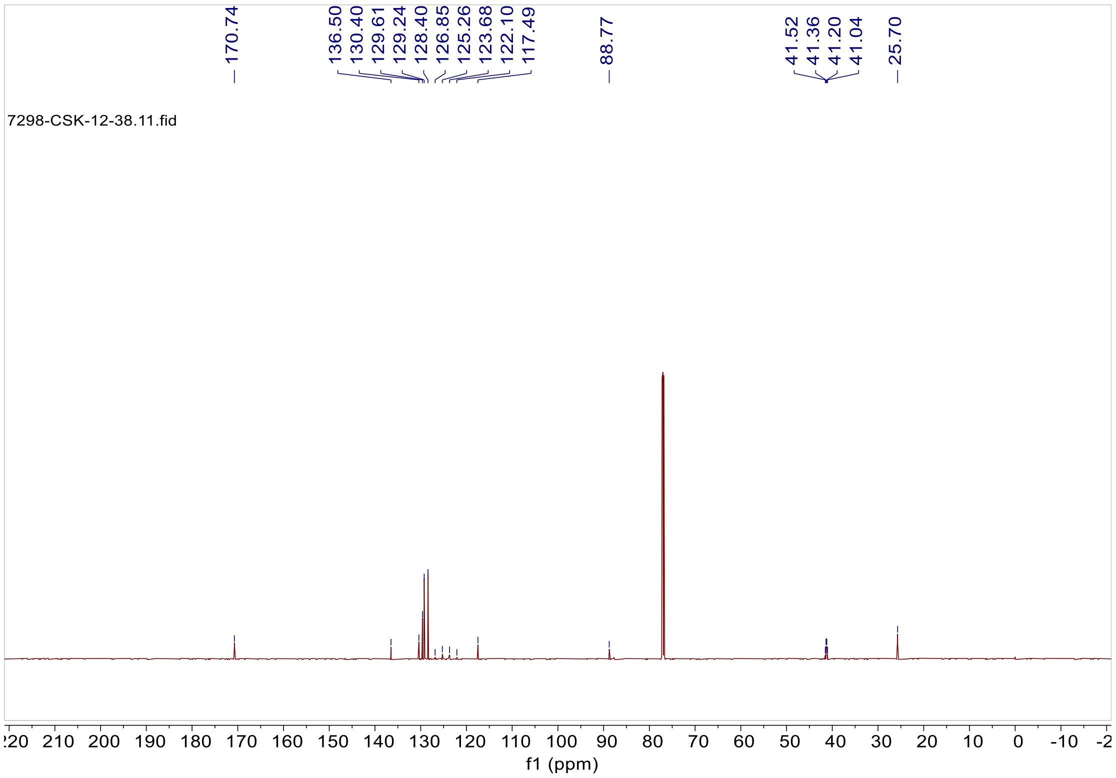

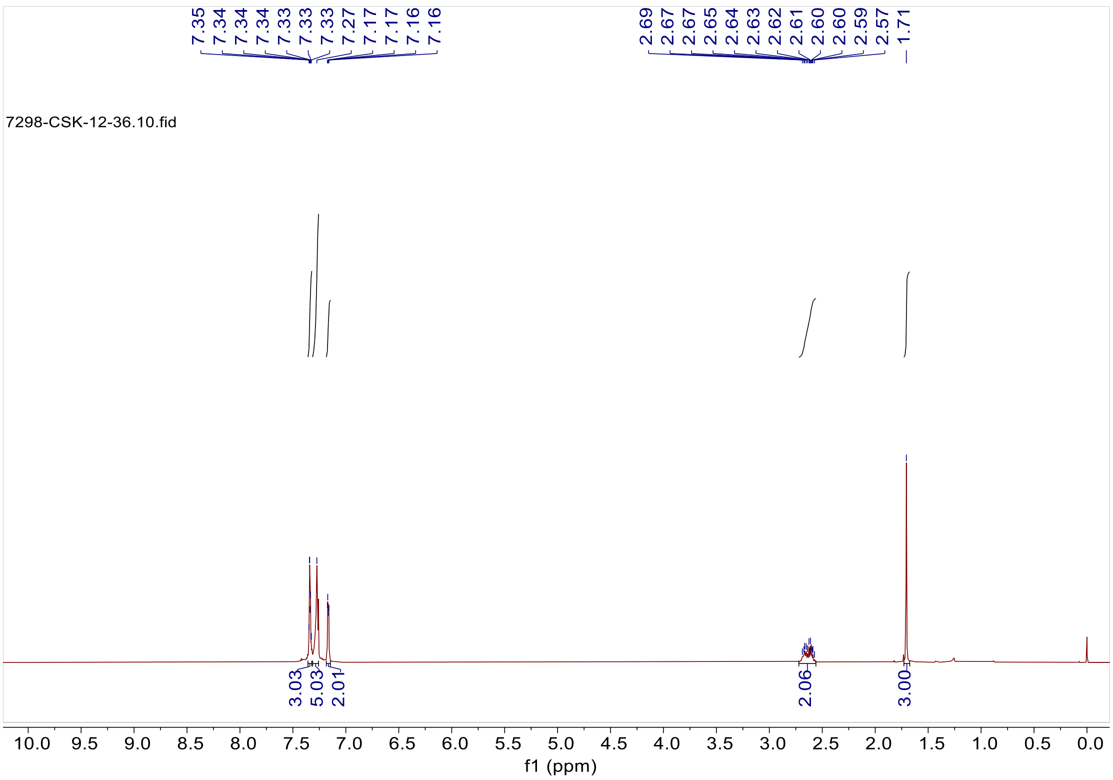

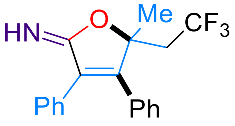

5d

^{1}H NMR (700 MHz, $CDCl_3$)

^{13}C NMR (176 MHz, $CDCl_3$)

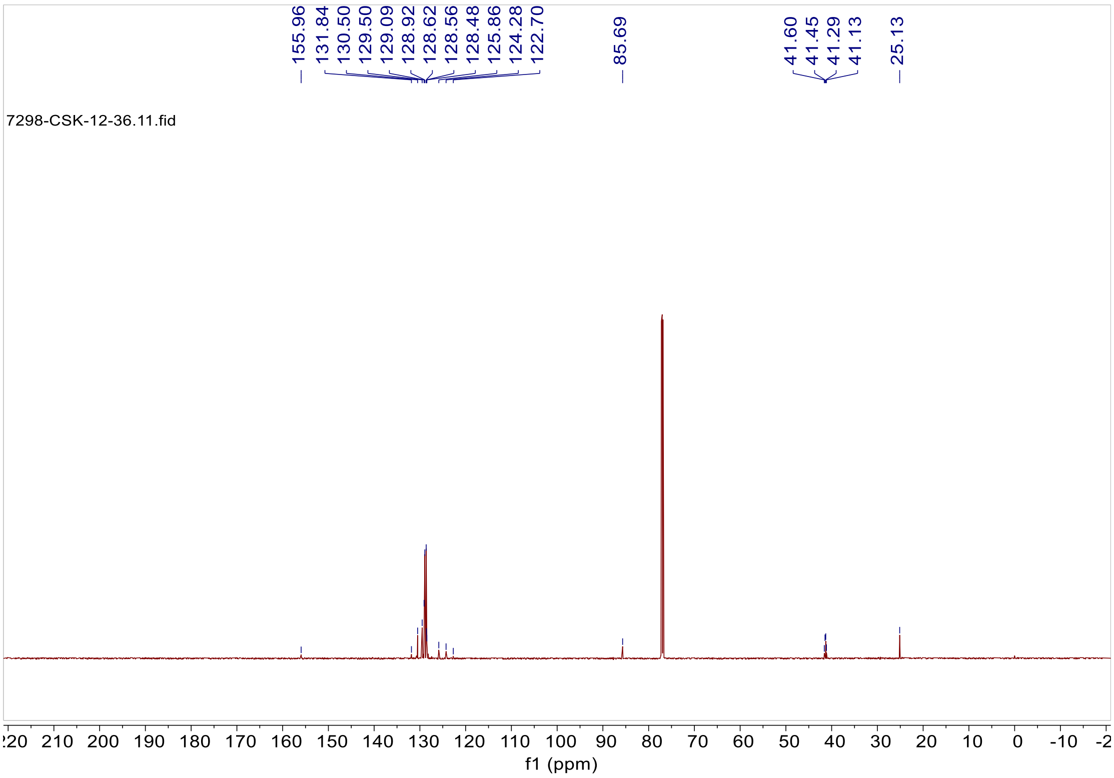

Supplement: Supplementary file 1 [file ol5c03213_si_001.pdf]
